# Unexpected patterns of segregation distortion at a selfish supergene in the fire ant *Solenopsis invicta*

**DOI:** 10.1186/s12863-018-0685-9

**Published:** 2018-11-07

**Authors:** Kenneth G. Ross, DeWayne Shoemaker

**Affiliations:** 1 Department of Entomology, University of Georgia, Athens, GA USA; 2 Department of Entomology and Plant Pathology, University of Tennessee, Knoxville, TN USA

**Keywords:** Colony social organization, Meiotic drive, Segregation distortion, Selfish genetic element, Supergene, Transmission ratio distortion

## Abstract

**Background:**

The *Sb* supergene in the fire ant *Solenopsis invicta* determines the form of colony social organization, with colonies whose inhabitants bear the element containing multiple reproductive queens and colonies lacking it containing only a single queen. Several features of this supergene — including suppressed recombination, presence of deleterious mutations, association with a large centromere, and “green-beard” behavior — suggest that it may be a selfish genetic element that engages in transmission ratio distortion (TRD), defined as significant departures in progeny allele frequencies from Mendelian inheritance ratios. We tested this possibility by surveying segregation ratios in embryo progenies of 101 queens of the “polygyne” social form (3512 embryos) using three supergene-linked markers and twelve markers outside the supergene.

**Results:**

Significant departures from Mendelian ratios were observed at the supergene loci in 3–5 times more progenies than expected in the absence of TRD and than found, on average, among non-supergene loci. Also, supergene loci displayed the greatest mean deviations from Mendelian ratios among all study loci, although these typically were modest. A surprising feature of the observed inter-progeny variation in TRD was that significant deviations involved not only excesses of supergene alleles but also similarly frequent excesses of the alternate alleles on the homologous chromosome. As expected given the common occurrence of such “drive reversal” in this system, alleles associated with the supergene gain no consistent transmission advantage over their alternate alleles at the population level. Finally, we observed low levels of recombination and incomplete gametic disequilibrium across the supergene, including between adjacent markers within a single inversion.

**Conclusions:**

Our data confirm the prediction that the *Sb* supergene is a selfish genetic element capable of biasing its own transmission during reproduction, yet counterselection for suppressor loci evidently has produced an evolutionary stalemate in TRD between the variant homologous haplotypes on the “social chromosome”. Evidence implicates prezygotic segregation distortion as responsible for the TRD we document, with “true” meiotic drive the most likely mechanism. Low levels of recombination and incomplete gametic disequilibrium across the supergene suggest that selection does not preserve a single uniform supergene haplotype responsible for inducing polygyny.

**Electronic supplementary material:**

The online version of this article (10.1186/s12863-018-0685-9) contains supplementary material, which is available to authorized users.

## Background

Selfish genetic elements are heritable entities that spread in populations despite having no or even negative fitness consequences for the host organism [1–4]. Among the numerous types known, selfish elements that bias their transmission to progeny through gametogenesis or early offspring ontogeny are attracting increased attention. Transmission ratio distortion (TRD), defined as significant departures in progeny allele or haplotype frequencies from expected Mendelian inheritance ratios [5–7], appropriately refers to cases where it is unknown whether distortion arises in the products of meiosis (gamete selection) or fertilization (differential zygote/embryo survival) [8–10]. Segregation distortion, which refers to TRD manifested around gametogenesis (also called meiotic drive [11, 12], cf. [13]), takes two general forms [14]. “True” meiotic drive occurs when segregation distortion is confined to events associated with meiosis, such that drive elements subvert the process of fair chromosome segregation to be preferentially included in the functional products of meiosis, the gametes [3, 15, 16]. This form typically is tied to female gametogenesis because inherently asymmetric meioses in females lead to production of functional (egg or ovule) and nonfunctional (polar body) products, generating opportunities for homologs to compete for inclusion in gametes. The other common form of segregation distortion, “gamete killing” drive, occurs post-meiotically but before fertilization and features destruction or functional disruption of gametes lacking the drive element, as orchestrated by the element [3, 8]. This form of distortion usually is associated with male gametogenesis, presumably because drive-induced loss of functional gametes normally entails lower fecundity costs to males than females [6, 17, 18]; while male gamete killing drive appears to be the most common form of segregation distortion, ascertainment bias may make it appear so simply because it usually produces altered sex-ratios and therefore is readily detected [6].

Transmission ratio distortion increasingly is viewed as an important evolutionary force driving such diverse phenomena as the origin of meiotic asymmetries in oogenesis [19], the dynamics of chromosomal architecture [20], mating system evolution [6, 21–23], speciation [3, 9, 24], and extinction [25]. Notably, though, TRD appears to have only a sporadic taxonomic distribution and often seems poorly developed or rare in those wild populations in which it has been reported [5, 6]. Several explanations have been offered for this: (i) non-Mendelian drive loci may rapidly spread to fixation, precluding their detection as polymorphisms within populations [7, 18]; (ii) recombination may disrupt selfish drive complexes to generate haplotypes incapable of producing segregation distortion [26]; (iii) the segregation advantage of drive elements is strongly opposed by genetic elements on the homologous chromosome, creating gene-level selection for suppressor alleles that curtail distortion [3, 12, 18, 27, 28]; and (iv) reductions in fecundity due to recruitment of linked deleterious factors induce negative selection at the organismal level — this favors the spread of unlinked suppressors throughout the genome that can act at the gene level to counteract drive during gametogenesis or induce changes in mating systems (e.g., mate discrimination, multiple mating) or other organismal traits that mitigate drive prevalence [12, 17, 22, 27, 29, 30].

An additional explanation for the apparent low incidence of segregation distortion and other forms of TRD is suggested by the fact that relatively few studies have surveyed wild populations specifically for evidence of subtle drive, including detailing the nature and extent of variation among progenies [5, 9, 10, 24, 31–33]. This task is made all the more challenging when such distortion does not involve sex chromosome drive, the phenotypic manifestations of which (deviant sex ratios) are readily visible [6, 27]. In addition to revealing the taxonomic distribution and prevalence of TRD, broad surveys of drive and its variation in wild populations can illuminate genetic features such as the relationship between drive locus structure and strength of distortion [26, 34], additive effects of linked drive elements and enhancers [6, 12, 17], epistatic interactions between drive elements and unlinked suppressors [7, 17, 35–37], and the impact of drive elements on fundamental structural genomic components such as inversions, duplicate genes, and centromeric expansions [7, 33, 38].

The fire ant *Solenopsis invicta* is a highly eusocial insect that has emerged as an important model for social evolution and is of interest with regard to the potential for TRD, including segregation distortion. This species, which is an invasive pest in the southern USA and elsewhere [39], displays naturally occurring variation in a fundamental, ecologically important social trait, the number of reproductive (egg-laying) queens per colony [40]. Colonies of the “monogyne” social form are headed by a single such queen, whereas colonies of the “polygyne” form contain multiple queens. The two forms differ not only in colony queen number but as well in many other individual-level, colony-level, and life-history traits [41]. Remarkably, variation in colony queen number is completely associated with allelic variation at a single Mendelian factor marked by the gene *Gp-9* [42, 43]. Colonies of the monogyne form in its invasive range include only inhabitants bearing the *B* coding-sequence variant (allele) of *Gp-9* (in its native range, additional similar variants occur and collectively are termed “*B*-like” alleles). In contrast, colonies of the polygyne form always additionally include some inhabitants bearing the alternate *b* allele (or related "*b*-like" alleles in the native range), and all polygyne reproductive queens possess such an allele. Worker fire ants regulate colony social organization by tolerating and nurturing reproductive queens collectively deemed acceptable and destroying those deemed unacceptable. This fact led to the hypothesis — verified experimentally [44, 45] — that the presence in a fire ant colony of even a low frequency of workers with the *b* allele is both necessary and sufficient to elicit acceptance of multiple queens (each bearing the *b* allele). Conversely, colonies lacking such workers entirely (monogyne colonies) tolerate only a single queen that invariably also lacks the *b* allele (i.e., monogyne queens are always *BB* homozygotes) [46].

Linkage mapping revealed that the *b* allele of *Gp-9* occurs within a large chromosomal segment, termed the *Social b* (*Sb*) supergene, that undergoes limited recombination with its homolog [47, 48]. This region corresponds to some 60% of the sequence of one of the 16 *S. invicta* chromosomes (~ 13 Mb) and includes several hundred genes, suggesting that many genes associated with the *Gp-9*^*b*^ allele in the supergene may be responsible for the range of phenotypic differences observed between the social forms [47, 49–51]. Suppression of recombination presumably is explained by the acquisition of one large (~ 9 Mb) and two smaller inversions [50, 51], along with substantial repetitive sequence, by the *Sb* supergene [49]. The corresponding region in the homologous (*SB*) chromosome 16 undergoes free recombination, but there likely is no effective recombination between *Sb* haplotypes in invasive populations because homozygous queens invariably die before becoming reproductively active [42, 52, 53]. Minimal recombination, along with other potential factors such as historical selective sweeps [54, 55] and a pronounced genetic bottleneck during founding of the US population [56], appear to have resulted in extremely low genetic variation among supergene sequence variants in the invasive range [48].

The *Sb* supergene shares several features in common with known segregation distorters, raising the prospect that it may also act as an element favoring its own transmission during oogenesis. First, suppression of recombination over the large *Sb* region is similar to that characterizing diverse segregation distorters, which typically comprise multilocus systems whose components are held in strong linkage disequilibrium by physical linkage, inversions, pericentric locations, or other features that minimize recombination [17, 27, 57, 58]. Second, the *Sb* supergene contains deleterious mutations, the diverse effects of which include impairment of the reproductive potential of heterozygous queens, effective lethality in homozygous queens, reduction in longevity in homozygous workers, and reduction in sperm counts in (hemizygous) males [43, 59] — such incorporation of deleterious alleles that negatively affect organismal fitness is a hallmark of known segregation distorter gene complexes [13, 17, 21, 36, 58, 60, 61]. Third, *Sb* evidently is a sink for transposable elements in fire ants [49, 62], paralleling the common accumulation of these and other types of repetitive DNA at segregation distortion loci [4, 7, 17, 34, 63]. Fourth, the *Sb* supergene likely encompasses the centromere of chromosome 16, which, like all centromeres in *S. invicta* [64], is exceptionally long and possibly involved in centromere-mediated competition between homologs for microtubule binding sites [14].

A final consideration bolstering the possibility that *Sb* behaves as a segregation distorter is the fact that it displays other selfish genetic attributes. For instance, the supergene appears to be over-represented by about 50% among female brood that develop into queens compared to those that develop into workers [65]; this suggests that *Sb* may bias the developmental trajectory of a host fire ant larva into the reproductive rather than sterile caste and thus promote its own perpetuation via delayed ontogenetic manipulation (a unique form of TRD). Also, the supergene underlies a “green-beard” phenomenon, characterized by an extreme and rarely observed strategy of some selfish genetic elements. Specifically, fire ant workers bearing the *Sb* element preferentially attack and destroy queens that lack it (*SB*/*SB*) when such queens are introduced into polygyne colonies, whereas workers lacking the element are under-represented among the attackers [66, 67]. This green-beard mode of facultative harming behavior [68] favors persistence of the *Sb* haplotype in the polygyne form despite its deleterious effects at the organismal level.

In this study, we investigated the possibility that the *Sb* supergene in polygyne *S. invicta* is involved in segregation distortion during gametogenesis. We note that because males of this species normally are haploid, such TRD necessarily is confined to reproducing queens and, most likely, the events surrounding oogenesis. To distinguish segregation distortion in mothers from TRD that occurs at the zygote/embryo stages, the ideal strategy is to genotype unfertilized eggs directly, a technically daunting task. Alternatively, the fertilization products (zygotes / embryos) can be genotyped as soon as feasible after fertilization to allow reliable genotyping yet minimize any possibility of genotype-biased differential mortality due to endogenous causes or discrimination by parents/alloparents. Thus we examined segregation ratios of *Sb* in progenies of young diploid (sexually produced) embryos produced by mated heterozygous (*SB*/*Sb*) polygyne queens. For comparative purposes, we also examined segregation ratios in these same embryos at 14 polymorphic microsatellite loci. In addition to documenting evidence of distortion at a genetic element that is a candidate for such selfish behavior in a wild population, we closely examined the extent of variation among progenies (different genetic backgrounds) in the hope of gaining insights into the genetic architecture of distortion and its suppression. Although our design does not allow us to definitively identify the stage at which distortion occurs, several lines of evidence point to gametogenesis; hence, we hereafter use the terms (meiotic) drive, segregation distortion, and transmission ratio distortion largely interchangeably.

## Methods

### Colony collection, rearing, and queen classification

Twelve large polygyne nests of *S. invicta* were collected in northeastern Georgia, USA (see Table 1; Additional file 1: Table S1). Colony inhabitants were removed from the soil in the laboratory [69] then transferred to special nests held in plastic trays in a rearing room (see Additional file 2: Text S1 for rearing details). Several dozen wingless (presumably reproductive) queens from each polygyne colony were isolated individually in small broodless fragments of their parent colony [70]; after four weeks the brood patterns in the fragments allowed us to distinguish unmated from mated queens (worker brood absent or present, respectively). Among the latter, 113 were held for further study, 101 of which were used to produce progenies whose *Gp-9* and multilocus microsatellite genotype distributions were studied (Additional file 3: Figure S1).

**Table 1:** Study sample sizes and summary data on transmission ratio distortion (TRD) in polygyne *Solenopsis invicta.*

Sample sizes					
Number of nests from which polygyne study queens					12
Number of queens genotyped					113
Number of queens producing study progenies^a^					101 (8.4 ± 1.7, 4–11)
Number of loci genotyped in queens / progenies					15
Mean number of progenies genotyped per locus^b^					93.1 (40–101)
Number of embryos with DNA extracted					3621
Number of embryos successfully genotyped					3512
Number of presumed non-embryonated eggs^c^					109 (0.030)
Number of embryos genotyped per progeny^d^				Locus	Mean no.
				*Bertha*	32.7
				*C27*	32.6
				***C294***	32.1
				*C536*	32.3
				*cassidy*	31.7
				***Gp-9***	34.5
				*i_109*	33.6
				*i_114*	32.6
				*i_120*	31.9
				***i_126***	33.1
				*i_129*	30.8
				*red_ant*	34.0
				*Sol-42f*	32.9
				*Sol-49*	32.5
				*sunrise*	33.9
Summary TRD data					
Locus^e^	Number of segregating progenies^f^	Number of embryos genotyped in segregating progenies^g^	Mean *k*^h^	Proportion of segregating progenies with *k >* 0.65^i^	Proportion of segregating progenies with *k* significantly > 0.5^j^
*red_ant*	12 (0.30)	417 (34.8)	0.568 (0.549–0.589)	0.000	0.000 (−)^k^
*C27*	35 (0.36)	1153 (32.9)	0.558 (0.547–0.571)	0.000	0.000 (0.026, 0.000)^l^
*C536*	80 (0.81)	2578 (32.2)	0.564 (0.555–0.573)	0.038	0.025 (0.025, 0.000)
*Sol_42f*	77 (0.77)	2542 (33.0)	0.566 (0.555–0.577)	0.065	0.039 (0.040, 0.000)
*i_114*	72 (0.71)	2344 (32.6)	0.565 (0.556–0.576)	0.056	0.042 (0.044, 0.000)
*sunrise*	50 (0.50)	1700 (34.0)	0.581 (0.566–0.597)	0.100	0.060 (0.063, 0.000)
*cassidy*	65 (0.70)	2065 (31.8)	0.568 (0.555–0.582)	0.062	0.062 (0.063, 0.000)
*i_109*	81 (0.80)	2712 (33.5)	0.572 (0.560–0.585)	0.074	0.074 (0.074, 0.000)
*i_120*	74 (0.74)	2392 (32.3)	0.575 (0.563–0.587)	0.122	0.081 (0.080, 0.000)
*Sol_49*	76 (0.75)	2464 (32.4)	0.577 (0.565–0.590)	0.092	0.092 (0.094, 0.000)
*Bertha*	67 (0.66)	2180 (32.5)	0.581 (0.568–0.595)	0.134	0.104 (0.106, 0.034)
*i_129*	29 (0.30)	851 (29.3)	0.579 (0.556–0.605)	0.138	0.138 (0.140, 0.034)
***Gp-9***	101 (1.0)	3493 (34.6)	0.584 (0.572–0.595)	0.168	0.168 (0.166, **0.069**)
***C294***	61 (0.98)	1955 (32.0)	0.589 (0.572–0.607)	0.197	0.180 (0.182, **0.069**)
***i_126***	85 (0.84)	2808 (33.0)	0.590 (0.575–0.605)	0.235	0.200 (0.205, **0.103**)

The term “progeny” is used to refer to a group of diploid embryos that are the offspring of a single mated polygyne queen (i.e., a family of diploid siblings). Three of the15 loci studied are located within the *Sb* supergene (see Additional file 4: Table S2 for genomic coordinates of all loci); these supergene loci are highlighted in bold font

^a^These queens are a subset of the total 113 queens genotyped. Mean numbers of queens originating from each nest (±standard deviation and range) are in parentheses

^b^Ranges in parentheses

^c^Non-embryonated eggs fail to undergo embryogenesis and may serve a trophic function. Non- embryonated eggs as a proportion of total eggs studied is in parentheses

^d^Excludes complete genotyping failures in a progeny; loci are listed in alphabetical order

^e^Loci are arranged from lowest to highest proportions of segregating progenies that feature *k* significantly > 0.5 (binomial test)

^f^Segregating progenies as proportions of total progenies genotyped are in parentheses

^g^Mean number of embryos genotyped per segregating progeny are in parentheses

^h^In this table, the parameter *k* is the proportion of gametes in segregating progenies carrying an allele present at frequencies >0.5 (unpolarized *k*). The mean as well as the two-tail 95% confidence intervals (in parentheses) are based on 1000 bootstrap replicates

^i^For the mean sample sizes of 32-33 embryos genotyped per segregating locus in this study, *k*=0.65 is a threshold above which segregation ratios generally depart significantly from 1:1 according to one-tailed exact binomial tests

^j^Observed proportions based on exact binomial tests; bootstrap/rarefaction estimates of the observed proportions, along with their lower one-tailed 95% confidence limits, are shown respectively in parentheses. Confidence limits greater than 0.05 (bold font) indicate significant TRD at a locus

^k^Number of segregating progenies was too low to conduct bootstrap analyses

^l^Bootstrap estimates assume that the next progeny sampled would have yielded a *k* value significantly > 0.05

### Collection of embryo progenies

Families (progenies) of diploid embryos were obtained from 101 mated mother queens to quantify transmission ratio distortion (TRD) (Table 1; Additional file 3: Figure S1; Additional file 2: Text S1). Queens were isolated in specimen cups with 2–3 adult workers for 12 h, after which each queen was removed and frozen at − 80 °C. Eggs laid by the queen were maintained with the workers for an additional 48 h; these eggs (technically, embryos within the egg coat) were then collected, transferred into a gelatin capsule, and immediately frozen at − 80 °C. The ages of collected embryos thus ranged from 48 h to 72 h post-oviposition. Eggs laid by queens in the 12 h isolation period of this experiment typically numbered in the hundreds, too numerous to count and track over the ensuing 48 h.

To remedy this lack of information on the fate of embryos under such conditions, we conducted a set of supplementary tests in which we examined the aptitude of similarly sized groups of adult workers to successfully maintain viable eggs/embryos for 48 h. Single reproductive polygyne queens were held in a small petri dish unit for 12-24 h, at which point they were removed and the eggs they laid were counted. Two or three workers from the queen’s colony of origin were then placed in each unit. After 48 h, all intact, evidently viable embryos in the unit were counted. Ten queens from each of four source colonies produced a total of 1637 eggs used in these tests (mean = 40.9 eggs/queen).

### DNA extraction and *Gp-9* / microsatellite genotyping to quantify TRD

Frozen embryos (36 per progeny) were transferred individually into single assay-plate wells for genomic DNA extraction (see Additional file 2: Text S1 for details). After accounting for rare losses, a total of 3621 embryos were successfully extracted. DNA also was extracted from the heads of each of the 101 progeny mother queens, as well as twelve additional mother queens from the same source colonies whose progenies were not studied. A modified multiplex PCR procedure [71] was used to score genotypes of individual embryos and queens at the *Gp-9* gene (Additional file 2: Text S1). Primers for this assay amplify all *Gp-9* allele *B* and allele *b* coding-sequence variants known from the US range of *S. invicta* [39, 72]; thus, all three major-allele genotypes (*BB*, *Bb*, *bb*) could be scored directly by running out the PCR products in agarose gels. Total volumes of the undiluted PCR amplicons were run out, stained with ethidium bromide, and visualized under UV light. Any of the embryos that yielded weak or no detectable *Gp-9* PCR products using the above method, but for which microsatellites could be scored (below), were subjected to a TaqMan qPCR (Applied Biosystems) assay [73] in order to definitively confirm or assign *Gp-9* genotype. Multilocus genotypes at 14 microsatellite loci (Additional file 4: Table S2) were generated in three separate multiplex PCR reactions (Additional file 2: Text S1) and scored from sequence chromatograms with the aid of GENEMARKER software (SoftGenetics). Two of the microsatellites (*C294*, *red_ant*) were genotyped only for subsets of progenies because of their development during the course of the study or low variation, respectively (see Table 1; Additional file 1: Table S1 for sample sizes). Also, two of the microsatellites (*C294*, *i_126*) were found during the course of the study to be linked to *Gp-9* and contained within or located near the *Sb* supergene inversions (see below; Additional file 5: Figure S2). The 109 eggs for which neither *Gp-9* nor any of the microsatellites could be scored are assumed to be “non-embryonated eggs,” which look normal for several days post-oviposition but fail to undergo embryogenesis (are inviable [74]) and occasionally are laid by mated polygyne *S. invicta* queens [75].

We note that artifactual errors in genotype scoring caused by allelic drop-outs (non-amplification of one allele in heterozygotes) or by maternal DNA contamination associated with the small amounts of genomic DNA in our study embryos are highly unlikely. Careful examination and analyses of the patterns of multilocus genotypes in progenies typically fathered by a single haploid male, as we performed, would readily reveal evidence for such artifacts (see Additional file 2: Text S1). Moreover, spurious embryo genotype calls would mask the differences in frequencies of significant TRD that we observed between supergene and non-supergene loci, would erode the strong congruence we observed between measures of recombination and gametic disequilibrium, and would undermine the concordant patterns of segregation distortion we found among the three supergene loci (see below, Additional file 2: Text S1).

### Data analyses

The multilocus *Gp-9* and microsatellite genotypes of individual diploid offspring embryos were used to infer the social chromosome and marker-locus phased haplotypes of the eggs giving rise to each embryo. Such inference was simplified because the multilocus genotypes of all mother queens were known, fertile males are almost always haploid (but see [76] and below for exceptions), polygyne reproductive queens invariably are *Gp-9* heterozygotes in introduced *S. invicta* [43, 77], and queens of this species generally mate with single, or rarely two, males ([78], see also [59] and below). Using this information, it was straightforward to reconstruct the multilocus phased haplotypes of each mother queen, her eggs, and her mate(s). The availability of phased haplotypes for several thousand individuals derived from a wild population facilitated robust estimation of recombination fractions and gametic disequilibrium to complement our analyses of segregation distortion.

Allele frequencies and expected heterozygosity (*H*_exp_) were estimated for all 15 study loci from 113 mother queens and 109 of their male mates using GENEPOP ON THE WEB 4.2 [79, 80]. Pairwise genetic relatedness coefficients (*r*) between each progeny-yielding mother queen and her mate(s), as well as between pairs of nestmate queens, were estimated by the maximum likelihood method of Huang et al. [81] using the program POLYRELATEDNESS V1.5 and excluding the three supergene-linked loci; means and 95% confidence intervals (CIs) for *r* were obtained from 1000 bootstrap replicates over all pairs, a procedure adopted in light of the absence of colony-level effects on queen relatedness (Additional file 6: Text S2). Associations between *r* and congruence in deviations from Mendelian ratios at the supergene were examined for pairs of nestmate queens using a resampling method combined with non-parametric Spearman correlation tests (Additional file 2: Text S1); congruence in queens’ segregation ratios was represented by the difference in their *k* values (∆*k*), with *k* the proportion of gametes with the supergene-linked allele represented in a progeny. The fixation index *F*_ST_ was calculated by the method of Weir and Cockerham [82] as a measure of genetic differentiation between queens and their mates using the program GENEPOP and again excluding the supergene-linked loci. Exact probabilities that the observed genotype frequencies at the 15 study loci conformed to Hardy Weinberg equilibrium (HWE) were calculated for the 113 mother queens using a Markov chain method implemented in GENEPOP; values of the inbreeding coefficient *F*_IS_ for these same individuals were calculated by the method of Weir and Cockerham [82] using the same program.

Maximum likelihood estimates of the pedigree recombination frequency (recombination fraction; *c*) between each pair of marker loci were obtained for all jointly segregating progenies by directly calculating the ratio of the number of recombinant to the total number of gametes (eggs), with recombinants considered to be the least frequent of the two types [83]. A likelihood ratio test was used to test the null hypothesis that *c* = 0.5 (free recombination occurs) in each instance [84]. The standard error of the sample mean calculated across progenies for each locus pair was used to estimate the 95% confidence intervals (CIs) using the standard normal deviate. Because of the small number of progenies segregating for *red_ant*, this marker was included in these analyses only when ten or more jointly segregating progenies were available (four of 14 possible locus pairs).

Estimates of the gametic disequilibrium coefficient D* were obtained for locus pairs in progeny embryos and in the haploid male mates of queens that produced study progenies using the program ARLEQUIN v 3.5.2.2 [85]. For embryos, D* was calculated from the mother queen’s egg haplotypes (phase known) represented in embryos. Because males are haploid, the haplotypes are inherently phased. To avoid bias due to family structure in the embryos (two-locus Wahlund effect; [86]), a single multilocus egg haplotype was randomly sampled per progeny, the D* value was calculated, and means of the values from 100 resampling interations were taken as the parameter estimates. Exact test probabilities associated with the D* value, also obtained with ARLEQUIN, were corrected for multiple comparisons within each class (eggs, males) using the Holm-Bonferroni sequential method [87]. Gametic disequilibrium could not be evaluated for the locus pair *C294*/*red_ant* because no progenies were genotyped at both markers.

Segregation ratios for gametes bearing drive elements typically are represented by the parameter *k*, the proportion of gametes carrying the drive allele; thus, values of *k* are expected to vary from 0.5 (Mendelian [1:1] segregation) to 1.0 (complete segregation distortion) [12, 17]. However, in cases of reversal-of-drive (negative transmission ratio distortion) or for loci lacking drive alleles, *k* can be less than 0.5, in the latter case depending solely on the identity of the focal allele. Therefore, for some segregation analyses in which we wished simply to compare the average magnitude of distortion among all the study loci (without regard to specific alleles), we set *k* as the proportion of gametes bearing the allele in a segregating progeny that was present at a frequency equal to or greater than 0.5 (unpolarized *k*). In the majority of segregation analyses, however, *k* refers to alleles marking the supergene in the *Sb*-linked loci or to specific alleles at the non-supergene loci (polarized *k*).

We tested for significant departures from Mendelian proportions (TRD) at each segregating locus within each progeny by means of one-tailed exact binomial tests (event probability *k* = 0.5; e.g., [7, 10]). These individual tests were followed by a combination bootstrap/subsampling (rarefaction) procedure that allowed us to judge whether the number of significant departures at a locus statistically exceeded the number expected in the absence of TRD (Additional file 2: Text S1). We next conducted a simulation analysis to test whether observed segregation ratios at the four loci with the highest proportions of progenies with significant departures (using binomial tests) were more extreme than expected by chance, given our specific sample sizes (Additional file 2: Text S1). The 97.5th, 95th, 5th, and 2.5th percentiles of 1000 simulated *k* values expected by chance (variation from 1:1 segregation due solely to sampling error) were taken as the limits for statistical significance of the observed values in one- or two-tailed tests.

The frequency and significance of TRD involving the *Sb* supergene in our embryo progenies was evaluated further by considering the three supergene-linked loci (*C294*, *Gp-9*, *i_126*) simultaneously. The expected frequency of departures from Mendelian ratios at *Sb* occurring by chance in the absence of TRD, given our sample sizes, was estimated in a first multilocus simulation analysis that accounted for the observed correlations in segregation ratios between these markers (see below). A second, far more conservative, multilocus simulation analysis that disregarded the correlations between marker segregation ratios also was conducted. See Additional file 2: Text S1 for details of both simulations.

We compared both the proportions of departures from 1:1 segregation ratios and the distributions of *k* values between supergene and non-supergene loci considered as separate classes. In a first set of analyses, we tested whether proportions of significant deviations from 1:1 ratios (determined by binomomial tests) differed between the two classes by conducting a permutation test in which differences in these proportions between paired loci, members of which belonged to the same or to different classes, were compared to differences between paired loci for which class identity of each member was assigned randomly (1000 permutations); assignments were constrained so that the numbers of within- and between-class pairs in the empirical data were preserved in each permutation replicate. We complemented these permutation analyses by conducting Mann-Whitney tests that compared the observed differences in numbers of segregating progenies with *k* ≥ 0.65 between paired markers of the same or alternate classes (see Additional file 2: Text S1). In a second set of analyses, we tested whether distributions of the magnitude of departures from 1:1 ratios (unpolarized *k* values) differed between the two classes. A bootstrap test for differences in mean *k* values was conducted by constructing 5000 bootstrap samples of *k* values for each class of loci using the online program STATKEY [88]; for each bootstrap sample, the mean of non-supergene bootstrapped *k* values was subtracted from the mean for the supergene values and the 95th percentile of the 5000 differences was taken as the one-tailed confidence limit for the null hypothesis that *k* values for the supergene loci did not exceed those for the other loci. Further information on these analyses is given in Additional file 2: Text S1.

Next, a resampling procedure was undertaken to estimate the experiment-wide frequencies of supergene-associated alleles within segregating progenies; this was done to evaluate the overall population-level effect of any allele-specific transmission advantages (Additional file 2: Text S1). We note that because all 101 progenies segregated both *Gp-9* alleles, the results of this resampling procedure yielded unbiased overall population allele frequency estimates for this gene.

The relationship between degree of deviation from Mendelian ratios at supergene-linked markers and extent of failure of eggs to embryonate in a progeny was determined in order to examine the possibility that female gamete killing is the mechanism for segregation distortion (e.g., [5, 10, 89, 90]). For this purpose, we assumed that failure of amplification at all 15 PCR markers indicated that an egg failed to embryonate; such programmed embryo inviability occurs naturally at variable, but generally low, rates among mated polygyne *S. invicta* queens in the USA [75]. Two separate analyses were conducted. First, Spearman correlation coefficients were calculated between the binomial probability of even segregation ratios and the proportion of inviable eggs in a progeny, using the two supergene-linked loci with the largest sample sizes (*Gp-9* and *i_126*). Second, inviability proportions for the pool of 24 progenies shown to have significant supergene TRD by simultaneous consideration of all three supergene loci were compared to the proportions for the remaining 77 progenies using the non-parametric Mann-Whitney Test.

## Results

### Marker and sample characteristics

Each of the 14 microsatellite loci possessed 2–11 alleles (mean = 5.5), with per-locus *H*_exp_ = 0.286–0.819 (mean = 0.617; Additional file 4: Table S2). All polygyne reproductive queens are *Gp-9*^*Bb*^ heterozygotes in invasive populations [43], and *H*_exp_ = 0.5 for this gene in our universally heterozygous study queens.

The numbers of progenies in which embryos were genotyped ranged from 40 for locus *red_ant* to 101 for six of the microsatellite markers and *Gp-9*. Means of > 30 embryos per progeny were successfully genotyped at each microsatellite locus, with a mean of 34.5 embryos scored for *Gp-9* (see Table 1; Additional file 1: Table S1; Additional file 6: Text S2). Heterozygosity for the expected alleles was confirmed for the mother queen at all markers segregating within a progeny; indeed, observed queen genotypes invariably were consistent with those of their embryos for all study progenies. As expected, all 101 progenies segregated both *Gp-9* alleles.

### Progeny characteristics

Based on comparisons of mother queen and offspring embryo genotypes, several different types of progenies were recognized (Additional file 6: Text S2). The great majority of queens (93.1%) mated with a single male, so their progenies comprised simple families. Most such monandrous queens (85.1% of total) mated with a male bearing the *Gp-9*^*B*^ allele (i.e., lacking the *Sb* supergene), a result expected given that most polygyne queens mate with monogyne males (e.g., [91, 92]); a few queens (6.9%) mated with a single *Gp-9*^*b*^-bearing male (i.e., a male with the supergene), whereas a single queen (1.0%) mated with a single diploid male that was heterozygous at *Gp-9*. Among the 6.9% of mother queens that mated multiply, most mated with two males (6.0%) and the remaining one (1.0%) evidently with three males. Two of these polyandrous queens mated exclusively with *Gp-9*^*B*^-bearing males, while the remaining five mated with males of each *Gp-9* haplotype.

The estimated mean pairwise coefficient of relatedness (*r*) between single queens and their mates was 0.041 (see Additional file 6: Text S2). Such low relatedness is expected if polygyne queens mate predominantly with widely dispersing monogyne males [91, 92]. Genetic differentiation between successful reproductives of the two sexes considered as groups also was minimal, with mean *F*_ST_ = − 0.0004 between mother queens and their mates (non-supergene loci). This result accords with the minimal nuclear genetic differentiation reported between geographically adjacent polygyne and monogyne populations in the USA [93], assuming that most of our polygyne queens mated with males from neighboring monogyne colonies. Genetic relatedness of nestmate queens generally was low (mean *r* = 0.069), as reported previously for invasive polygyne *S. invicta* [78, 94], and did not vary significantly among source nests (Additional file 6: Text S2).

### Hardy-Weinberg equilibrium, recombination, and linkage disequilibrium

Genotype frequencies for the 113 study queens matched the frequencies expected under Hardy-Weinberg equilibrium (HWE) at all twelve loci not linked to the supergene (exact tests, 0.145 < *p* < 1.0). In contrast, genotype frequencies at all three supergene loci, *C294*, *Gp-9*, and *i_126*, departed significantly from HWE (all *p* < 0.001), with moderate to large negative estimates of *F*_IS_ indicative of excess heterozygosity (all 113 queens were *Gp-9*^*Bb*^ heterozygotes, yielding *F*_IS_ = − 1.0). Excess heterozygosity is expected at supergene markers because homozygosity for either supergene haplotype effectively is lethal in queens of the polygyne form [43].

**Fig. 1:**
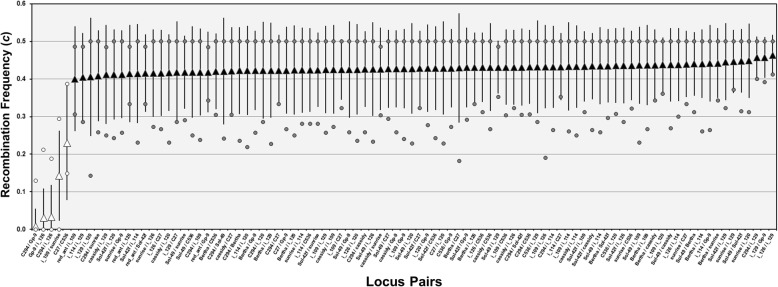
Estimates of recombination frequencies (*c*) between pairs of marker loci. Triangles represent the mean values for jointly segregating embryo (egg) progenies, error bars represent the 95% confidence intervals (CIs), and circles represent the minimum and maximum values. White symbols represent locus pairs with *c* values significantly < 0.5

Estimates of the pedigree recombination frequencies (*c*) between pairs of marker loci are depicted in Fig. 1. The 95% CIs of *c* for only five marker pairs do not overlap with 0.5, the value for freely recombining loci. The three lowest estimates involve all of the pairwise comparisons of the supergene markers (Additional file 6: Text S2; see Additional file 5: Figure S2 for locations of these markers on chromome 16). The two other pairs of loci with *c* significantly < 0.5 are *i_109*/*sunrise* and *C27*/*C536*; the former two loci are 2.4 Mb apart on chromosome 14, while the latter are 5.1 Mb apart on chromosome 6 (Additional file 4: Table S2). These results show that the loci we studied largely segregate independently, as expected given their locations mostly on different chromosomes (ten of the 16 chromosomes represented).

Estimates of the disequilibrium coefficient D* for eggs revealed that only the three supergene marker pairs displayed significant disequilibrium (mean D* = 0.596–0.989 across 100 replicates of single embryos per progeny; mean exact test probabilities of equilibrium, all *p* < 0.001), with the significance of disequilibrium withstanding correction for multiple comparisons for all three pairs (Holm-Bonferroni method, all *p* < 0.015) (also Additional file 6: Text S2). For male mates, eight marker pairs exhibited significant disequilibrium (D* = 0.353–1.0; exact test, all *p* < 0.05), with two of the supergene-linked marker pairs showing the lowest probabilities of equilibrium (D* = 0.284 and 1.0; *p* = 0.002 and *p* < 0.001 for *Gp-9*/*i_126* and *C294*/*Gp-9*, respectively). Only these two pairs retained statistical significance after correction for multiple comparisons (both *p* < 0.025). The minor discrepancies between the results for eggs and males presumably can be attributed to most males having originated from monogyne rather than polygyne mothers. An important point regarding the five *i_126* and seven *C294* alleles we identified is that haplotypes with the *i_126*^*230*^ and, especially, the *C294*^*92*^ alleles almost always also contained the *b* allele of *Gp-9* in both eggs and males (> 95% and > 99%, respectively; Additional file 7: Table S3), demonstrating the utility of the former two alleles as markers for the supergene (*Gp-9*^*b*^ invariably occurs on the supergene in invasive *S. invicta* [43, 77]).

Associations between pedigree recombination frequency (*c*) and gametic disequilibrium (D*) [95] are shown for eggs and males in Additional file 8: Figure S3. For the five locus pairs with mean *c* values significantly < 0.5, gametic disequilibrium was significantly negatively correlated with recombination frequency in both eggs and males (Spearman ρ = − 0.900, *p* = 0.037 for both). For the pairs of freely recombining markers (*c* = 0.5), no association between recombination rate and disequilibrium was observed (eggs: Spearman ρ = − 0.132, *p* = 0.226; males: Spearman ρ = 0.159, *p* = 0.132), as expected given that recombination erodes disequilibrium to yield reduced estimates that vary stochastically (e.g., [95]).

The important finding based on inferred egg haplotypes that some recombination occurs and that disequilibrium is not absolute across the supergene, even for markers located within the same inversion (*C294* and *Gp-9*), is re-enforced by genotypic data from the 113 polygyne mother queens. All of these queens were *Bb* heterozygotes at *Gp-9*, yet seven (6.2%) were apparent homozygotes for the *92* allele of *C294*. Although we cannot rule out the existence of rare null alleles or back mutation at locus *C294*, the observed pattern is consistent with the evidence that supergene recombinants are produced infrequently between the *SB* and *Sb* chromosomes and further suggests that individuals with such haplotypes can survive to become reproductives in the wild. Detectable but typically low levels of recombination between inverted homologous regions, occurring by means of double crossovers or gene conversion, have been widely documented in diverse taxa [96–98].

### Progeny embryo segregation patterns

**Fig. 2:**
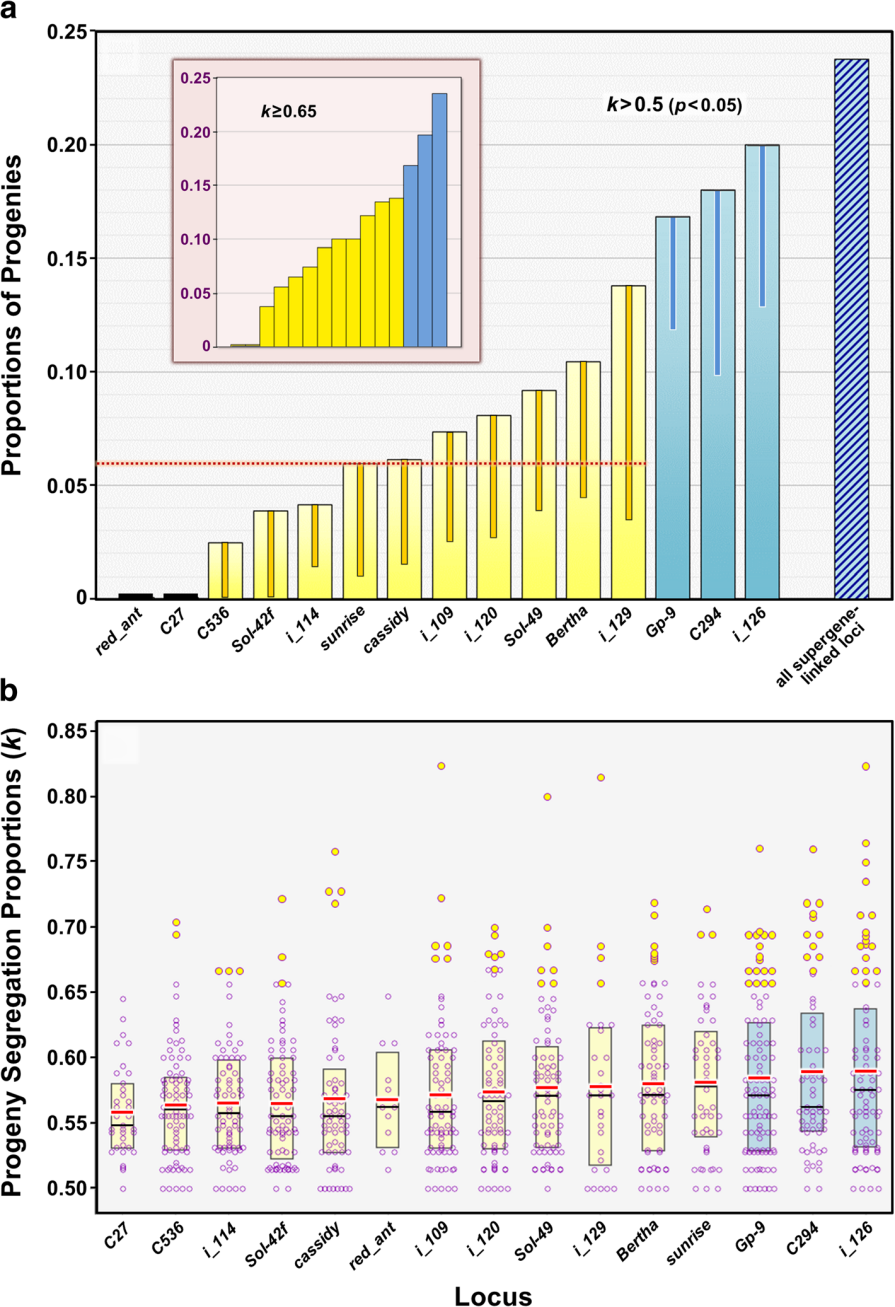
Departures from Mendelian (1:1) segregation ratios in progeny embryos at 15 marker loci. **a** Observed proportions of progenies with significant non-Mendelian ratios (*k* > 0.5, one-tailed exact binomial tests, *p* < 0.05) at each locus. Blue-shaded bars represent the three supergene-associated loci; the hatched bar represents the proportion of departures based on simultaneous consideration of all three supergene-associated loci. The dotted line represents the mean proportion across the remaining twelve (non-supergene) loci (6.0%). Error bars for the observed departures represent the one-tailed lower 95% confidence limits derived from bootstrap/rarefaction analyses (Additional file 6: Text S2); bars that do not extend below 0.05 signify significant TRD for that locus. **Inset**―Observed proportions of progenies with *k* ≥ 0.65 (the threshold above which segregation ratios generally depart significantly from 1:1 with our sample sizes). Loci are arranged in the order: *red_ant*, *C27*, *C536*, *i_114*, *Sol-42f*, *i_109*, *cassidy*, *Sol-49*, *i_129*, *i_120*, *sunrise*, *Bertha*, *Gp-9*, *C294*, and *i_126*; supergene loci are represented by blue-shaded bars. **b** Boxplots depicting segregation proportions (*k*) for each segregating progeny at each locus; *k* values (unpolarized) represent the more common gamete allele in each such progeny in this graph. The boxes depict the interquartile ranges, with black horizontal lines representing the median and red lines the mean for each locus. Individual progenies with significant non-Mendelian ratios (binomial tests, *p* < 0.05) are indicated by the larger yellow circles. Supergene loci are represented by boxes shaded blue; loci in this graph are arranged from low to high mean *k* values

Departures from Mendelian (1:1) segregation ratios, based on binomial tests conducted on each locus segregating in the 101 study progenies, are reported in Table 1 and Fig. 2 (see also Additional file 1: Table S1). The three supergene-linked loci featured the highest proportions of progenies with statistically significant departures, ranging from 16.8 to 20.0% of progenies (Fig. 2a); all three proportions are considerably and significantly in excess of the 5% attributable to Type I errors in the absence of TRD, based on the 95% bootstrap confidence limits (Table 1; Additional file 2: Text S1; Additional file 6: Text S2). The three supergene loci also displayed the highest mean *k* values (measures of magnitude of departure from Mendelian ratios) among the study loci (Fig. 2b). Binomial probabilities of Mendelian ratios were strongly correlated between pairs of supergene loci in each jointly segregating progeny (Additional file 9: Figure S4), as expected given their low recombination frequencies.

Among the remaining twelve, non-supergene, loci, the highest proportion of progenies with significant departures from Mendelian ratios, 13.8%, occurred at *i_129* (Fig. 2a), a marker with relatively few segregating progenies (*N* = 29). Based on the bootstrap confidence limits, the proportions of progenies with significant deviations from 1:1 ratios at this and all other non-supergene loci did not differ from the proportions expected by chance in the absence of TRD (all *p* > 0.05; Table 1). Moreover, the mean proportion of progenies with significant deviations over all twelve of these loci was 6.0%, close to the proportion expected due to Type I errors in the absence of TRD. Among these loci, only the pair *i_109* and *sunrise* exhibited correlated binomial probabilities of Mendelian segregation ratios (Spearman ρ = 0.560; *p* < 0.001 after Bonferroni correction for multiple tests); not surprisingly, this pair also displayed the fourth lowest (and statistically significant) recombination frequency (*c*), just after the three supergene-linked locus pairs (Fig. 1).

Comparison of the supergene and non-supergene loci as distinct classes confirmed that the former displayed both higher proportions of meaningful departures from 1:1 segregation ratios and overall elevated *k* values compared to the latter. The mean difference in proportions of significant departures between pairs of supergene and non-supergene loci (0.123) fell well above the entire range of differences for the 1000 permuted pairs (maximum = 0.093), while the mean difference for paired loci in which both were members of a single class (0.048) fell below the range for the permuted pairs (minimum = 0.063). Mann-Whitney tests on the differences in frequencies of segregating progenies with *k* ≥ 0.65 (indicative of important departures from Mendelian ratios) between paired markers of the same or alternative classes support the preceding inference of relatively more instances of TRD for supergene loci—the median difference for paired loci of the alternative classes (0.123) was significantly higher than that for paired loci of the same supergene status (0.042) (*N* = 36 and 69, respectively, *W* = 2943, *p* < 0.001). Evidence that the supergene loci also displayed elevated *k* values overall compared to the other loci comes from our finding that the mean bootstrap *k* for the former class exceeds that for the latter by 0.015, with the lower one-tailed confidence limit (0.008) greater than zero, the value expected under the null hypothesis that supergene *k* values generally do not exceed *k* values for the other markers. Indeed, only three of the 5000 bootstrap replicate mean differences (0.06%) were less than or equal to zero.

**Fig. 3:**
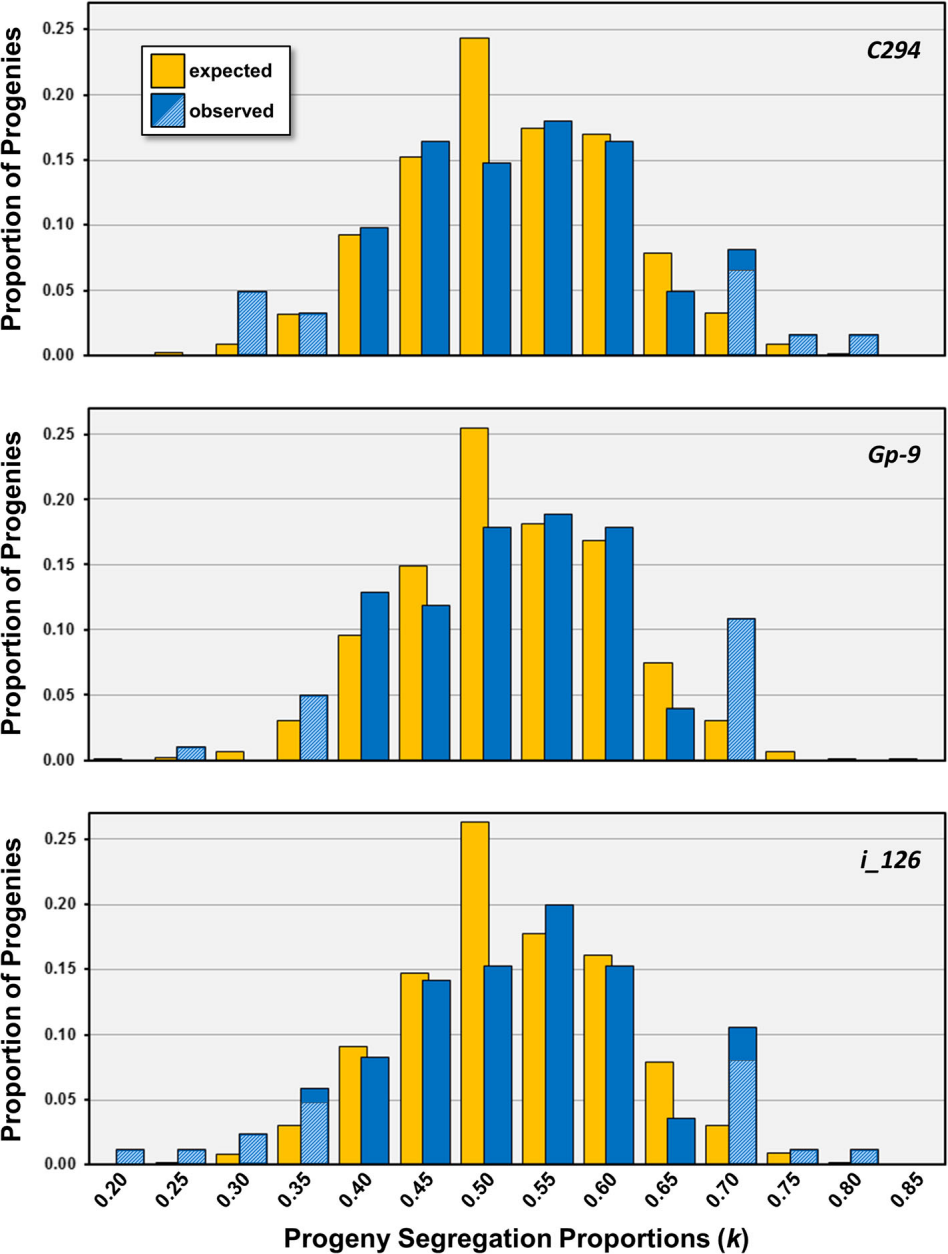
Observed and simulated expected distributions of progeny segregation proportions (*k*) at three supergene-associated loci. The expected values were generated by a simulation model that takes into account specific sample sizes and assumes that departures from Mendelian segregation ratios arise by chance. The segregation proportions (polarized *k*) refer to the supergene-linked alleles *C294*^*92*^, *Gp-9*^*b*^, and, for locus *i_126*, the specific allele on the supergene in each progeny (*i_126*^*230*^ in 60 of 85 [70.6%] segregating progenies). Hatching indicates proportions of progenies with observed *k* values that depart significantly from Mendelian ratios based on the binomial probabilities. Values on the x-axis denote bin maximum values of *k*

Results of a simulation analysis for comparing the distributions of observed segregation proportions (*k*) at the three supergene loci with those expected by chance, given our specific sample sizes, are shown in Fig. 3. Distributions for all three loci show excesses of extreme *k* values compared to those expected. Specifically, values of *k* for ten progenies segregating at *C294* fell within the lower and upper 2.5% tails for the simulated progenies and thus are judged to deviate significantly from Mendelian ratios (3.05 total are expected by chance), thirteen progenies segregating at *Gp-9* fell within the tails (5.05 expected), and 20 progenies segregating at *i_126* fell within the tails (4.25 expected). The results of this simulation thus suggest that significant deviations from Mendelian ratios occur at each supergene marker in three to five times more progenies than expected in the absence of TRD. For the non-supergene locus *i_129*, three segregating progenies fell within the 5% tail, which does not differ significantly from the fewer than two expected by chance (Fisher exact test; *p* > 0.61).

An unexpected feature of the Mendelian deviations for the supergene loci evident from the above is that not all departures feature a surplus of the allele associated with the *Sb* supergene (and with a hypothesized drive locus) (Fig. 3). Indeed, such cases are not much more common than those in which the alternate, non-supergene-linked, allele predominates (termed “reversal-of-drive” or “negative distortion”; [13, 17, 27, 99–103]), resulting in overdispersed *k* values for these three markers. Based on the binomial tests, six progenies displayed significant excesses and five displayed deficiencies for the *C294*^*92*^ supergene allele (1.5 of each expected by chance), eleven progenies displayed excesses and six deficiencies for the *Gp-9*^*b*^ supergene allele (2.5 of each expected by chance), and nine progenies displayed excesses and eight deficiencies for the *i_126* supergene alleles (2.2 of each expected by chance). Thus, drive reversal at the homologous region of the *SB* chromosome appears to be as potent as the predicted drive associated with the *Sb* supergene.

**Fig. 4:**
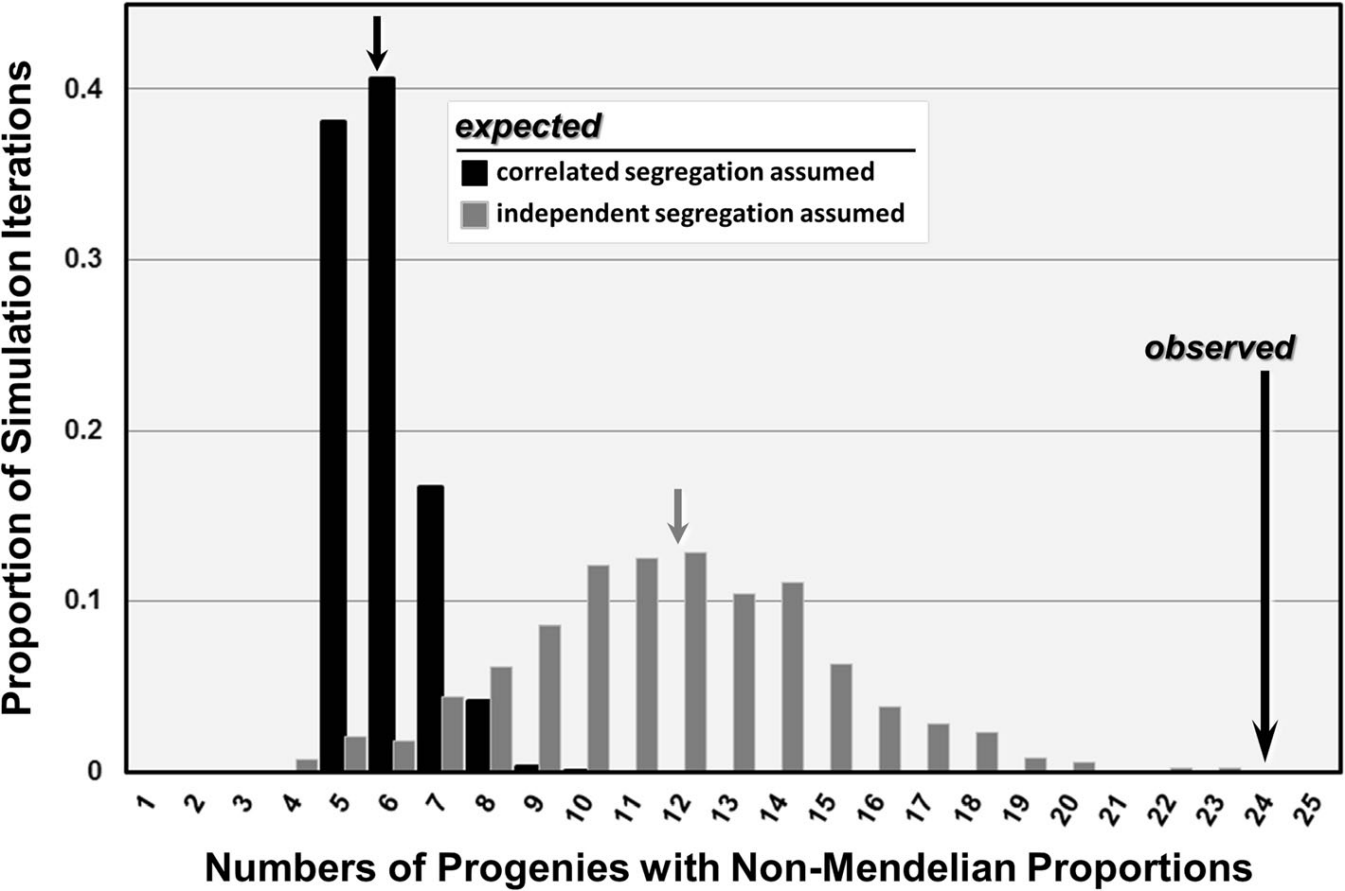
Observed and simulated expected distributions of progenies with non-Mendelian segregation proportions of the *Sb* supergene. The distributions of expected numbers were generated by simulation models that simultaneously considered all three supergene-linked loci and accounted for specific sample sizes. The models assume that departures from Mendelian (1:1) segregation ratios arise solely by chance, with one model incorporating correlations between marker segregation ratios (black bars), and the other, more conservative model disregarding this non-independence (gray bars). Small arrows show the mean numbers of expected progenies from 1000 iterations of each model, whereas the large arrow shows the observed proportion of non-Mendelian progenies

Considering all three supergene loci simultaneously, 24 of the 101 progenies (23.8%) exhibited significant distortion at one or more loci (binomial tests). The proportions of progenies expected to exhibit such departures in the absence of drive, obtained via multilocus simulations explicitly incorporating our sample sizes, are shown along with the observed proportion in Fig. 4. For the model incorporating segregation ratios correlated between loci, the simulated numbers of progenies that depart significantly from Mendelian ratios ranged from five to ten across the iterations, with a mean of 5.9. This is fewer than one-quarter of the number actually observed to display significant departures at one or more supergene loci. For the more conservative model that ignored between-marker correlations, numbers of progenies deviating significantly from Mendelian ratios ranged from three to 23, with a mean of 11.8 progenies, half the number observed. The 95% CIs (one-tailed) for the latter model results do not include values greater than 17 progenies. These results further substantiate the conclusion that relatively modest but real segregation distortion exists at the supergene region. Importantly, for all 17 progenies in which two or more of the supergene loci exhibited significant Mendelian departures featuring supergene alleles, these departures involved congruent excesses or deficiencies of the alleles across the loci reflective of drive or drive reversal (nine of the former and eight of the latter; see Additional file 1: Table S1); this re-enforces the conclusion that drive reversal is a real feature of the *Sb* supergene drive system.

A resampling procedure undertaken to estimate the overall (population) frequencies of supergene alleles within segregating progenies showed that alleles associated with the supergene did not gain a consistent transmission advantage over their alternate alleles. Specifically, these frequencies were very close to 0.5 at all three supergene loci: 0.497, 0.501, and 0.502 for *C294*, *Gp-9*, and *i_126*, respectively (mean binomial probabilities of equal allele frequencies across the population of segregating progenies, *p* = 0.299, 0.277, and 0.289, respectively). Thus, the effects of *Sb* drive and reversal-of-drive on supergene frequency appear to be relatively balanced in our study population.

Examination of the distributions of *k* values for individual alleles at each of the twelve non-supergene loci revealed no evidence of consistent, directional segregation distortion at any of these loci, with the middle quartiles of *k* values for all adequately sampled alleles overlapping 0.5 (Additional file 10: Figure S5). These results, in agreement with the overall low proportions of segregating progenies with significant non-Mendelian ratios, support the conclusion that TRD is not important at the non-supergene loci.

Queens producing progenies that exhibited significant drive (or drive reversal) at the three supergene markers originated from ten of the twelve polygyne source colonies. The frequency of such queens varied from 11.1% (1/9) to 50.0% (2/4) per colony in the ten with any, and variation in this frequency across all twelve colonies was not significant (χ^2^ = 14.5, dF = 11, *p* = 0.220). Indeed, median *k* values for *Sb*-linked alleles for all nestmate queens (not just those with significant TRD) did not differ significantly according to queen colony of origin (Kruskal-Wallis test; *H* = 9.0, 17.2, and 14.2 for *C294*, *Gp-9*, and *i_126*, respectively; all *p* > 0.10). Thus, there is no evident colony-level effect on the tendency of queens to produce eggs deviating from Mendelian ratios of *Sb*.

Three of the 24 queens producing non-Mendelian supergene ratios were polyandrous rather than monandrous (12.5%), not significantly different from the proportion of such queens producing Mendelian ratios (5.2%; Fisher exact test, *p* = 0.352). Further, queens producing non-Mendelian ratios were no more closely related to their mates than were other queens (mean *r* = 0.061 and 0.037, respectively; Mann-Whitney test, *W* = 1449, *p* = 0.073). With respect to the supergene status of their mates, monandrous queens producing non-Mendelian supergene ratios mated with *Sb* or *SB* males at frequencies similar to queens producing Mendelian ratios (Fisher exact test, *p* = 0.661), a result not affected by polarity of *k* in the progenies (i.e., drive vs. drive reversal; Additional file 11: Fig. S6). Thus, queens producing progenies with significant supergene TRD appear not to be remarkable in terms of their mating habits.

Associations between genetic relatedness (*r*) for pairs of nestmate queens and congruence in their progeny’s degree of deviation from 1:1 supergene segregation ratios (∆*k*) were examined to learn if relatedness is predictive of similarities in such deviations. No association was detected (Additional file 6: Text S2), as expected if there is not a strong heritable basis for extent of deviations from Mendelian ratios at the supergene. Importantly, queens with significant supergene TRD did not stand out with respect to their genetic relatedness to other nestmate queens also displaying significant TRD; that is, pairs of such queens generally were neither more nor less closely related to one another than were queens in other pairs, regardless of whether the polarity of focal queen TRD was in the same direction (e.g., both displayed drive) or differed between them (one displayed drive and the other drive reversal) (Kruskal-Wallis test, *H* = 0.87, *p* = 0.65; also Additional file 6: Text S2; Additional file 12: Figure S7). These findings are discussed more fully with respect to a proposed mechanism of drive reversal in Additional file 13: Text S3.

Several explicit analyses confirm what is perceptible in some of the previous results―queens whose progenies exhibited significant distortion at the supergene loci were not prone to produce eggs that departed from Mendelian ratios at other loci (see Additional file 6: Text S2). Specifically, (i) the binomial probabilities of Mendelian ratios at the supergene loci in a progeny were not associated with the probabilities for the remaining segregating loci in that progeny, (ii) the 24 progenies implicated as displaying supergene-associated distortion did not, as a group, display a greater tendency for significant non-Mendelian ratios at the remaining segregating loci compared to the other 77 progenies, and (iii) a positive relationship was found between the mean binomial probability of Mendelian ratios across segregating loci in a progeny and the coefficient of variation (CV) for these single-locus probabilities, implying that queens with higher overall levels of non-Mendelian ratios also displayed higher variation across the loci in these levels.

Queenless groups of 2–3 workers succeeded in maintaining apparently viable eggs/embryos at very high proportions in the set of tests supplementing the TRD analyses. A total of 1598 of 1637 viable embryos (98%) was recovered intact from the 40 test units after 48 h, with only one unit maintaining fewer than 88% and most units (23) maintaining all of the embryos provided to them (see Additional file 14: Figure S8). This result suggests that such small groups of workers are fully competent to maintain viable eggs/embryos under the conditions we used to measure TRD. It also suggests that worker culling of embryos based on embryo supergene status is highly unlikely to explain the TRD we observed, as this would imply the loss of 20–35% of embryos in about one-quarter of the supplementary test units (based on *k* values for the 24 progenies with significant TRD in the main experiment; see Additional file 6: Text S2). More generally, the predicted pattern of loss of embryos due to worker intervention (*M*_TRD_; proportionate worker-induced selective mortality of embryos) required to explain observed *k* values in our TRD study differs strikingly from the pattern actually measured in the supplementary test units (Additional file 14: Figure S8).

Finally, association of the level of segregation distortion at the supergene loci with the extent of egg embryonation failure in a progeny was examined to investigate if female gamete dysfunction may underlie such distortion (e.g., [5, 10, 89, 90]). Proportions of non-embryonated eggs in the 101 progenies ranged from zero to 0.278, with a mean of 0.030, very similar to the mean proportion reported decades earlier for laboratory-maintained mated polygyne *S. invicta* queens from the same area (0.040; [75]). Based on the two supergene loci with the largest sample sizes, no significant relationship was observed between the extent of distortion (assessed by the binomial probability of even segregation) and the proportion of non-embryonated eggs (*Gp-9*: *N* = 101, Spearman ρ = 0.014, *p* = 0.892; *i_126*: *N* = 85, Spearman ρ = 0.036, *p* = 0.744). Moreover, comparison of non-embryonation proportions for the 24 progenies implicated as having supergene-associated TRD with the proportions for the remaining 77 progenies revealed no significant difference between the groups (Mann-Whitney Test [one-tailed], *W* = 1231, *p* = 0.478). Given that female gamete mortality may be expected to lead to a positive association between level of distortion and frequency of non-embryonation, these analyses offer no support to the hypothesis that this mechanism underlies significant supergene TRD in polygyne *S. invicta*.

## Discussion

### Patterns of transmission ratio distortion (TRD)

The *Social b* (*Sb*) supergene on chromosome 16 (the “social chromosome”) in the fire ant *S. invicta* plays a major role in determining the form of social organization, most importantly, whether colonies contain a single or multiple reproductive queens. Remarkably, other features of the supergene suggest that it is a selfish genetic element and raise the possibility that it may act as a transmission ratio distorter [49, 57]: the *Sb* supergene contains multiple inversions and is pericentric, so that recombination consequently is greatly reduced; the centromere of chromosome 16 is exceptionally large, possibly reflecting a role in chromosomal competition for inclusion in the egg during meiosis; the supergene contains deleterious mutations and abundant transposable elements; and the supergene mediates two known selfish features—“green-beard” behavior and a bias in the development of diploid larvae toward the queen rather than worker caste [43, 47, 59, 65, 67]. Together, these features led to our prediction that meiotic drive or some other form of early TRD favors *Sb* supergene transmission at the expense of its homolog, thus aiding its persistence in populations despite its negative fitness effects on individual fire ant sexuals.

Our survey of segregation ratios in embryo progenies of 101 polygyne queens using three supergene-linked markers and twelve additional markers located across the genome revealed that significant departures from Mendelian ratios occurred at the supergene in 3–5 times more progenies than expected in the absence of TRD. By comparison, the non-supergene loci displayed lower proportions of progenies with significant departures, with the mean proportion close to that expected in the absence of TRD with Type I error. Additionally, the magnitude of departures from 1:1 ratios (*k* values) averaged higher for alleles at all three supergene loci than for alleles at any of the non-supergene loci. These results demonstrate the existence of supergene-associated TRD at modest yet significant levels, and further reveal substantial inter-progeny variation in the segregation ratios. This latter finding is important because such variation rarely has been studied in wild populations [5, 7, 10, 22, 31, 35], yet quantifying it is key to understanding the genomic architecture of drive elements and their antagonists (see below; also [5, 7, 12, 17, 33]).

A surprising feature of the large inter-progeny variation in embryo TRD we observed is that significant departures from Mendelian ratios did not always favor alleles in the *Sb* supergene (and a hypothesized drive locus also contained therein), but often instead favored the alternate, non-supergene-linked allele(s) on the homologous *SB* chromosome. Such drive reversal [13, 17, 27, 100–103] implies a complex multilocus genetic architecture that is forged by intragenomic conflict and may feature multiple genetic modifiers (including suppressors) of social chromosome drive [7, 12, 17, 22]. Although the details of this architecture, including the nature, form of epistatic interactions, and locations of modifiers throughout the genome, remain to be revealed, the genetic background of individual polygyne (*SB*/*Sb*) queens evidently affects the expression of supergene-associated drive. As might be expected given the relatively common occurrence of drive reversal in our system, supergene haplotypes evidently gain no consistent transmission advantage over their alternate haplotypes at the population level. From this we conclude that *Sb*-linked drive and *SB*-linked reversal-of-drive appear to be at a balanced equilibrium in invasive polygyne *S. invicta* (see [12, 24, 30]).

Significant TRD was recorded at all three supergene-linked loci we surveyed, with the probabilities of distortion highly correlated across the markers. This pattern is predicted based on the physical locations of the markers on, and structural features of, chromosome 16 (Additional file 5: Figure S2). The gene *Gp-9* and microsatellite marker *C294* are located near one another within the largest of three inversions that collectively have been assumed to demarcate the supergene boundaries. Microsatellite *i_126* is located outside any known inversions on the opposite arm of the chromosome near the centromere, more than a third of the length of the chromosome from the two other markers. The patterns of recombination and gametic disequilibrium we observed, with the adjacent marker pair displaying very low recombination rates and high disequilibrium and the other pairs displaying slightly higher recombination and lower disequilibrium, are fully congruent with this structural information. The presence of several adjacent or overlapping inversions, the proximity of one of them to the centromere (where crossing over evidently is strongly reduced [104], see also [12, 27, 57]), and the accumulation of repetitive elements (transposons) across the region [49, 60] likely all combine to strongly limit rates of recombination. Thus, although the exact boundaries of the supergene remain somewhat unclear, and may vary among individuals and populations, they likely span a region of chromosome 16 well beyond the known inversion breakpoints, a conclusion substantiated by mapping data showing constant genetic map distances that extend well past marker *i_126* proximally on the *Sb* chromosome ([47], Y. Zheng et al., unpublished; see Additional file 5: Figure S2). Sizeable zones in which recombination is suppressed often flank inversions, although the causes of this suppression remain obscure [e.g., [105–107]].

The *SB*/*Sb* recombination and incomplete gametic disequilibrium we observed across the supergene, especially between markers *C294* and *Gp-9*, are significant because they suggest that selection may not preserve a single uniform supergene haplotype in the service of regulating polygyny in *S. invicta*. This conclusion is re-enforced by our discovery that some reproductive queens of this form were *Bb* heterozygotes at *Gp-9* but apparent homozygotes for the *92* allele of *C294*, evidence for viability of *SB*/*Sb* recombinants, and is supported further by observed patterns of sequence variation across the supergene and its homologous region that are as expected following some historical recombination ([48], see also [98]). The significant excess heterozygosity we observed in polygyne reproductive queens at all three supergene-linked markers can be explained by the fact that all such queens are heterozygotes for the supergene and *SB* homologous region; in accord with the recombination data, all are *Bb* heterozygotes at *Gp-9*, the great majority (93.8%) are heterozygotes for the *92* allele of *C294*, and an excess number are heterozygotes for the *230* allele of *i_126* (60.2% observed vs. 42.1% expected under Hardy-Weinberg equilibrium).

### Stage and mode of transmission distortion

Clarification of the specific mechanism of supergene-associated TRD in *S. invicta* awaits detailed investigation, but results of the present study offer some clues regarding the stage, and hence mode, of distortion. Embryos were genotyped as soon as logistically feasible after oviposition (48-60 h) in order to minimize the possibility that differential mortality during embryogenesis, rather than events surrounding oogenesis, were responsible for any distortion. Nonetheless, there existed a short window during which endogenous or worker-mediated embryo mortality, or other factors, could have altered transmission ratios. One explanation for such TRD, differential fertilization success/zygote viability due to egg-sperm compatibility issues associated with an evolutionary arms race involving male gametes’ access to eggs (e.g., [8, 9, 23, 108]), is not supported by our data; we found instances of both significant supergene drive and drive reversal for progenies sired by single *SB* males (the great majority of progenies in our study), in direct contradiction to predictions of this hypothesis (see also below).

Another explanation, differential mortality of embryo supergene homozygotes and heterozygotes, may be posited in light of the fact that homozygosity for either haplotype is effectively lethal in adult polygyne queens [43]. However, such an explanation is negated by the distributions of *k* values for progenies fathered by single males bearing or lacking the supergene (progenies containing heterozygotes along with either *Sb* or *SB* homozygotes, respectively). Specifically, the equal numbers of significant deviations favoring or disfavoring supergene-bearing eggs in progenies fathered by *SB* males (eleven of each), as well as the similarity in distributions of *k* for progenies fathered by either *SB* or *Sb* males, speaks against any role for diminished supergene homozygote (or heterozygote) embryo viability in explaining the TRD we observed (Additional file 11: Figure S6).

Intervention by the workers tending embryos in our isolation units, via cannibalism or neglect, could conceivably have played a role in TRD, behavior that might be viewed as a form of competition between embryos with and without the supergene for maternal (alloparental) care and resources (e.g., [9]). Such TRD mediated through the extended phenotype of the mother queen (her sterile adult worker offspring), were it to occur, is as relevant as, and perhaps more intriguing in an ultimate sense than, true meiotic drive. Nonetheless, the very low number of workers (2–3) in each TRD progeny unit makes it unlikely that such complex social behavior involving discrimination among embryos on the basis of their genotypes could be displayed, given that similar behaviors normally are the emergent outcomes of collective decisions of thousands of workers in colonies of this highly eusocial species (e.g., [67]). This conclusion is re-enforced by the results of our supplementary rearing trials, which showed that queenless groups of just a few workers maintained apparently viable for 48 h virtually all eggs/embryos they were provided (Additional file 14: Figure S8). This pattern stands in strong contrast to the expectation of 20–35% embryo mortality (*M*_TRD_) in about one-quarter of these test progenies were selective worker cannnibalism the primary cause of the TRD observed in our main experiment.

Several considerations are consistent with TRD in polygyne *S. invicta* occurring before fertilization and thus constituting segregation distortion, and the possibility exists that it may in fact be “true” meiotic drive. True meiotic drive takes advantage of the arena for competition provided by intrinsic asymmetries in meiotic cell fate during oogenesis [19]; thus, it is not surprising that this form of TRD is restricted to females in most plants and animals [7]. In contrast, post-meiotic (pre-fertilization) segregation distortion, which typically acts via breakdown of gamete viability or functionality, normally is restricted to males [8]. Aside from the unlikely prospect of fire ant queens sustaining substantial fecundity loss, the lack of an association between proportion of non-embryonated eggs and extent of segregation distortion in our study also mitigates against such “gamete killing” as the responsible form of segregation distortion. Moreover, the unusually large centromeres of *S. invicta* (averaging 34% of chromosome length) suggest a history of competition between homologs for preferential access to the meiotic spindle assembly and for segregation into an oocyte (“centromere drive”; [64, 109]), and there exists some variation in centromere size between the *Sb* and *SB* chromosomes (Y. Zheng et al., unpublished). Indeed, because of their roles as sites of attachment to the microtubule lattice, variant centromeres and heterochromatic neocentromeric elements are viewed as definitive genomic substrates for mediating non-Mendelian meiotic segregation [20, 38, 58, 60, 110, 111]. Notably, the behavior of such variants can be modified by enhancer or suppressor genes involved in production of centromeric repeat sequences or of the kinetochore protein complex, which links centromeric chromatin and spindle microtubules to coordinate chromosomal movement, resulting in the evolution of complex genetic architectures responsible for both production of meiotic drive and restoration of fair segregation [110, 112, 113]. Although the causal relationships between centromere drive and supergene segregation distortion in fire ants, if any, remain to be clarified, the fact that dramatic centromere expansion occurred at the base of the clade comprising South American fire ant species with supergene control of social organization (including *S. invicta*) [64] suggests that the phenomena are closely tied.

Finally, we note that biased gene conversion during meiosis is an unlikely explanation for the observed TRD in polygyne *S. invicta* queens. This is because parallel transmission biases were detected at pairs of supergene markers separated by as much as 10 Mb, whereas biased gene conversion is expected to act locally [10].

### Genetic mechanism of drive reversal

A hallmark of true meiotic drive and other TRD systems is the presence of intragenomic conflict related to the transmission advantage of drive elements coupled with the negative organismal fitness effects associated with possession of these elements. This conflict reflects the fact that drive homologs experience a direct transmission disadvantage, whereas unlinked genes elsewhere in the genome suffer adverse consequences by being transmitted to suboptimally adapted individuals while not benefitting from a transmission advantage [16, 24]. As a result, suppressors of drive in homologous chromosomal regions and other, unlinked genomic locations are expected to evolve, as are enhancers of drive in linked regions, leading to continuing antagonistic coevolution between genes that cause or enhance drive and those that suppress it [7, 29, 36, 114, 115]. Selection pressure on suppressors is expected to be most intense in the region of the homologous chromosome corresponding to the drive complex [12, 27], and our queen relatedness data are consistent with such a location in the fire ant genome (Additional file 13: Text S3).

Drive reversal can be viewed as an extreme manifestation of individual variation in the strength of drive in the sense that both likely depend on the genetic background, that is, on the genome-wide presence and nature of multiple segregating suppressors and enhancers and their epistatic interactions [17, 22, 36]. Thus, the variation we observed in both the magnitude and direction of drive in polygyne *S. invicta* queens may be linked to the presence of variants segregating at such modifier loci. One relatively simple scenario for the genetic mechanism posits multiple unlinked suppressor loci that segregate allelic variants with additive effects to counter *Sb* drive (see Additional file 13: Text S3; Additional file 15: Figure S9). While speculative, such suppressors of true meiotic drive, as well as any enhancers in the supergene, may act (i) by regulating duplication/expansion of centromeric DNA repeat domains and/or levels of kinetochore complex proteins associated with the social chromosome centromere [7, 38, 111], or (ii) by influencing the development of meiotic spindle asymmetry that facilitates orientation of selfish centromeres towards the egg pole [111].

Drive reversal has been shown to be generated, at least in part, by recombinant driver loci for the *t*-complex of mice [13] and the *SD* complex of *Drosophila melanogaster* [12], illustrating the importance of complete disequilibrium across the critical components of a selfish element to its full functionality. In our study, all relevant progenies displaying significant supergene drive or drive reversal had congruent deficiencies or excesses of supergene-linked alleles across our three markers, which span much of the supergene. This suggests that historical single crossovers between *Sb* and *SB* haplotypes within the interval separating our markers are not responsible for drive reversal. Double crossovers involving recombination of the entire supergene drive complex (locus) into the *SB* haplotype background, which could preserve allelic congruence across our markers, also are unlikely to explain drive reversal, based on the general rarity of this form of recombination between large inverted and non-inverted segments [106] and the likely instability of the presumably large *Sb* drive complex in a haplotype (*SB*) lacking inversions (see Additional file 13: Text S3; also Additional file 16: Figure S10). Whatever the mechanism of drive reversal in fire ants, similar phenomena may prove to be more common than currently appreciated once appropriate surveys of other wild populations are undertaken, and their molecular dissection promises to yield useful general insights into TRD.

## Conclusions

The *Sb* supergene determines the form of colony social organization in *S. invicta*, and several features of this selfish element predicted that it may also act to favor its own transmission during reproduction. Our survey of a large number of embryo progenies using supergene-linked and other markers revealed departures from Mendelian ratios of the supergene at frequencies 3–5 times higher than expected in the absence of transmission ratio distortion (TRD) and than found at non-supergene loci. Unexpectedly, significant embryo TRD involved not only excesses of alleles associated with the *Sb* supergene (and with a hypothesized drive element contained within it), but also recurrent excesses of the alternate alleles on the homologous *SB* chromosome. The common occurrence of such drive reversal in this system is consistent with the evolution of a complex genomic architecture featuring multiple suppressors of supergene drive, such that the *Sb* haplotype evidently gains no consistent transmission advantage over its homolog at the population level. Evidence points to “true” meiotic drive as the most likely mechanism for the TRD patterns we observed. These findings are important because they reveal the presence and distribution of TRD, a factor mediating diverse evolutionary phenomena, in a wild population in which it was predicted, and they suggest its proximate causes.

We further observed low levels of recombination and incomplete gametic disequilibrium across the supergene, even between adjacent markers within the same inversion. Discovery of recombinant supergenes bearing traces of *SB* ancestry in the native range would confirm historical recombination and demonstrate that selection does not preserve a single uniform supergene haplotype for regulating a derived form of fire ant social organization.

## Additional files

Additional file 1: **Table S1.** Polygyne *Solenopsis invicta* nest information, mother queen characteristics, and numbers of embryos genotyped in progenies. (PDF 200 kb)

Additional file 2: **Text S1.** Methods―additional information. (PDF 155 kb)

Additional file 3: **Figure S1.** Protocol for collecting progeny embryos for *Sb* supergene transmission ratio distortion (TRD) study in *Solenopsis invicta*. Families (progenies) of diploid embryos were obtained from individual mother queens initially isolated in plaster-bottomed petri dishes for three to four weeks with several thousand adult workers (colony fragments) **(i)**. Queens confirmed to be mated (producing worker brood) at the end of this period were then isolated with 2–3 workers in plastic plaster-bottomed specimen cups (isolation cups) **(ii)**. Each such queen was removed from the cup after 12 h then frozen in a − 80 °C freezer **(iii)**. Eggs laid by the queen were maintained in the cup with the workers for an additional 48 h (by which time they were embryos within the egg coat), then transferred into a gelatin capsule and placed immediately in a − 80 °C freezer pending DNA extraction **(iv)**. Thirty-six haphazardly selected embryos per progeny were sampled for genotyping at *Gp-9* and 14 microsatellite loci **(v)**. (TIF 1353 kb)

Additional file 4: **Table S2.** Characteristics of marker loci genotyped in polygyne queens and their progeny embryos (male genotypes inferred). (PDF 10 kb)

Additional file 5: **Figure S2.** The social chromosome (chromosome 16, *Sb* variant) of polygyne *Solenopsis invicta*. Depiction is based on reference genome build *Si_gnH_C3* of a haploid *SB* male from the USA [51]. Locations of three inversions on the distal arm of the *Sb* chromosome (two of which overlap) are shown by blue bars, and the positions of three supergene-linked marker loci (*i_126*, *C294*, *Gp-9*) and the centromere are indicated (physical distances between loci are shown with parentheses; size of the centromere is not shown to scale). The region of reduced recombination on *Sb* chromosome 16, estimated by mapping to the new reference build 2796 RADseq SNPs from 92 haploid sons of a heterozygous *SB*/*Sb* queen [47], is indicated by the orange bar. Purple and grey blocks represent Pacific Biosciences (PacBio) contigs of the raw assembly. (Based on original figure by Y. Zheng.) (TIF 221 kb)

Additional file 6: **Text S2.** Results―additional information. (PDF 144 kb)

Additional file 7: **Table S3.** Proportions of haplotypes with a supergene-associated allele that also bear such an allele at another supergene locus. (PDF 97.8 kb)

Additional file 8: **Figure S3.** Associations between recombination frequency (*c*) and gametic disequilibrium (D*) in progeny embryos (eggs) and males. The five locus pairs with values of *c* significantly less than 0.5 are indicated by the larger circles identified in the legend (the top three listed pairs are supergene loci). Locus pairs involving *red_ant* are excluded because of small sample sizes. (TIF 363 kb)

Additional file 9: **Figure S4.** Associations of binomial probabilities of Mendelian segregation ratios between supergene loci. Colored lines represent the least squares regression lines fitted to the three sets of values. Samples sizes, Spearman ρ and associated probability values (after Bonferroni correction for multiple tests), and *R*^2^ (coefficient of determination) values are shown in the inset. (TIF 669 kb)

Additional file 10: **Figure S5.** Distributions of segregation proportions (*k*) in embryo progenies for 56 alleles of twelve non-supergene-linked microsatellite loci. Boxes represent interquartile ranges, whereas whiskers indicate the range limits. Within boxes, black horizontal bars represent the medians and red dots the means. Five alleles at four of the loci that segregated in only a single progeny are not included. The three alleles with boxes not overlapping 0.5 occurred in very few segregating progenies (numbers shown in blue). As expected for alleles that segregate in Mendelian ratios, estimates of the mean deviations from 1:1 ratios (│0.5 - *k*│) tend to decrease with larger numbers of segregating progenies studied (smaller plot). (TIF 1481 kb)

Additional file 11: **Figure S6.** Segregation proportions (*k*) in polygyne queen embryo progenies fathered by males lacking or bearing the *Sb* supergene. Values that differ significantly from 1:1 segregation ratios at one or more of the three supergene loci (binomial tests) are indicated by the yellow dots. The distributions of progeny *k* values, which represent weighted means across the supergene loci for each progeny, do not differ significantly with respect to the supergene-related haplotype of the fathers (Mann-Whitney test, *N* = 86 and 7, *W* = 3950, two-tailed *p* = 0.185). (TIF 157 kb)

Additional file 12: **Figure S7.** Genetic relatedness between pairs of nestmate queens (*r*) in relation to their patterns of supergene TRD. Queen pairs are classified into three types: pairs in which one or both queens did not display significant TRD, pairs in which both queens displayed TRD of the same polarity (either both displayed drive or both displayed drive reversal), and pairs in which the queens displayed TRD of opposite polarity (one displayed drive and the other drive reversal) (*N* = 359, 12, and 11, respectively). (A) Distributions of *r* values for queens of the different types; sizes of the symbols indicate the relative numbers of identical *r* values (smallest symbols of each type, *N* = 1). Similar results showing that queens with significant TRD do not display unusually high or low relatedness to nestmate queens with TRD were observed as well for the seven individual source colonies from which these queens originated. (B) Box and whisker plots of the data. The boxes represent the interquartile ranges while the whiskers represent the 90th percentiles. Medians for each type are zero; means are indicated by the red dotted lines. Pairs of queens in which both members displayed TRD were neither significantly more nor less closely related to one another than were pairs of nestmate queens not displaying TRD (see main text). (TIF 631 kb)

Additional file 13: **Text S3.** Genetic mechanism of drive reversal―additional information. (PDF 135 kb)

Additional file 14: **Figure S8.** Numbers of eggs/embryos successfully maintained by 2–3 polygyne workers over a 48 h period in supplementary tests. Dark red portions of the bars indicate the differences between initial and final numbers of intact eggs/embryos present in each test unit, corresponding to *M*_TRD_ values if the losses are due to worker cannibalism (*M*_TRD_ is defined as the proportionate worker-induced selective mortality of embryos that yields biased segregation ratios). The smaller plot contains boxplots comparing distributions of presumptive *M*_TRD_ values for our 101 TRD study progenies and 40 supplementary test units. *M*_TRD_ for the former was estimated from the unpolarized *k* values (see Additional file 6: Text S2 for formula), while for the latter it was directly equated with observed embryo losses. Boxes represent the interquartile ranges, while whiskers represent the 5th and 95th percentiles. Means of each distribution are indicated by the red dotted lines. Colored lines next to each box depict values for three summary measures of statistical dispersion in *M*_TRD_ values for each group (collectively termed Average Absolute Deviation statistics); these are the Mean Absolute Deviation from the Mean (red), Mean Absolute Deviation from the Median (gold), and Median Absolute Deviation from the Median (green). The boxplots and dispersion statistics show that *M*_TRD_ values in our supplementary test units are too low and unvarying to support the hypothesis that worker intervention during embryogenesis rather than segregation distortion (meiotic drive) primarily caused significant supergene TRD in our main experiment. (TIF 1564 kb)

Additional file 15: **Figure S9.** Hypothetical scenario for additive genetic mechanism of *Sb* supergene drive reversal in polygyne *Solenopsis invicta*. The presence of seven suppressor loci segregating wild-type alleles (ineffective in suppressing *Sb* drive) along with suppressor alleles is depicted as an example of such a multilocus system, with several different multilocus haplotypes arising from recombination and segregation in the population illustrated. The inset depicts the distribution of multilocus haplotypes with varying proportions of suppressor alleles expected in an equilibrium population of 5000 haplotypes with the seven suppressor loci in gametic equilibrium, equal allele frequencies, and no selection. Haplotypes with no or very few suppressor alleles allow *Sb* drive to prevail, those with high or maximal numbers of suppressor alleles overwhelm *Sb* drive to cause drive reversal, and those with intermediate proportions of suppressor alleles (the most common circumstance―inset) neutralize *Sb* drive to favor Mendelian segregation ratios. The suppressor loci are shown in the region of *SB* chromosome 16 homologous to the supergene (see Additional file 13: Text S3 for rationale). Centromere is not shown to scale (see Additional file 5: Figure S2). (TIF 781 kb)

Additional file 16: **Figure S10.** Hypothetical scenario for a historical double crossover involving the putative supergene drive locus as the genetic mechanism responsible for *Sb* supergene drive reversal in polygyne *Solenopsis invicta*. (A) Double crossover between the wild-type *Sb* and *SB* social chromosomes in the segment between markers *i_126* and *Gp-9* transferred the complete drive complex (locus) from *Sb* to *SB* without altering the congruence of alleles at all three supergene markers observed in progenies with both drive and reversal-of-drive. Two novel recombination products were generated, a *SB* chromosome containing the drive locus and a *Sb* chromosome without it, and these presumably must be paired in a queen to yield drive reversal in her progeny (pairing of wild-type *SB* with wild-type *Sb* yields supergene (*Sb*) drive; pairing leading to any of six different drive-locus^+^ or drive-locus^−^ homozygotes is expected to yield Mendelian proportions, although at least some of these homozygotes are expected to be lethal genotypes in queens; and pairing leading to either of two drive-locus^+^/drive locus^–^ heterozygotes on otherwise *SB* or *Sb* haplotypes in homozygous condition is expected to yield drive that is undetectable). (B) Sequence differentiation between *SB* (*N* = 60) and *Sb* (*N* = 20) social chromosomes from native (South American) *S. invicta*, as measured by differences in the pooled *SB* nucleotide diversity (π) and the median values of absolute sequence divergence (*d*_xy_) between individual chromosomes of each type along 5 kb non-overlapping windows. Values significantly greater than zero (substantially above blue line), consistent with recombination of *Sb* segments into *SB* haplotypes, are rare and involve small segments. Asterisks indicate three such instances of likely recombination of ≈5-10 kb-size elements. Positions of the three inversions contained within the supergene are shown by the blue bars. (C) Linkage disequilibrium (LD, measured as *r*^2^ values) in native *S. invicta* along the *SB* social chromosome (*N* = 60). Outside of the centromere, LD is weak, as expected given the lack of sizeable inversions and predicted resultant free recombination. Data for Panels B and C were obtained from whole-genome sequence assemblies (Y. Zheng et al., unpublished; [104]). Centromere is not shown to scale in any of the figures. (TIF 1.53 mb)

## Abbreviations

*k*: The proportion of gametes carrying the *Sb* drive (or another) allele
*M*_TRD_: Proportionate worker-induced selective mortality of embryos
TRD: Transmission ratio distortion
