# Supplementary material for: Unexpected patterns of segregation distortion at a selfish supergene in the fire ant Solenopsis invicta
Source: BMC Genet. 2018 Nov 7;19:101. doi: 10.1186/s12863-018-0685-9 (PMC6223060; doi:10.1186/s12863-018-0685-9)
Supplement: Supplementary file 1 — Table S1. Polygyne Solenopsis invicta nest information, mother queen characteristics, and numbers of embryos genotyped in progenies. (PDF 200 kb) [file 12863_2018_685_MOESM1_ESM.pdf]

Table S1 Polygyne *Solenopsis invicta* nest information, mother queen characteristics, and numbers of embryos genotyped in study progenies

Nest collection locality	Nest of origin of mother queen	Progeny code	Mother queen mating frequency	Marker loci: *Bertha*	*C27*	*C294*[a]	*C536*	*cassidy*	*Gp-9*[a]	*i_109*	*i_114*	*i_120*	*i_126*[a]	*i_129*	*red_ant*	*Sol-42f*	*Sol-49*	*sunrise*
Southeast Clarke Park, Athens-Clarke Co., Georgia, USA (33°55'33"N 83°18'30"W)	P2014-1	14-1-a	1	32 (0.189)	32 (0.430)	—	31 (0.237)	31 (0.237)	32 (0.108)	31 (0.298)	32 (0.298)	32 (0.108)	31 (0.055)	29 (0.132)	33	31 *116* (0.035)	31	32
		14-1-b	1	34	35	—	34 (0.568)	34 (0.061)	33 (0.500)	33 (0.364)	34 (0.432)	34 (0.061)	33	34	35	34 (0.196)	34 (0.196)	32 (0.189)
		14-1-c	2	35 *212* (0.045)	36	—	35 (0.155)	35 (0.500)	35 (0.500)	35 (0.088)	36	35 (0.500)	35 (0.500)	35 *154* (0.020)	36 (0.203)	35 (0.368)	35	36
		14-1-d	1	35 (0.229)	34	31 *92* (0.035)	35 (0.500)	33 (0.428)	36 (0.155)	35 (0.115)	27 (0.356)	35 (0.298)	33 (0.148)	35	—	33 (0.148)	34 (0.428)	35
		14-1-f	1	27	29 (0.430)	27 (0.360)	25	27	35 (0.368)	33 (0.570)	31 (0.212)	26 (0.500)	30	17	—	28 (0.298)	30 *137* (0.021)	32 (0.430)
		14-1-h	1	28	30 (0.075)	28 (0.356)	28 (0.425)	28	36 (0.309)	31	33 *303* (0.040)	28 (0.286)	31 (0.500)	28	—	28 (0.356)	28 (0.292)	31 (0.148)
		14-1-i	1	28 (0.286)	30 (0.292)	29 (0.092)	29 (0.286)	29	36 (0.203)	33 (0.360)	32 (0.181)	29 (0.286)	33 (0.141)	24	—	27 (0.575)	29	33 (0.237)
		14-1-j	1	28 *212* (0.044)	29 (0.292)	25 (0.229)	25 (0.356)	22 (0.356)	34 (0.121)	32 (0.500)	31 (0.430)	24 (0.356)	33 (0.081)	26 (0.154)	—	26 (0.500)	26 (0.229)	33 (0.148)
	P2014-3	14-3-a	1	31 (0.500)	32	31 (0.304)	32 (0.155)	33 *269* (0.002)	35 (0.309)	32 (0.368)	25	31	29	31	—	32 (0.364)	30 (0.196)	32 (0.368)
		14-3-b	1	24	24	22 (0.196)	16 (0.196)	24 (0.304)	26 (0.088)	25	24	33 *320* (0.040)	21	34 *154* (0.029)	—	33 (0.243)	19	26 (0.368)
		14-3-c	1	35 *206* (0.045)	35	—	35	35 (0.500)	35 (0.500)	35 (0.368)	34 (0.061)	35 (0.368)	32 (0.298)	35	36	35 (0.500)	35	32 (0.055)
		14-3-e	1	35 (0.360)	33	31 *106* (0.035)	34 (0.229)	34	35 (0.155)	35 (0.075)	31	33 (0.356)	31 *SB* (0.015)	34	—	33	35 (0.428)	35 (0.243)

Nest collection locality	Nest of mother queen	Progeny code	Mother queen mating freq.	*Bertha*	*C27*	*C294* [a]	*C536*	*cassidy*	*Gp-9* [a]	*i_109*	*i_114*	*i_120*	*i_126* [a]	*i_129*	*red_ant*	*Sol-42f*	*Sol-49*	*sunrise*
		14-3-f	1	33 (0.351)	33	32 (0.061)	31	30 (0.124)	36 *b* (0.045)	35 (0.570)	27 (0.428)	31	29 *Sb* (0.031)	32	—	31	30 (0.221)	34
		14-3-g	1	36 (0.500)	36	32 *92* (0.010)	34 (0.360)	36 (0.292)	36 *b* (0.033)	36 (0.368)	34 (0.221)	34 (0.360)	33	35	—	36	36 (0.181)	35 (0.196)
		14-3-h	1	36 (0.203)	35	—	36 (0.203)	36 (0.309)	36 (0.309)	36 (0.309)	36 *307* (0.033)	35 (0.250)	34 (0.196)	35	35	36 (0.566)	36 (0.121)	35 (0.368)
		14-3-i	1	29	29	30 (0.243)	29 (0.243)	28	36 (0.196)	33 (0.115)	32 (0.364)	30 *314* (0.049)	33 (0.196)	28	—	29 (0.364)	27	34
	P2014-4	14-4-a	1	29 (0.100)	31 (0.428)	31 (0.356)	31 (0.351)	30 (0.292)	35 (0.155)	34 (0.304)	29 (0.430)	32 (0.572)	33 (0.196)	33 (0.637)	—	33 (0.500)	30	34
		14-4-b	1	30	30	29 (0.416)	27 (0.402)	30	35 (0.423)	34 (0.212)	32 (0.345)	30	34 (0.332)	8	—	27	32 (0.180)	33
		14-4-e	1	28 (0.434)	32 (0.434)	25 (0.368)	26 (0.432)	26	36 (0.309)	32	23	31 (0.304)	31 (0.243)	23 (0.250)	—	29 (0.434)	28 (0.309)	32 (0.500)
		14-4-f	1	34 (0.286)	34	33 (0.500)	33 (0.423)	34 (0.279)	34 (0.434)	34 (0.298)	33 (0.339)	30	34 (0.500)	28 (0.500)	—	33 (0.500)	32 (0.286)	34
		14-4-g	1	29 (0.292)	31	29 (0.572)	28 (0.500)	28 (0.425)	36 (0.500)	33 (0.430)	33 (0.430)	28 (0.428)	33	28	—	29	30 (0.229)	33
		14-4-h	1	30 (0.356)	31	30 (0.428)	29	28 (0.286)	35 (0.566)	32 (0.500)	32 (0.430)	29 (0.428)	33 (0.500)	30	—	29 (0.356)	29 (0.124)	33
		14-4-j	1	36	36 (0.434)	—	36 (0.309)	36	31 *b* (0.035)	36 (0.434)	35 (0.500)	36 *314* (0.014)	36 *Sb* (0.033)	34	36 (0.121)	36 (0.121)	36	36
	P2014-13	14-13-a	1	35 *225* (0.020)	34	—	35 (0.500)	34	36 (0.121)	34 (0.304)	34 (0.568)	34 (0.432)	34	34	36	36	35 *162* (0.020)	36 (0.309)
		14-13-b	1	30 (0.292)	33 (0.364)	34 (0.304)	34	31 (0.500)	35 (0.250)	36 (0.203)	34 (0.432)	33 (0.243)	36	33 (0.243)	—	33 (0.364)	32	36 (0.121)
		14-13-c	1	30 (0.181)	30	29 (0.500)	27 *107* (0.026)	29 (0.356)	34 (0.432)	34 (0.115)	32 (0.108)	27 (0.351)	34 (0.432)	25 (0.212)	—	31 (0.500)	31 (0.141)	34
		14-13-d	2	34 (0.432)	35 (0.250)	34 (0.304)	34 (0.432)	35 (0.368)	36 (0.203)	36 (0.203)	36 (0.566)	33 (0.500)	36 (0.203)	33	—	34 (0.148)	34 (0.304)	36

Nest collection locality	Nest of mother queen	Progeny code	Mother queen mating freq.	*Bertha*	*C27*	*C294* [a]	*C536*	*cassidy*	*Gp-9* [a]	*i_109*	*i_114*	*i_120*	*i_126* [a]	*i_129*	*red_ant*	*Sol-42f*	*Sol-49*	*sunrise*
		14-13-f	2	35	35 (0.500)	—	35	35	36 (0.155)	35 (0.500)	34 (0.568)	35 (0.368)	34 (0.088)	35 (0.500)	35	35	35 *144* (0.045)	35
		14-13-j	1	32 (0.055)	17	—	32 (0.108)	31	32 (0.364)	32 (0.189)	32 (0.055)	17	32 (0.430)	17	32 (0.298)	32 (0.055)	32 (0.189)	32 (0.298)
		14-13-k	1	35 (0.500)	0	—	36 *87* (0.014)	36 (0.309)	36 (0.121)	36 (0.434)	36 (0.309)	36 (0.121)	36 (0.309)	36	35	36 *118* (0.006)	36 (0.434)	36
		14-13-m	1	36 (0.566)	36 (0.566)	—	36 (0.309)	0	36 (0.434)	36	36	36 (0.203)	36	36 (0.434)	36	36 (0.203)	36 (0.434)	36 (0.566)
		14-13-n	1	29	31	—	20 (0.252)	0	35 (0.368)	29	27 (0.425)	28	28 (0.425)	29	29	27	27 (0.500)	31
Wolfskin Road, Oglethorpe Co., Georgia, USA (33°51'00"N 83°14'36"W)	P2014-5	14-5-a	1	31	31	—	31 (0.500)	30 (0.428)	32 (0.570)	30	31	30	30	32	32	32	30 *144* (0.049)	31
		14-5-d	1	25	27	24 (0.500)	25 (0.212)	23 *275* (0.026)	28 (0.432)	26 (0.189)	25	25	26 (0.500)	24	—	24 (0.423)	24 (0.279)	28
		14-5-e	1	29 (0.292)	34	33 (0.148)	34 (0.500)	31 (0.108)	34 (0.115)	35 *154* (0.020)	32 (0.243)	33 (0.115)	34 *Sb* (0.029)	34	—	34 (0.500)	32 (0.081)	35 *85* (0.008)
		14-5-f	1	32 (0.500)	32	—	31 (0.141)	33	35 *b* (0.045)	33 (0.148)	33 (0.148)	33 (0.500)	32 (0.055)	31	33 (0.243)	33 (0.500)	33	33
		14-5-h	1	31	31 (0.500)	29 (0.229)	26	29	35 (0.368)	33 (0.364)	31 (0.500)	25 (0.500)	33 (0.243)	19 (0.324)	—	32 (0.108)	31 (0.075)	33
		14-5-j	1	31 (0.360)	33	—	31 (0.360)	31 (0.237)	32 (0.298)	32 (0.108)	31	31 *326* (0.035)	31	31	32	31 (0.141)	33	32
		14-5-k	1	36 (0.121)	36	36 (0.203)	35	36	36 (0.203)	35 (0.155)	33 (0.364)	34	35 (0.155)	9	—	36 (0.203)	36 (0.309)	35
		14-5-L	1	36	36	36 (0.066)	36 (0.309)	36 (0.566)	36 *b* (0.033)	36 (0.066)	36	36 (0.434)	36 (0.066)	36	—	35 (0.368)	36 (0.203)	36
		14-5-m	1	31 (0.237)	31 (0.237)	—	32 (0.430)	32 *239* (0.010)	33 *B* (0.040)	33 (0.148)	33 (0.243)	31	32 (0.055)	31	32 (0.298)	32 (0.298)	33 *144* (0.040)	33 (0.081)

Nest collection locality	Nest of mother queen	Progeny code	Mother queen mating freq.	*Bertha*	*C27*	*C294*[a]	*C536*	*cassidy*	*Gp-9*[a]	*i_109*	*i_114*	*i_120*	*i_126*[a]	*i_129*	*red_ant*	*Sol-42f*	*Sol-49*	*sunrise*
	**P2014-6**	**14-6-c**	**1**	31 (0.141)	30	—	27 (0.351)	30 (0.181)	36 (0.434)	31 (0.360)	31 (0.500)	31 (0.237)	30 (0.500)	30	34	31 (0.500)	31 (0.360)	32 (0.055)
		**14-6-f**	**1**	34 (0.432)	34	—	33 (0.148)	33 (0.148)	35 (0.250)	34 (0.115)	34	34 (0.196)	34	34	34 (0.061)	34 (0.432)	34 (0.304)	35 (0.155)
		**14-6-g**	**1**	33 (0.100)	34 (0.360)	34 (0.298)	31 (0.132)	35 (0.345)	35 (0.432)	35	35	34	34 (0.432)	32	—	35 (0.132)	34 (0.181)	35
		**14-6-i**	**1**	35	0	—	35 (0.368)	35 (0.250)	35 (0.250)	35	35 (0.250)	36 *320* (0.033)	35 (0.368)	35	35 (0.500)	35 (0.155)	35 *156* (0.045)	35
		**14-6-L**	**1**	36 (0.066)	36	36 (0.309)	36	36 (0.434)	36 (0.309)	36	36 (0.434)	36	36 (0.309)	36	—	36	36	36 (0.066)
		**14-6-n**	**1**	36 (0.434)	36	36 (0.434)	35 (0.500)	33	36 (0.566)	35 (0.155)	34 (0.196)	34	35 (0.368)	35 (0.155)	—	36 (0.203)	36 (0.309)	35 (0.088)
		**14-6-o**	**1**	32	29	27 (0.124)	32 (0.298)	30 (0.181)	35 (0.088)	33 (0.364)	25	29 (0.500)	28 (0.172)	27 *154* (0.001)	—	35	31 (0.360)	34 (0.304)
		**14-6-p**	**1**	33 (0.500)	34 (0.115)	34 (0.432)	34 (0.061)	32 (0.570)	35 (0.368)	33	33	32	32 (0.430)	33	—	33 (0.148)	33 (0.500)	34
		**14-6-r**	**1**	29	29 (0.500)	29 (0.356)	28 (0.575)	28	34 (0.304)	28 (0.172)	26 (0.423)	28 (0.575)	26 (0.423)	30	—	32	28	31
	**P2014-7**	**14-7-a**	**1**	32 (0.054)	30 (0.124)	31 *100* (0.032)	31 (0.500)	28 (0.339)	33 (0.092)	33 (0.423)	33 (0.500)	30	33 *SB* (0.038)	26	—	31 (0.419)	29 (0.419)	33
		**14-7-b**	**1**	32 *206* (0.010)	35	33 (0.148)	32 (0.141)	31 (0.141)	35 (0.148)	34	35 (0.196)	32	34 (0.196)	34	—	33	30 (0.075)	34 (0.115)
		**14-7-c**	**1**	34	34	—	34 (0.568)	33 (0.364)	36 (0.434)	34 *147* (0.029)	35	35 (0.250)	34 (0.304)	34	35	34	33 (0.243)	35 (0.088)
		**14-7-d**	**1**	24 (0.154)	29	25 *92* (0.007)	27 (0.351)	24 (0.581)	33 *b* (0.040)	31 (0.237)	31 (0.360)	25 (0.054)	30 (0.181)	28	—	28 (0.092)	28 (0.286)	31
	**P2014-8**	**14-8-a**	**1**	32	33	33 (0.360)	31 (0.237)	31 (0.172)	33 (0.243)	34 (0.500)	34 (0.148)	29 (0.428)	34 (0.243)	22	—	34 (0.500)	31 (0.132)	34
		**14-8-b**	**1**	30	31	33 *92* (0.040)	29	25 (0.360)	35 *b* (0.045)	33 *147* (0.029)	34	30 (0.570)	34 *Sb* (0.005)	33	—	29 (0.243)	30	34 (0.061)

Nest collection locality	Nest of mother queen	Progeny code	Mother queen mating freq.	*Bertha*	*C27*	*C294*[a]	*C536*	*cassidy*	*Gp-9*[a]	*i_109*	*i_114*	*i_120*	*i_126*[a]	*i_129*	*red_ant*	*Sol-42f*	*Sol-49*	*sunrise*
		14-8-d	1	35	34 (0.196)	—	32 (0.055)	34 (0.304)	35 (0.500)	34 (0.061)	35	34 (0.196)	35 (0.500)	34 (0.304)	34	34	34	34 (0.061)
		14-8-f	1	32 (0.108)	32	31 (0.075)	27 (0.351)	29 (0.500)	35 *B* (0.045)	34 *147* (0.001)	36 (0.121)	30 (0.181)	33 (0.148)	24	—	31 (0.141)	30 (0.428)	36 *85* (0.014)
		14-8-g	1 (2N male)	34	35	34 (0.304)	32 (0.237)	28 (0.088)	35 (0.304)	31 (0.500)	32 (0.155)	33 (0.061)	31 (0.304)	32	—	32 (0.250)	31 (0.368)	32 (0.500)
		14-8-i	1	32 (0.189)	32 (0.298)	32 (0.108)	32 (0.430)	32 (0.570)	32 (0.189)	32 (0.298)	32	32 (0.189)	32 (0.108)	32	—	32 (0.189)	32 (0.430)	32 (0.298)
		14-8-j	1	32 (0.189)	34 (0.196)	35 (0.500)	35 (0.250)	34	36 (0.434)	35 (0.250)	34 (0.568)	35 (0.250)	35 (0.500)	35	—	35 (0.500)	35 (0.368)	35
		14-8-k	1	35 (0.250)	36	35 (0.368)	36	35	36 (0.309)	36 (0.121)	36 (0.121)	36 (0.566)	36 (0.566)	34	—	35 (0.500)	36	36 (0.434)
		14-8-L	1	35 (0.088)	36	36 *92* (0.014)	36	36 (0.309)	36 *b* (0.014)	34 (0.432)	34 (0.115)	36 (0.309)	34 *Sb* (0.001)	0	—	36 (0.434)	36 (0.434)	35
Barnett Shoals/Whit Davis Roads, Athens-Clarke Co., Georgia, USA (33°53'11"N 83°19'06"W)	P2014-9	14-9-c	1	34	35 (0.368)	34 (0.432)	34 (0.304)	33 (0.364)	34 (0.432)	33 (0.243)	29 (0.356)	32 (0.108)	33 (0.243)	7 (0.500)	—	31 (0.360)	35 (0.368)	33 (0.500)
		14-9-e	1	36	36 (0.309)	36 *92* (0.014)	36 (0.434)	36	36 *b* (0.014)	35 (0.250)	35 (0.250)	36 (0.434)	35 *Sb* (0.020)	36	—	36 (0.309)	36 (0.434)	35 (0.155)
		14-9-g	1	36	26 (0.423)	—	36 (0.434)	36	36 (0.434)	36 (0.309)	36 (0.203)	35	36 (0.434)	35	36	36 (0.309)	36 (0.203)	36 (0.309)
		14-9-h	1	34 (0.061)	0	—	34 (0.568)	34 (0.061)	34 (0.432)	34 (0.196)	34	33 (0.364)	34 (0.432)	34	34	34 (0.196)	34 (0.196)	34
		14-9-i	1	35 (0.250)	35 (0.368)	—	35	35 (0.368)	34 (0.568)	35 (0.500)	34	35 (0.500)	35 (0.368)	35	35 (0.368)	35 *116* (0.045)	35 (0.250)	35 (0.500)
		14-9-j	1	33 (0.243)	32 (0.189)	—	33 (0.148)	31 (0.360)	33 (0.243)	33 (0.148)	34	32 (0.430)	34 (0.304)	32	34	31 (0.500)	31 (0.360)	34
		14-9-k	2	23 (0.202)	25	—	24	24	25 *B* (0.007)	24 (0.419)	24 (0.419)	25 (0.054)	24 *SB* (0.011)	24 (0.154)	17	24	23	25 (0.115)
		14-9-n	1	36	36	36 (0.203)	36 (0.566)	36 (0.434)	36 (0.203)	36	36 (0.203)	36 (0.434)	36 (0.203)	36	—	36 (0.434)	36 (0.434)	36
		14-9-o	1	35 (0.309)	35 (0.121)	35 (0.566)	35 (0.434)	35 (0.203)	36 (0.566)	35 (0.434)	35 (0.155)	35	35 (0.566)	35	—	35 (0.434)	35	35 (0.121)

Nest collection locality	Nest of mother queen	Progeny code	Mother queen mating freq.	*Bertha*	*C27*	*C294*[a]	*C536*	*cassidy*	*Gp-9*[a]	*i_109*	*i_114*	*i_120*	*i_126*[a]	*i_129*	*red_ant*	*Sol-42f*	*Sol-49*	*sunrise*
		14-9-p	1	35 (0.500)	35	—	0	0	36 (0.434)	35 ***154*** (0.020)	36 (0.434)	35 (0.368)	35 (0.368)	35	36 (0.121)	35 (0.155)	35 (0.155)	35
		14-9-r	1	36	36	—	36 (0.121)	0	35 ***b*** (0.020)	36	36 (0.566)	36 (0.203)	36 ***Sb*** (0.033)	36 (0.566)	36 (0.434)	36	36	36 (0.309)
	P2014-10	14-10-a	1	36 (0.309)	35	36 (0.203)	36 (0.203)	35	36 (0.203)	36 (0.434)	34	36 (0.566)	34 (0.304)	34 (0.568)	—	35 (0.088)	36 (0.203)	36
		14-10-b	1	32 (0.189)	33 (0.364)	32 (0.298)	32 (0.298)	32	33 (0.243)	30 (0.572)	29	33 (0.148)	28 (0.425)	32	—	32 (0.055)	32 (0.189)	30 (0.572)
		14-10-c	1	34	34	33 (0.148)	33 (0.243)	30 (0.572)	36 (0.121)	33	31 (0.430)	33	30 (0.181)	33	—	33 (0.500)	33 (0.500)	33 (0.364)
		14-10-d	1	33 (0.500)	33 (0.243)	33 (0.364)	33	33 ***269*** (0.007)	34 (0.368)	32 (0.430)	32 (0.430)	33 (0.364)	32 (0.430)	30	—	33 (0.364)	33	32
		14-10-e	1	33	32 (0.298)	32 (0.189)	32 (0.430)	30 (0.572)	33 (0.243)	34	33 (0.243)	32 (0.108)	34 (0.304)	26	—	32	31 (0.141)	35 (0.500)
		14-10-i	1	30 (0.572)	31	—	0	0	31 (0.360)	30	30 ***303*** (0.049)	30 ***326*** (0.021)	30 (0.292)	31 (0.360)	31	30 (0.181)	30 (0.181)	33 (0.432)
		14-10-j	1	33 (0.148)	34	—	33 (0.243)	18	36 (0.309)	33 (0.500)	32 (0.430)	33 (0.500)	33 (0.364)	33	33	33	33 (0.081)	33 (0.500)
		14-10-k	1	35 (0.250)	34	36 (0.434)	36 (0.309)	34	36 (0.434)	36	36 (0.309)	35 (0.368)	36 (0.434)	36	—	33 (0.081)	33 (0.500)	36
		14-10-L	1	32 (0.570)	32	—	32 (0.298)	0	33 ***b*** (0.018)	32 (0.430)	32 (0.189)	32 (0.298)	32 ***Sb*** (0.025)	32 (0.108)	33	31 (0.141)	31	32 (0.570)
	P2014-11	14-11-a	1	35 (0.500)	35 (0.368)	—	35 (0.088)	36 (0.434)	36 (0.566)	35 (0.368)	36 (0.121)	35 (0.155)	35 (0.250)	36	35	35 (0.250)	35	35
		14-11-b	1	33 (0.364)	33 (0.364)	33 (0.364)	34	31 (0.075)	35 (0.368)	36	35 (0.250)	33 (0.081)	35	34	—	33 (0.243)	33 (0.500)	36
		14-11-c	1	31 (0.243)	32	31 (0.304)	29 (0.189)	30 (0.351)	35 (0.250)	31 (0.428)	25 (0.237)	29	31 (0.428)	31	—	31 (0.237)	30 (0.500)	33
		14-11-e	1	34 ***225*** (0.029)	35	—	35 (0.250)	35	35 (0.088)	34	35	35	33 (0.148)	35	36	35 (0.368)	34 (0.568)	35 (0.155)

Nest collection locality	Nest of mother queen	Progeny code	Mother queen mating freq.	*Bertha*	*C27*	*C294*[a]	*C536*	*cassidy*	*Gp-9*[a]	*i_109*	*i_114*	*i_120*	*i_126*[a]	*i_129*	*red_ant*	*Sol-42f*	*Sol-49*	*sunrise*
		14-11-i	1	35 (0.566)	35 (0.309)	—	35	35 (0.434)	36 (0.066)	35 (0.434)	34 (0.155)	8 (0.254)	36 ***Sb*** (0.033)	8 (0.500)	35	35	35	34 (0.368)
		14-11-j	1	36	36 (0.121)	—	36 (0.309)	36 (0.434)	36 (0.566)	36 (0.309)	34	36	36 (0.434)	36 (0.566)	36 (0.434)	36 (0.309)	36 (0.066)	36 (0.203)
		14-11-k	2	35	35	35 (0.500)	35 (0.250)	35 (0.368)	35 (0.500)	34 (0.568)	33 (0.364)	35 (0.500)	34 (0.568)	35 (0.250)	—	35 (0.500)	35 (0.500)	34
		14-11-L	1	35	35	35 ***100*** (0.020)	34 (0.196)	35 (0.250)	36 ***B*** (0.014)	35 (0.368)	35	35 (0.088)	35 ***SB*** (0.020)	35 (0.500)	—	35 (0.500)	35 (0.500)	35
		14-11-m	1	34 (0.196)	34	33 (0.148)	33 (0.364)	33 (0.500)	36 (0.121)	34 (0.196)	31 (0.360)	33 (0.148)	32 (0.189)	34	—	35 (0.500)	35	33 (0.148)
	P2014-12	14-12-a	2	32	33	32 ***106*** (0.015)	30 (0.292)	32 (0.360)	35 (0.061)	33 (0.298)	30 (0.181)	31	31 ***SB*** (0.015)	31	—	32	32 (0.360)	33
		14-12-b	1	35 ***206*** (0.045)	34	35 (0.250)	35 (0.500)	33 (0.243)	35 (0.250)	35 (0.368)	35	33	36 (0.309)	34	—	34 (0.196)	32 (0.430)	36 (0.309)
		14-12-c	1	34	35	34 (0.432)	35 (0.500)	35 (0.088)	35 (0.500)	34 (0.304)	34	35 (0.088)	34 (0.568)	34 (0.196)	—	35	35 ***158*** (0.001)	34
		14-12-e	3	29 (0.500)	33	32 ***112*** (0.010)	32	30	34 (0.061)	33 (0.148)	33 (0.432)	31 (0.237)	34 ***SB*** (0.001)	32	—	31	30 (0.141)	35
		14-12-h	1	36 (0.121)	35	—	36 (0.203)	0	36 ***B*** (0.014)	36 ***145*** (0.006)	36 (0.203)	35 (0.250)	36 ***SB*** (0.014)	36 (0.566)	36	35 (0.368)	36 (0.309)	36
		14-12-j	1	35 (0.368)	36 (0.434)	—	35	26 (0.577)	36 (0.203)	36 (0.434)	36 (0.309)	33	36	35 ***146*** (0.045)	36 (0.309)	36 (0.203)	36 (0.434)	36
		14-12-k	1	35 (0.368)	9	—	35 (0.250)	27	34 ***B*** (0.029)	35	35 (0.250)	35 (0.368)	35 ***SB*** (0.045)	35 (0.368)	35	35 (0.500)	35 (0.500)	36 ***79*** (0.014)
		14-12-L	1	36	36	—	36 (0.203)	36 (0.566)	34 (0.432)	36 (0.566)	35	0	36	0	36	36	36	36

Nest collection locality	Nest of mother queen	Progeny code	Mother queen mating freq.	*Bertha*	*C27*	*C294*[a]	*C536*	*cassidy*	*Gp-9*[a]	*i_109*	*i_114*	*i_120*	*i_126*[a]	*i_129*	*red_ant*	*Sol-42f*	*Sol-49*	*sunrise*
		14-12-m	1	33 (0.061)	34	—	34 (0.500)	0	34 (0.088)	34 (0.368)	34 (0.368)	34	34	35	33	34 (0.368)	34 (0.500)	35
Number of progenies genotyped:				101	98	61	99	93	101	101	101	100	101	98	40	100	101	101
Mean number of embryos genotyped per progeny (excluding complete progeny failures):				32.7	32.6	32.1	32.3	31.7	34.5	33.6	32.6	31.9	33.1	30.8	34.0	32.9	32.5	33.9
Number of segregating progenies genotyped:				67	35	61	80	65	101	81	72	74	85	29	12	77	76	50
Number of segregating progenies with significant departures from Mendelian ratios (binomial $p<0.05$; as proportion of segregating progenies in parentheses):				7 (0.104)	0 (0)	11 (0.180)	2 (0.025)	4 (0.062)	17 (0.168)	6 (0.074)	3 (0.042)	6 (0.081)	17 (0.200)	4 (0.138)	0 (0)	3 (0.039)	7 (0.092)	3 (0.060)
Number of segregating progenies with significant departures from Mendelian ratios expected by chance in the absence of drive (5% of total progenies):				3.4	1.8	3.1	4.0	3.3	5.1	4.1	3.6	3.7	4.3	1.5	0.6	3.9	3.8	2.5

[a] Data for supergene-linked loci are highlighted in dark red font.

Dashes (—) indicate that progeny were not genotyped at a given locus. Blue shading indicates segregating progenies. Orange shading indicates segregating progenies with significant departures from Mendelian ratios (binomial test *p*-values in parentheses; over-represented alleles are underlined and italicized, with *i_126* alleles identified as occurring in the supergene haplotype [*Sb*] or in the homologous non-supergene haplotype [*SB*] by reference to *C294* and/or *Gp-9* alleles in the same gamete)
