# Supplementary material for: Unexpected patterns of segregation distortion at a selfish supergene in the fire ant Solenopsis invicta
Source: BMC Genet. 2018 Nov 7;19:101. doi: 10.1186/s12863-018-0685-9 (PMC6223060; doi:10.1186/s12863-018-0685-9)
Supplement: Supplementary file 2 — Text S1. Methods―additional information. (PDF 155 kb) [file 12863_2018_685_MOESM2_ESM.pdf]

Text S1 Methods—additional information

(a) Colony collection, rearing, and queen classification

Twelve large polygyne nests of *S. invicta* were collected in spring 2014 from three sites in northeastern Georgia, USA (Additional file 1: Table S1). Colony inhabitants were separated from the soil [1] and transferred to large plastic trays with moistened plaster-bottom nests held in a rearing room (14:10h light:dark cycle, 28-30°C, 40-70% RH; e.g., [2]). Colonies were provided water and fed daily by alternating a high-protein diet (tuna/dog food/peanut butter mix) with a high-carbohydrate diet (pureed assorted vegetables/granulated sugar mix), supplemented with frozen crickets provided on a twice-weekly basis [2, 3].

Wingless (reproductive) queens from each polygyne colony were isolated individually in small broodless fragments of their parent colony; these fragments consisted of approximately 3g (5000) adult workers housed in small plastic trays with small nests maintained as above [4]. By four weeks after setup, the brood patterns in each fragment allowed unmated queens to be distinguished from mated queens—worker brood were absent in the former but present in the latter. Unmated queens were discarded, whereas mated queens were used to produce progenies whose *Gp-9* and multilocus microsatellite genotype distributions were studied (see Additional file 3: Figure S1). Queens producing diploid males among their progeny were not distinguished from those producing only workers for the purposes of this study (see [5] for information on different classes of reproductive queens in polygyne *S. invicta*; see [6] for information on diploid male-producing queens).

(b) Collection of embryo progenies

Families (progenies) of diploid embryos were obtained from 101 mated mother queens in order to quantify transmission ratio distortion (TRD) (Additional file 3: Figure S1). Queens were isolated with 2-3 adult workers from their colony fragment in 6mL plastic specimen cups with moistened plaster bottoms (isolation cups); after 12h the queen was removed and frozen in a -80°C freezer. Eggs laid by the queen were maintained in the cup with the workers for an

additional 48h (untended eggs often succumb to mold infection [7]). These eggs (technically, embryos within the egg coat) were then collected with a fine artists' brush, transferred into a size "0" gelatin capsule, and immediately placed in a -80°C freezer. The age of collected embryos thus ranged from 48h to 60h post-oviposition; the normal developmental period from oviposition until eclosion of the embryo to the first instar larva in *S. invicta* is 120-144h at temperatures similar to those in our rearing room [8].

In a set of supplementary tests, we examined the aptitude of small groups of adult workers, such as those used to produce the embryo progenies in which TRD was assessed, to successfully maintain viable eggs/embryos for a period of 48h rather than cannibalize them or allow them to succumb to mold infection. Ten queens from each of four source colonies collected in the same area as the colonies used to estimate TRD were used in these supplementary tests. Single reproductive polygyne queens were held in a 10mm X 35mm petri dish for 12-24h—at this point they were removed, the eggs they laid were counted, their spermathecae were examined to ensure that they were mated, and they were confirmed to be *Gp-9* heterozygotes using the gel-based PCR method described below. A total of 1637 eggs (mean = 40.9 eggs laid/queen) were counted initially. Two or three workers from the same colony of origin as the queen were then placed in the petri dish units along with a small amount of high-carbohydrate diet. After 48h, all intact, evidently viable eggs/embryos in the dish were counted.

(c) DNA extraction and *Gp-9*/ microsatellite genotyping to quantify TRD

Frozen embryos were retrieved from gelatin capsules with a fine artists' brush and spread on a microscope slide. Thirty-six embryos per progeny were transferred individually with jewelers' forceps to single wells in 96-well assay plates containing 7μL ATL (tissue lysis) buffer (Qiagen). An additional 173μL ATL buffer and 20μL Proteinase K (Qiagen) solution were added to each well, and the plate was incubated overnight at 55°C. Following transfer of the contents of each well to a 1.5mL microcentrifuge tube, genomic DNA of the embryos was extracted using a DNeasy Blood & Tissue Kit (Qiagen) by following the manufacturer's instructions. Final DNA

elution was accomplished by adding 20μL AE buffer (Qiagen) heated to 65°C to each spin column, centrifuging the column, then repeating this step to recover a total 40μL of genomic DNA solution. After accounting for rare losses, a total of 3621 embryos were successfully extracted. DNA also was extracted from the heads of each of the 101 progeny mother queens, as well as twelve additional mother queens from the same source colonies whose progenies were not studied, by using a DNeasy Blood & Tissue Kit and following the manufacturer's instructions (final single elution to 200μL).

A multiplex PCR procedure modified from Valles and Porter [9] was used to score genotypes of individual embryos at *Gp-9*. Primers designed for this assay amplify all *Gp-9* allele *B* and allele *b* coding-sequence variants known from the US range of *S. invicta* [10, 11]; thus, all three major-allele genotypes (*BB*, *Bb*, *bb*) could be scored directly by running out the PCR products in agarose gels. Modifications to the procedure to increase its sensitivity given the small amounts of template DNA in each embryo were as follows. TaKaRa Ex Taq Hot Start DNA polymerase premix (Clontech; 2mM $MgCl_2$) was used in 30μL reaction volumes also containing 0.83μM of each of the four primers, 4μL of undiluted genomic DNA solution, and water. The following touchdown thermal cycling profile was employed: one cycle at 94.0°C (2min); followed by two cycles at 94.0°C (15s), 58.3°C (15s), and 68.0°C (45s); two cycles at 94.0°C (15s), 57.3°C (15s), and 68.0°C (45s); two cycles at 94.0°C (15s), 56.3°C (15s), and 68.0°C (45s); two cycles at 94.0°C (15s), 55.2°C (15s), and 68.0°C (45s); 32 cycles at 94.0°C (15s), 54.8°C (15s), and 68.0°C (45s); followed by a single final extension at 68°C (5min). Total volumes of the undiluted PCR amplicons were run out in 1.5% agarose gels, stained with ethidium bromide, and visualized under UV light. The same multiplex PCR procedure was used to score the *Gp-9* genotypes of the 113 mother queens, except the reactions were carried out in 15μL volumes with 2μL of genomic DNA solution diluted 1:20 (DNA:water) using a standard cycling profile [9].

Genotypes at 14 microsatellite loci (Additional file 4: Table S2) were scored using the stock genomic DNA solution from each embryo and the diluted DNA solution from each mother queen

as template in multiplex PCR reactions [11]. One primer of each locus primer pair was labeled at the 5′ end with one of four fluorescent dyes (FAM, PET, NED, VIC; Applied Biosystems). Primer pairs were combined in multiplex reactions by taking into account PCR thermal cycling profiles, dye labels, and expected size and yield of the PCR products. The complete set of 14 loci was amplified in three separate 12μL PCR reactions, each containing Hot-Start Taq 2X Mastermix (Denville Scientific), 0.06-0.4μM of each member of 2-5 pairs of primers, 2μL of DNA, and water. The thermal cycling profile was as follows: one cycle at 94°C (1min); followed by 35 cycles at 94°C (30s), primer-specific annealing temperature (45s), and 72°C (60s); followed by a single final extension at 72°C (40min). Resulting PCR amplicons were diluted (1:100 to 1:200) and pooled into a single plate for sequencer injection. GeneScan 600 LIZ size standard (0.1μL) was added to all pool-plex dilutions, which subsequently were run on an ABI-3730XL 96-capillary sequencer (Applied Biosystems). Microsatellite genotypes were scored from sequence chromatograms with the aid of the software GENEMARKER (SoftGenetics).

Any of the 3621 embryos that yielded weak or no detectable *Gp-9* PCR products using the above methods, but for which microsatellites could be scored, were subjected to a TaqMan qPCR (Applied Biosystems) allelic discrimination fluorogenic assay [12] in order to definitively confirm or assign *Gp-9* genotype. The 109 eggs for which neither *Gp-9* nor any of the microsatellites could be scored are assumed to be "non-embryonated eggs," which look normal for some period of time but fail to undergo gametogenesis and may serve a trophic function [5, 13].

The small amount of genomic DNA in our study embryos is highly unlikely to have fostered artifactual errors that affected our genotype scoring, such as may arise from allelic drop-out (non-amplification of one allele in heterozygotes) or from maternal DNA contamination. (i) Any artifactual scoring due to factors such as allelic drop-outs or contamination would give rise to multilocus genotypes in progenies that often appeared inconsistent with the known maternal

genotypes. Instead, we found that queen genotypes invariably were as expected given those of their embryos (eggs) in all 101 progenies. (ii) Artifactual scoring would generate spurious multilocus genotypes in progenies that mimicked patterns expected from frequent multiple paternity, but with the spurious genotypes confined to just one or a few loci per progeny and distributed sporadically among individuals. Instead, we observed only a low frequency of multiple paternity (as found in previous studies [4, 14, 15]), with the evidence for supernumerary patrilines consistent across many loci in each such progeny. (iii) Spurious embryo genotype calls would affect the twelve non-supergene loci as well as the three supergene loci, masking differences in frequencies of progenies with significant TRD between the two classes of markers; yet, we observed a pronounced difference in the average frequencies between the two classes. (iv) Allelic drop-outs or other factors leading to scoring artifacts would erode the strong congruence we observed between measures of recombination and gametic disequilibrium, as well as between these measures and the known genomic locations for all loci; moreover, spurious embryo genotype calls would undermine the concordant patterns of TRD we found among the three supergene loci.

(d) Data analyses

The multilocus *Gp-9* and microsatellite genotypes of diploid offspring embryos were used to infer the social chromosome and marker-locus phased haplotypes of the eggs giving rise to each embryo. Allele frequencies and expected heterozygosity (H_{exp}) were estimated for all 15 study loci from 113 mother queens and 109 of their male mates, the pairwise genetic relatedness coefficient (r) was estimated between each progeny-yielding mother queen and her mate(s) as well as between all pairs of nestmate queens (after excluding the three supergene-linked loci), and the fixation index F_{ST} was calculated as a measure of genetic differentiation between queens and their mates considered as groups (again after excluding the supergene-linked loci). Exact probabilities that the observed genotype frequencies at the 15 study loci conformed to Hardy

Weinberg equilibrium (HWE), as well as values of the inbreeding coefficient F_{IS}, were calculated for the 113 mother queens.

Associations between nestmate queen *r* and congruence in their *k* values (deviations from Mendelian segregation ratios) for the supergene were examined for pairs of queens as follows. A resampling method in which pairs of queens were randomly selected for each iteration was employed (each focal queen was used only once per iteration), with the resulting list of *r* values compared to the differences in supergene-linked *k* values for each pair (Δk) by calculating the Spearman correlation coefficient. Values of *k* represented the mean for the supergene-linked alleles at the three supergene loci. The procedure was run for 1000 iterations to generate a distribution and its 95% confidence limits for the correlation coefficient.

Maximum likelihood estimates of the pedigree recombination frequency (*c*) between each pair of marker loci were obtained by directly calculating the ratio of the number of recombinant to the total number of gametes (eggs) [16]. Estimates of the gametic disequilibrium coefficient D^* between locus pairs were calculated from the queen egg haplotypes represented in progeny embryos as well as for the haploid male mates of queens that produced study progenies. Calculated values of D^* were found to be highly correlated with those of other disequilibrium measures (i.e., D and D' [17]; data not reported).

We tested for significant TRD at each segregating locus within each progeny using one-tailed exact binomial tests (event probability $k = 0.5$) [18, 19]. Rather than evaluating statistical significance for each of the large number of these tests by adjusting the experimentwise α-value, we employed non-parametric resampling to generate confidence limits in order to minimize vulnerability to Type II errors [20, 21]. Specifically, the proportions of progenies with significant TRD at each locus were compared to the proportions expected under Mendelian segregation with a 5% Type I error rate using a combination bootstrap/subsampling (rarefaction) procedure (see e.g., [22]). This procedure involved drawing bootstrap samples of the minimum

number of segregating progenies for any locus (29 for locus *i_129*, disregarding locus *red_ant*, for which only twelve such progenies were genotyped); mean proportions for each locus were obtained from 1000 bootstrap replicates, with their one-tailed 95% confidence limits taken as the 95th percentiles of the bootstrapped proportions. For comparison, we also used a standard bootstrap procedure (without rarefaction subsampling) to estimate the proportions of progenies with significant TRD (and 95% confidence limits) for each locus. Because point estimates and their confidence limits obtained from the two types of bootstrap analyses were similar (Pearson r =0.999 and 0.945, respectively, both p<0.001), we present only results from the former.

We next conducted a simulation analysis to test whether observed segregation ratios at the four loci with the highest proportions of progenies with significant departures (based on binomial tests) were more extreme than expected by chance, given our specific sample sizes. An effectively infinite population pool of two gamete alleles in a 1:1 ratio (20,000 of each) was simulated, the number of gamete alleles equal to the actual progeny size was randomly drawn (with replacement) from this pool for each segregating locus in each progeny, and k was calculated; this procedure was then repeated 999 times, and the 97.5th, 95th, 5th, and 2.5th percentiles of the 1000 simulated k values were taken as the limits for statistical significance of the observed values in one- or two-tailed tests. For these and all subsequent tests involving calculation of k at the three supergene loci, k refers to the supergene alleles *92* at locus *C294* and *b* at locus *Gp-9*; for locus *i_126*, where recombination with the other supergene loci is higher (Fig. 1), the specific supergene-marking allele in a progeny was inferred by virtue of its association with the former two alleles. Importantly, in all 60 of the 85 progenies that segregated at *i_126* and included allele *230*, this allele was determined to mark the supergene.

The frequency and significance of TRD involving the *Sb* supergene across all 101 embryo progenies was evaluated further by considering the three supergene-linked loci simultaneously. The expected frequency of departures from Mendelian ratios at *Sb* occurring by chance in the absence of TRD, given our sample sizes, was estimated in a first multilocus simulation analysis

that accounted for the correlations in segregation ratios between these markers. Five progenies were designated at random to display significant TRD at *Gp-9* (the number expected due to Type I errors); each of these five progenies also was designated to display significant TRD at *C294* and *i_126* at probabilities 0.912 and 0.853, respectively, the empirically observed correlations in binomial probabilities of Mendelian ratios between these marker pairs (see main text). Progenies not assigned significant distortion at the latter markers by virtue of their association with *Gp-9* were designated at random to display significant distortion in order to yield cumulative totals of 4.25 and 3.0 progenies, respectively, departing by chance from Mendelian ratios (5% of the segregating progenies at each marker). The total number of unique progenies showing significant distortion at one or more supergene markers was tallied, and this procedure was reiterated 999 times to generate a distribution of numbers of progenies expected to exhibit non-Mendelian supergene ratios by chance. A second, far more conservative, multilocus simulation analysis that disregarded the correlations between supergene marker segregation ratios also was conducted. In this case, 5% of progenies at each locus were jointly designated at random as deviating from Mendelian segregation ratios in each of 1000 iterations, and the total number of unique progenies with significant distortion at one or more supergene markers was tallied for each iteration.

We next compared proportions of significant departures from 1:1 segregation ratios and distributions of *k* values between the supergene and non-supergene loci considered as separate classes. In a first set of analyses, we tested whether proportions of significant deviations from 1:1 ratios (determined by binomomial tests) differed between the two classes by conducting a permutation test in which differences in these proportions between paired loci belonging to the same or different classes were compared to differences between paired loci belonging to classes whose identity was randomly assigned (permuted). Specifically, differences between paired loci of the same supergene-associated status, either both supergene-linked or both not, as well as paired loci with each member in a different class, were compared to differences between paired

loci in which supergene association (class identity) of each member was assigned randomly; these assignments were constrained such that the numbers of within- and between-class pairs in the actual data were preserved in each permutation replicate (N = 69 and 36 pairs with members of the same and alternate supergene-associated status, respectively). Distributions of the differences obtained from the 1000 replicates conducted represent the null expectation when no difference exists in frequencies of significant departures from Mendelian ratios between supergene and non-supergene classes of loci. Non-parametric Mann-Whitney tests were employed to complement the permutation analyses; these involved comparing the observed differences in numbers of segregating progenies with $k \geq 0.65$ between paired markers of the same or alternate classes (for mean progeny sample sizes of 32-33 embryos genotyped per segregating locus, as in this study, k=0.65 is a general threshold level above which segregation ratios depart significantly from 1:1 according to the binomial test). In a second set of analyses, we tested whether distributions of the magnitude of departures from 1:1 ratios (unpolarized k values) differed between the two classes. A bootstrap test was conducted by constructing 5000 samples, in each of which the mean of the bootstrapped non-supergene k values was subtracted from the mean of the supergene values. The 95th percentile of the 5000 differences was taken as the one-tailed confidence limit for comparison with the expected difference of zero under the null hypothesis that supergene k values did not exceed those for non-supergene markers. This analysis was conducted using the online program STATKEY [23].

Finally, a resampling procedure was undertaken to estimate the population-wide frequencies of supergene-associated alleles within segregating progenies. A single embryo was drawn at random from each segregating progeny, then the embryo haplotype frequencies, along with the binomial probabilities of an even ratio of the alternate alleles, were calculated over the sample of segregating progenies. This procedure was repeated 999 times, with both the frequencies and binomial probabilities averaged over all resampling iterations.

References

1. Jouvenaz DP, Allen GE, Banks WA, Wojcik DP. A survey for pathogens of fire ants, *Solenopsis* spp. in the southeastern United States. Fla Ent. 1977;60:275-9.
2. Trible W, Ross KG. Chemical communication of queen supergene status in an ant. J Evol Biol. 2016;29:502-13.
3. Ross KG, Keller L. Experimental conversion of colony social organization by manipulation of worker genotype composition in fire ants (*Solenopsis invicta*). Behav Ecol Sociobiol. 2002;51:287-95.
4. Ross KG. Differential reproduction in multiple-queen colonies of the fire ant *Solenopsis invicta* (Hymenoptera:Formicidae). Behav Ecol Sociobiol. 1988;23:341-55.
5. Vargo EL, Ross KG. Differential viability of eggs laid by queens in polygyne colonies of the fire ant, *Solenopsis invicta*. J Insect Phys. 1989;35:587-93.
6. Ross KG, Vargo EL, Keller L, Trager JC. Effect of a founder event on variation in the genetic sex-determining system of the fire ant *Solenopsis invicta*. Genetics. 1993;135:843-54.
7. Tschinkel W. The fire ants. Cambridge, USA: Harvard University Press; 2006.
8. O'Neal J, Markin GP. Brood development of the various castes of the imported fire ant, *Solenopsis invicta* Buren (Hymenoptera: Formicidae). J Kansas Ent Soc. 1975;48:152-9.
9. Valles SM, Porter SD. Identification of polygyne and monogyne fire ant colonies (*Solenopsis invicta*) by multiplex PCR of *Gp-9* alleles. Insectes Soc. 2003;50:199-200.
10. Krieger MJB, Ross KG. Identification of a major gene regulating complex social behavior. Science. 2002;295:328-32.
11. Ascunce MS, Yang C-C, Oakey J, Calcaterra L, Wu W-J, Shih C-J, et al. Global invasion history of the fire ant *Solenopsis invicta*. Science. 2011;331:1066-8.
12. Shoemaker DD, Ascunce MS. A new method for distinguishing colony social forms of the fire ant, *Solenopsis invicta*. J Insect Sci. 2010;10:1-11.

13. Voss SH, McDonald JF, Bryan JHD, Keith CH. Abnormal mitotic spindles: developmental block in fire ant trophic eggs. Eur J Cell Biol. 1987;45:9-15.
14. Ross KG, Fletcher DJC. Comparative study of genetic and social structure in two forms of the fire ant, *Solenopsis invicta* (Hymenoptera: Formicidae). Behav Ecol Sociobiol. 1985;17:349-56.
15. Lawson LP, Vander Meer RK, Shoemaker D. Male reproductive fitness and queen polyandry are linked to variation in the supergene *Gp-9* in the fire ant *Solenopsis invicta*. Proc R Soc London B. 2012;279:3217-22.
16. Weir BS. Genetic data analysis II: methods for discrete population genetic data. Sunderland, USA: Sinauer; 1996.
17. Hedrick PW. Gametic disequilibrium measures: proceed with caution. Genetics. 1987;117:331-41.
18. Didion JP, Morgan AP, Clayshulte AM-F, Mcmullan RC, Yadgary L, Petkov PM et al. A multi-megabase copy number gain causes maternal transmission ratio distortion on mouse chromosome 2. PLoS Genet. 2015;11:e1004850.
19. Knief U, Schielzeth H, Ellegren H, Kempenaers B, Forstmeier W. A prezygotic transmission distorter acting equally in female and male zebra finches *Taeniopygia guttata*. Mol Ecol. 2015;24:3846-59.
20. Westfall PH, Young SS. Resampling-based multiple testing. New York, NY: Wiley; 1993.
21. Bender R, Lange S. Multiple test procedures other than Bonferroni's deserve wider use. BMJ. 1999;318:600-1.
22. Leberg PL. Estimating allelic richness: effects of sample size and bottlenecks. Mol Ecol. 2002;11:2445-9.
23. Lock RH, Lock PF, Morgan KL, Lock EF, Lock DF. Statistics: unlocking the power of data. 2nd ed. New York, NY: Wiley; 2017.
