# Supplementary figures and images for: Unexpected patterns of segregation distortion at a selfish supergene in the fire ant Solenopsis invicta
Source: BMC Genet. 2018 Nov 7;19:101. doi: 10.1186/s12863-018-0685-9 (PMC6223060; doi:10.1186/s12863-018-0685-9)

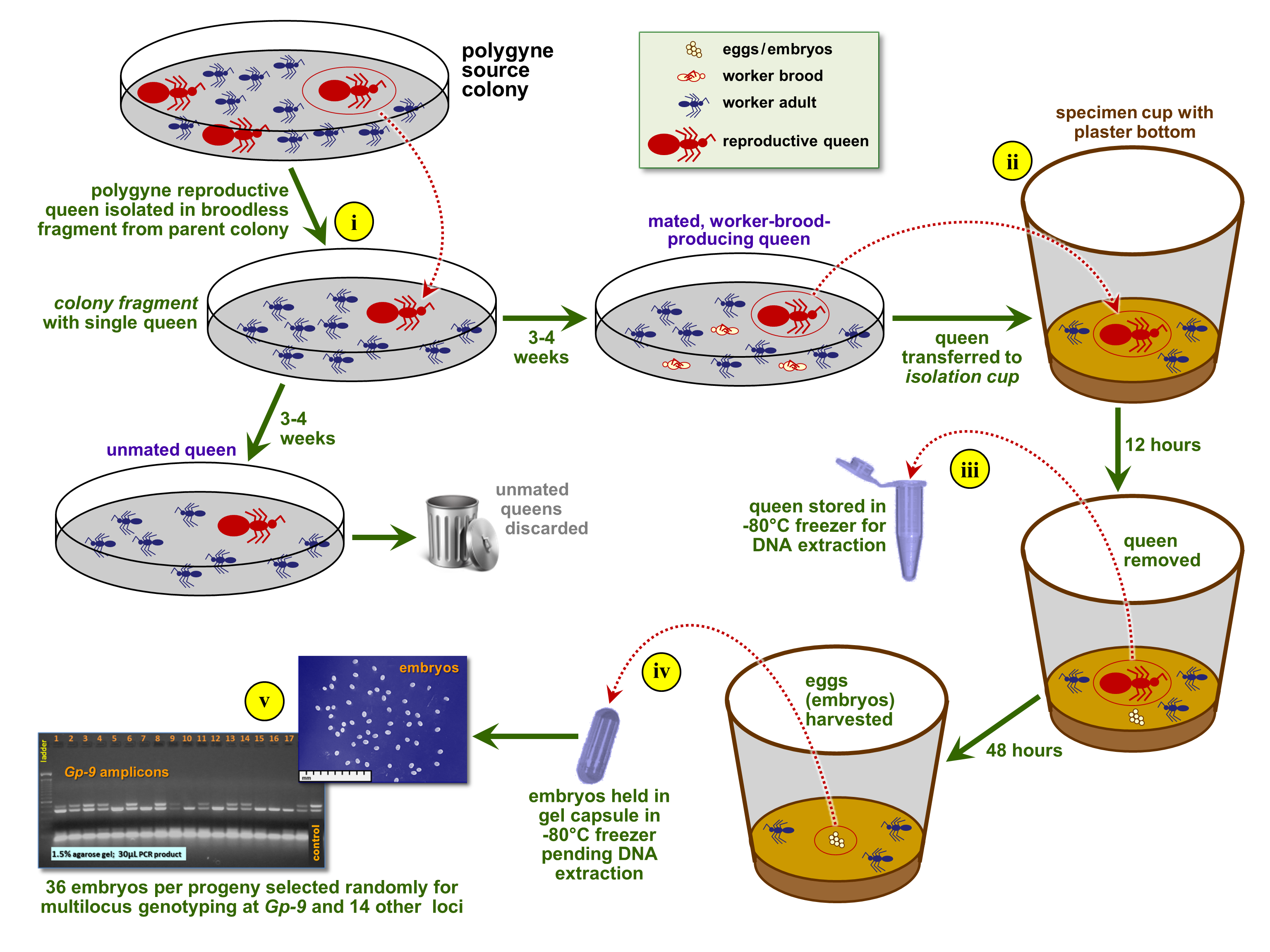

Supplement: Supplementary file 3 — Figure S1. Protocol for collecting progeny embryos for Sb supergene transmission ratio distortion (TRD) study in Solenopsis invicta. Families (progenies) of diploid embryos were obtained from individual mother queens initially isolated in plaster-bottomed petri dishes for three to four weeks with several thousand adult workers (colony fragments) (i). Queens confirmed to be mated (producing worker brood) at the end of this period were then isolated with 2–3 workers in plastic plaster-bottomed specimen cups (isolation cups) (ii). Each such queen was removed from the cup after 12 h then frozen in a − 80 °C freezer (iii). Eggs laid by the queen were maintained in the cup with the workers for an additional 48 h (by which time they were embryos within the egg coat), then transferred into a gelatin capsule and placed immediately in a − 80 °C freezer pending DNA extraction (iv). Thirty-six haphazardly selected embryos per progeny were sampled for genotyping at Gp-9 and 14 microsatellite loci (v). (TIF 1353 kb) [file 12863_2018_685_MOESM3_ESM.tif]

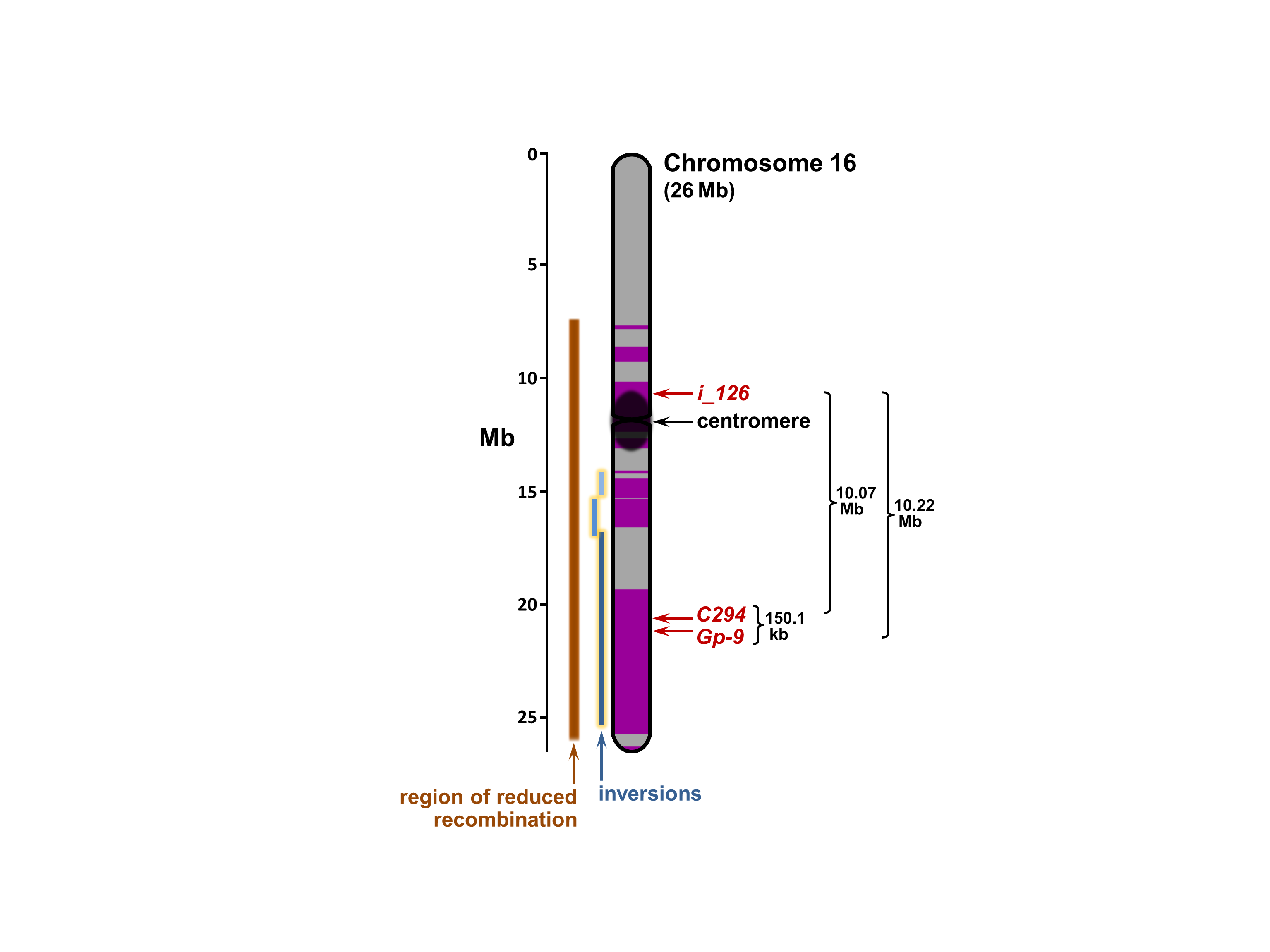

Supplement: Supplementary file 5 — Figure S2. The social chromosome (chromosome 16, Sb variant) of polygyne Solenopsis invicta. Depiction is based on reference genome build Si_gnH_C3 of a haploid SB male from the USA [51]. Locations of three inversions on the distal arm of the Sb chromosome (two of which overlap) are shown by blue bars, and the positions of three supergene-linked marker loci (i_126, C294, Gp-9) and the centromere are indicated (physical distances between loci are shown with parentheses; size of the centromere is not shown to scale). The region of reduced recombination on Sb chromosome 16, estimated by mapping to the new reference build 2796 RADseq SNPs from 92 haploid sons of a heterozygous SB/Sb queen [47], is indicated by the orange bar. Purple and grey blocks represent Pacific Biosciences (PacBio) contigs of the raw assembly. (Based on original figure by Y. Zheng.) (TIF 221 kb) [file 12863_2018_685_MOESM5_ESM.tif]

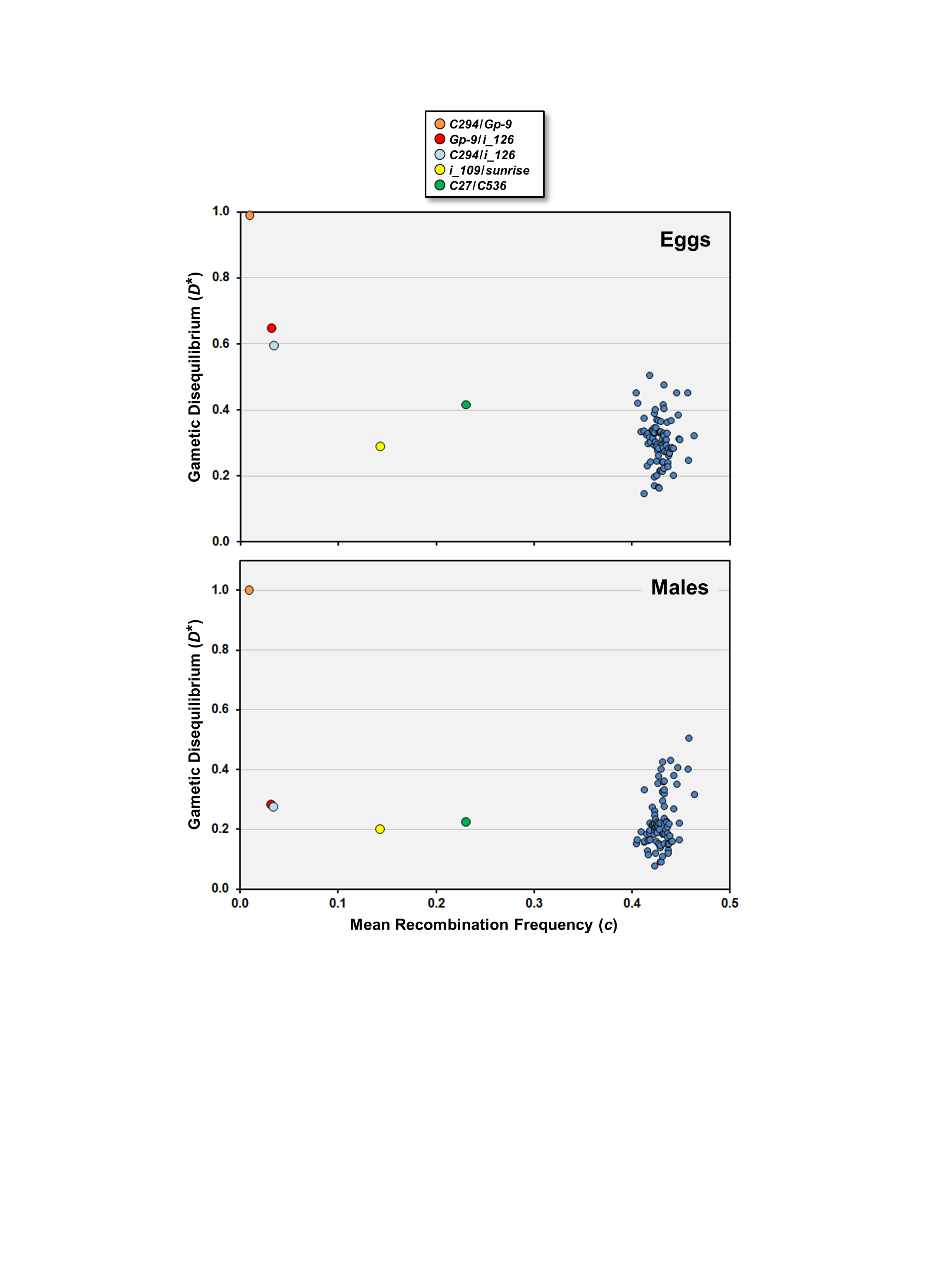

Supplement: Supplementary file 8 — Figure S3. Associations between recombination frequency (c) and gametic disequilibrium (D*) in progeny embryos (eggs) and males. The five locus pairs with values of c significantly less than 0.5 are indicated by the larger circles identified in the legend (the top three listed pairs are supergene loci). Locus pairs involving red_ant are excluded because of small sample sizes. (TIF 363 kb) [file 12863_2018_685_MOESM8_ESM.tif]

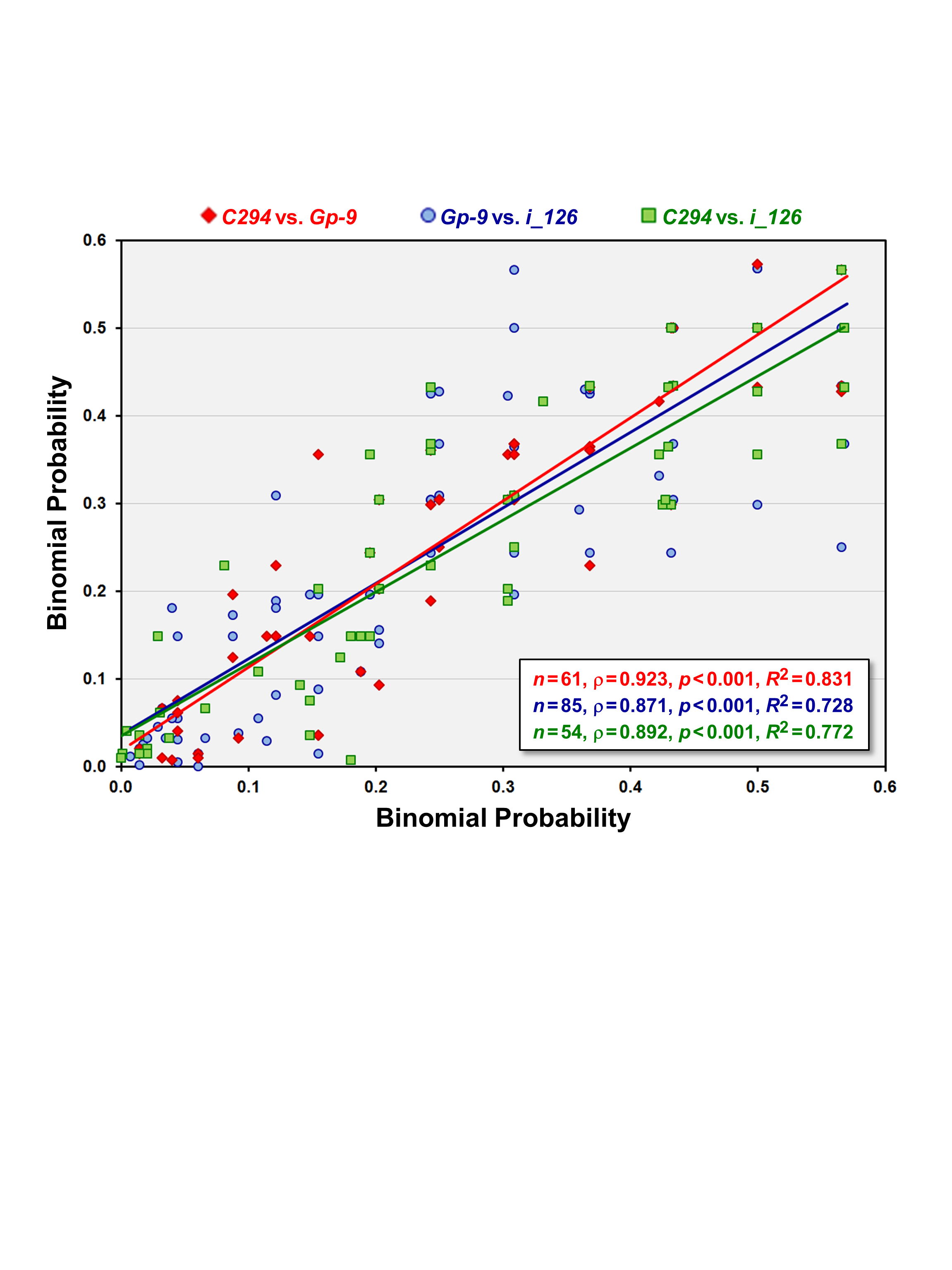

Supplement: Supplementary file 9 — Figure S4. Associations of binomial probabilities of Mendelian segregation ratios between supergene loci. Colored lines represent the least squares regression lines fitted to the three sets of values. Samples sizes, Spearman ρ and associated probability values (after Bonferroni correction for multiple tests), and R2 (coefficient of determination) values are shown in the inset. (TIF 669 kb) [file 12863_2018_685_MOESM9_ESM.tif]

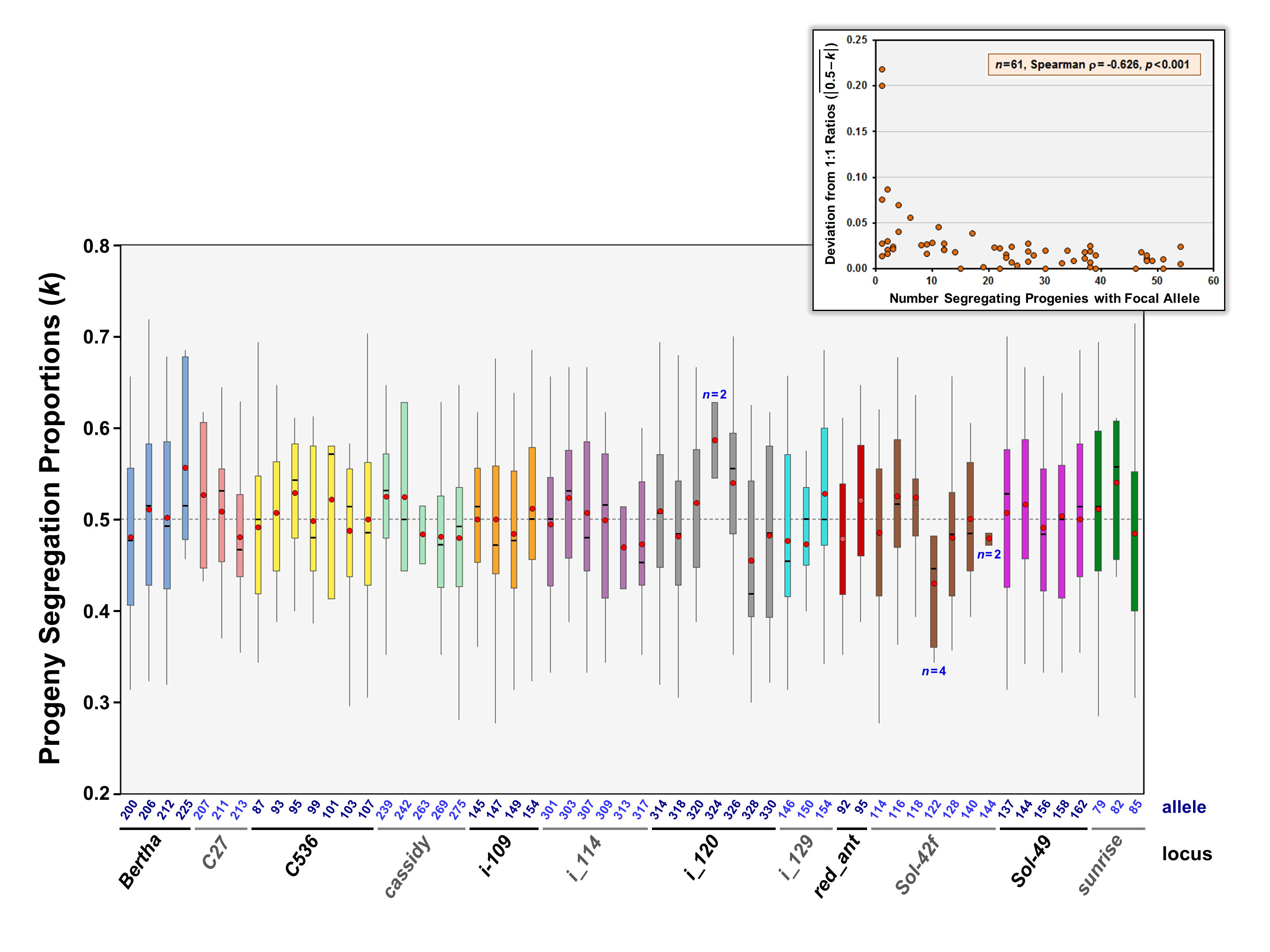

Supplement: Supplementary file 10 — Figure S5. Distributions of segregation proportions (k) in embryo progenies for 56 alleles of twelve non-supergene-linked microsatellite loci. Boxes represent interquartile ranges, whereas whiskers indicate the range limits. Within boxes, black horizontal bars represent the medians and red dots the means. Five alleles at four of the loci that segregated in only a single progeny are not included. The three alleles with boxes not overlapping 0.5 occurred in very few segregating progenies (numbers shown in blue). As expected for alleles that segregate in Mendelian ratios, estimates of the mean deviations from 1:1 ratios (│0.5 - k│) tend to decrease with larger numbers of segregating progenies studied (smaller plot). (TIF 1481 kb) [file 12863_2018_685_MOESM10_ESM.tif]

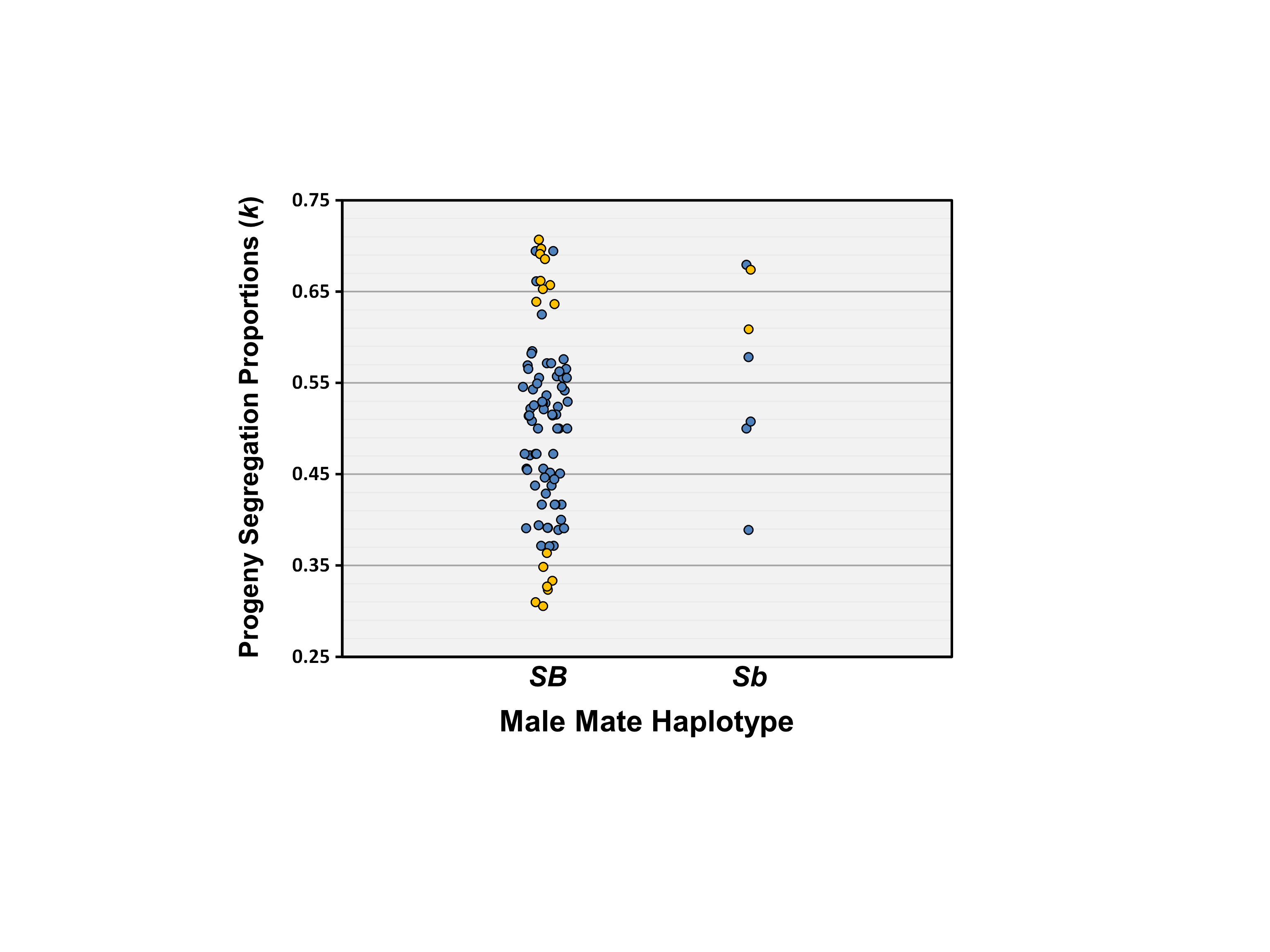

Supplement: Supplementary file 11 — Figure S6. Segregation proportions (k) in polygyne queen embryo progenies fathered by males lacking or bearing the Sb supergene. Values that differ significantly from 1:1 segregation ratios at one or more of the three supergene loci (binomial tests) are indicated by the yellow dots. The distributions of progeny k values, which represent weighted means across the supergene loci for each progeny, do not differ significantly with respect to the supergene-related haplotype of the fathers (Mann-Whitney test, N = 86 and 7, W = 3950, two-tailed p = 0.185). (TIF 157 kb) [file 12863_2018_685_MOESM11_ESM.tif]

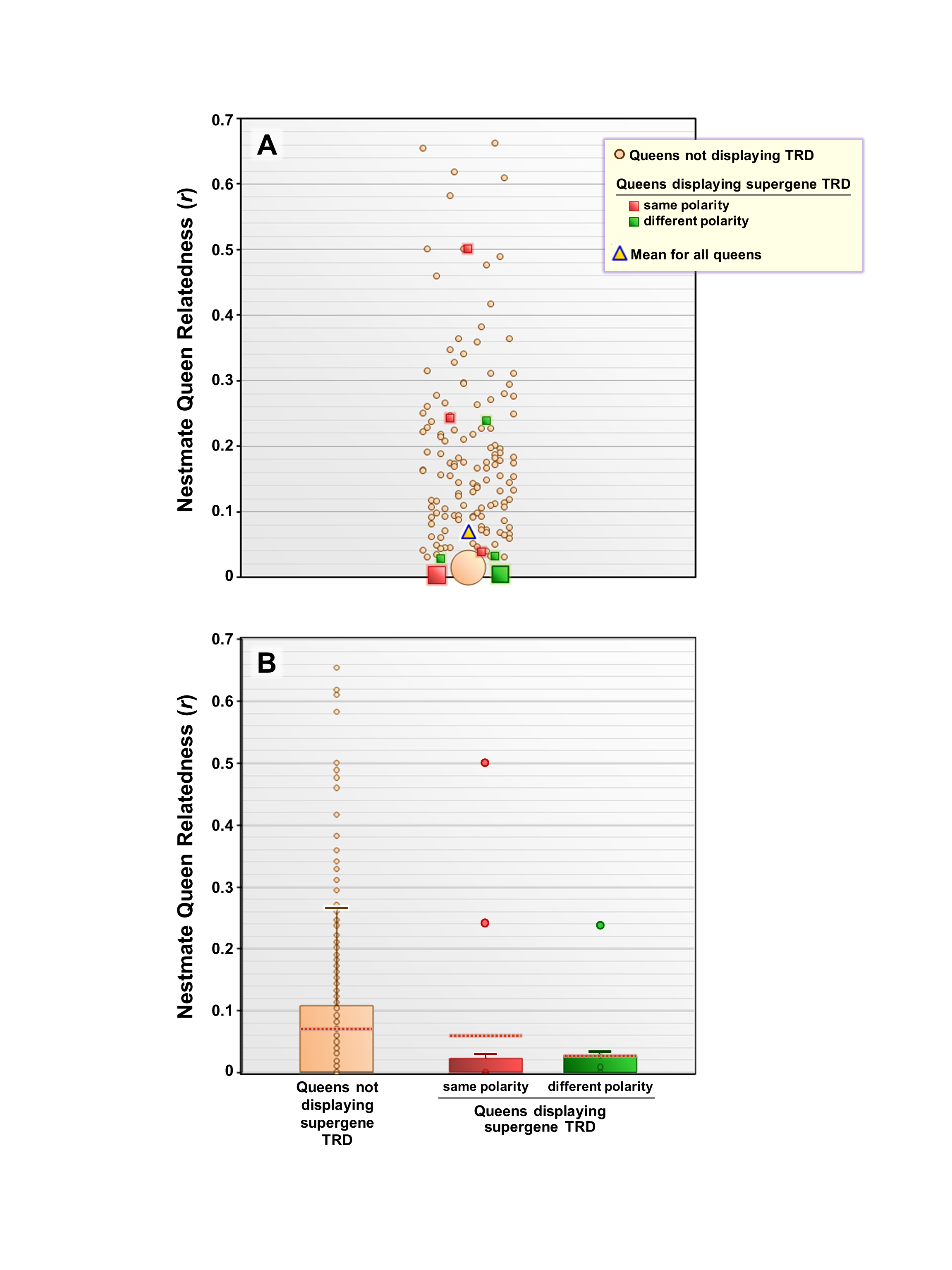

Supplement: Supplementary file 12 — Figure S7. Genetic relatedness between pairs of nestmate queens (r) in relation to their patterns of supergene TRD. Queen pairs are classified into three types: pairs in which one or both queens did not display significant TRD, pairs in which both queens displayed TRD of the same polarity (either both displayed drive or both displayed drive reversal), and pairs in which the queens displayed TRD of opposite polarity (one displayed drive and the other drive reversal) (N = 359, 12, and 11, respectively). (A) Distributions of r values for queens of the different types; sizes of the symbols indicate the relative numbers of identical r values (smallest symbols of each type, N = 1). Similar results showing that queens with significant TRD do not display unusually high or low relatedness to nestmate queens with TRD were observed as well for the seven individual source colonies from which these queens originated. (B) Box and whisker plots of the data. The boxes represent the interquartile ranges while the whiskers represent the 90th percentiles. Medians for each type are zero; means are indicated by the red dotted lines. Pairs of queens in which both members displayed TRD were neither significantly more nor less closely related to one another than were pairs of nestmate queens not displaying TRD (see main text). (TIF 631 kb) [file 12863_2018_685_MOESM12_ESM.tif]

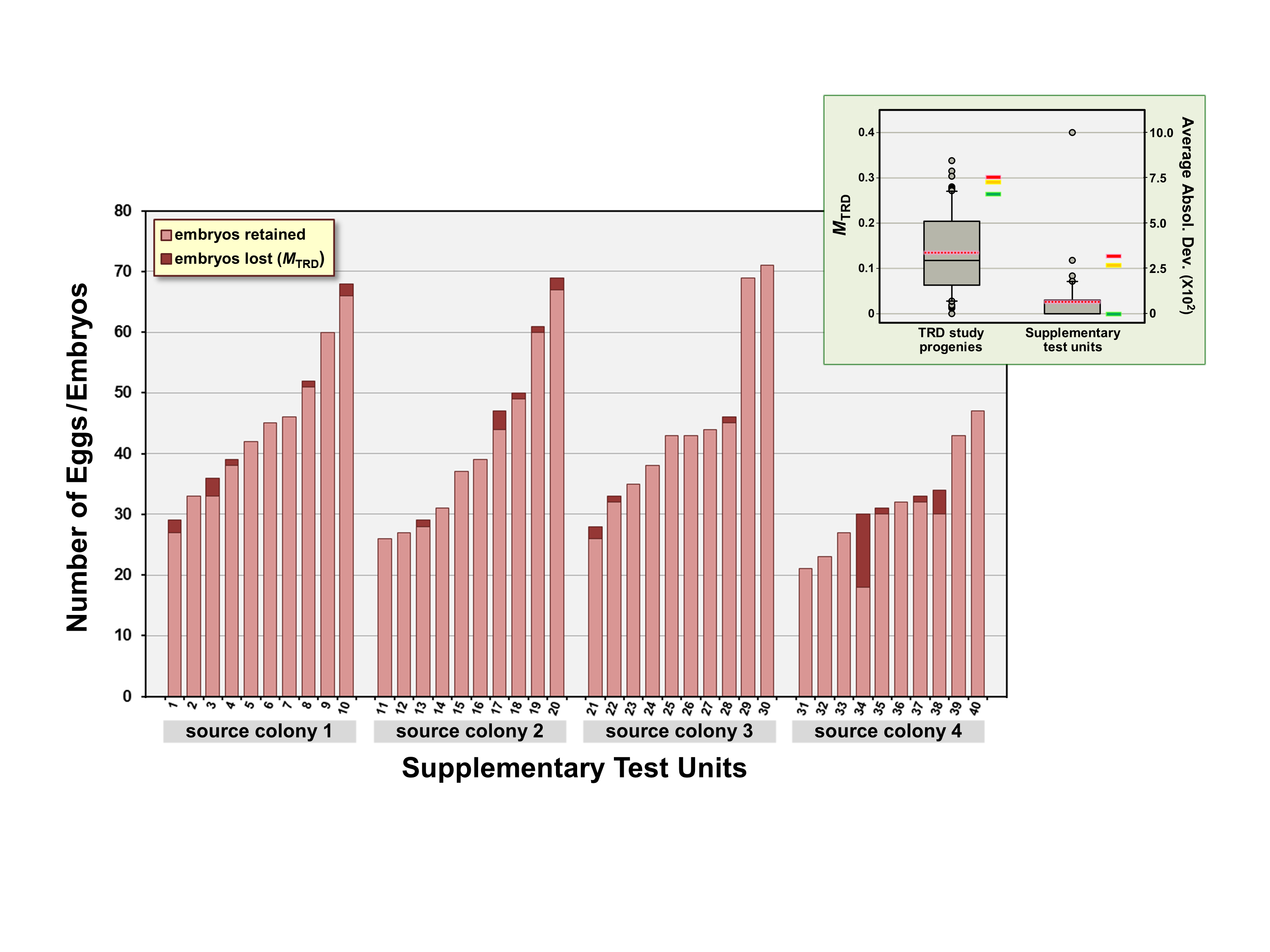

Supplement: Supplementary file 14 — Figure S8. Numbers of eggs/embryos successfully maintained by 2–3 polygyne workers over a 48 h period in supplementary tests. Dark red portions of the bars indicate the differences between initial and final numbers of intact eggs/embryos present in each test unit, corresponding to MTRD values if the losses are due to worker cannibalism (MTRD is defined as the proportionate worker-induced selective mortality of embryos that yields biased segregation ratios). The smaller plot contains boxplots comparing distributions of presumptive MTRD values for our 101 TRD study progenies and 40 supplementary test units. MTRD for the former was estimated from the unpolarized k values (see Additional file 6: Text S2 for formula), while for the latter it was directly equated with observed embryo losses. Boxes represent the interquartile ranges, while whiskers represent the 5th and 95th percentiles. Means of each distribution are indicated by the red dotted lines. Colored lines next to each box depict values for three summary measures of statistical dispersion in MTRD values for each group (collectively termed Average Absolute Deviation statistics); these are the Mean Absolute Deviation from the Mean (red), Mean Absolute Deviation from the Median (gold), and Median Absolute Deviation from the Median (green). The boxplots and dispersion statistics show that MTRD values in our supplementary test units are too low and unvarying to support the hypothesis that worker intervention during embryogenesis rather than segregation distortion (meiotic drive) primarily caused significant supergene TRD in our main experiment. (TIF 1564 kb) [file 12863_2018_685_MOESM14_ESM.tif]

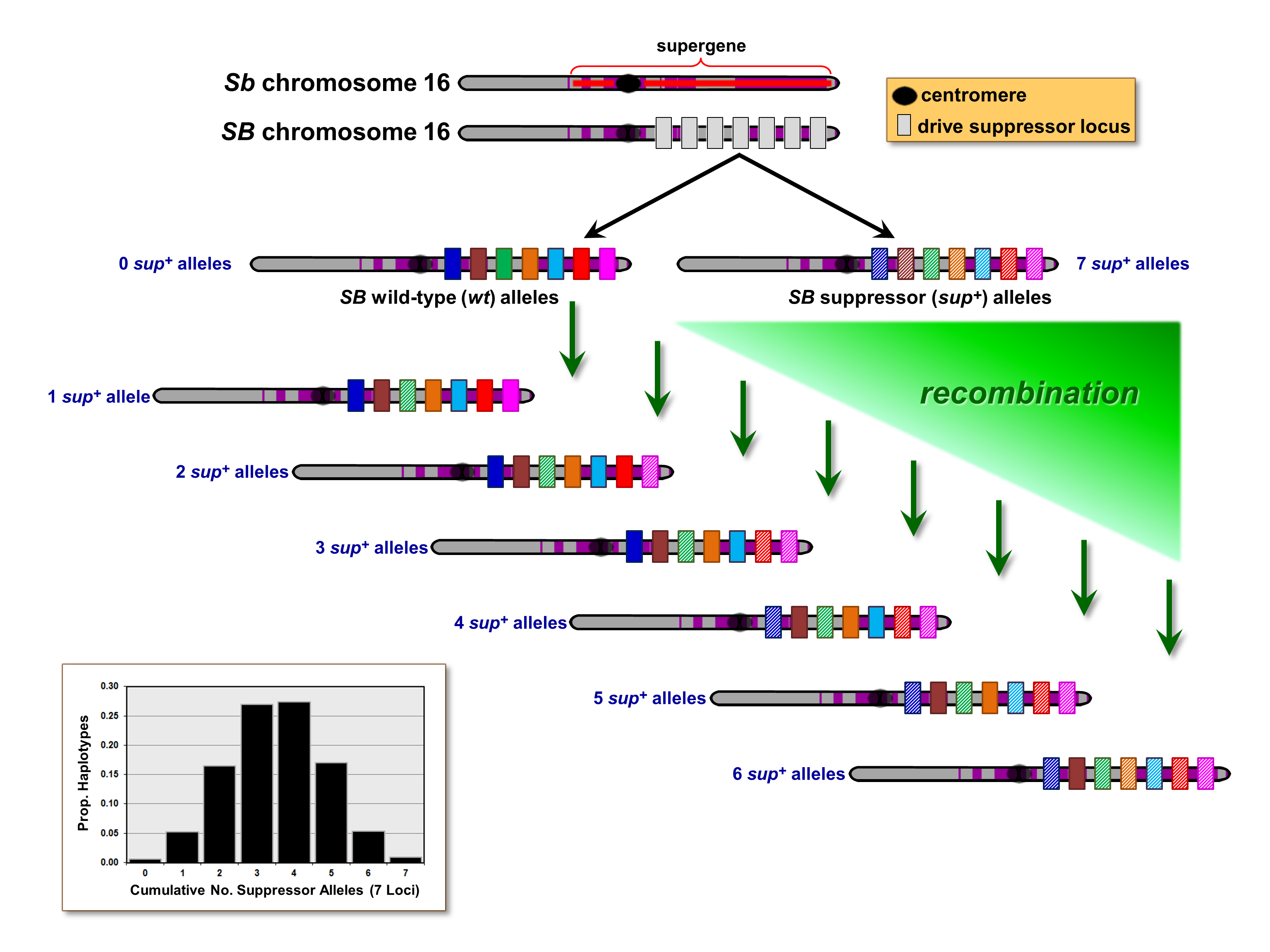

Supplement: Supplementary file 15 — Figure S9. Hypothetical scenario for additive genetic mechanism of Sb supergene drive reversal in polygyne Solenopsis invicta. The presence of seven suppressor loci segregating wild-type alleles (ineffective in suppressing Sb drive) along with suppressor alleles is depicted as an example of such a multilocus system, with several different multilocus haplotypes arising from recombination and segregation in the population illustrated. The inset depicts the distribution of multilocus haplotypes with varying proportions of suppressor alleles expected in an equilibrium population of 5000 haplotypes with the seven suppressor loci in gametic equilibrium, equal allele frequencies, and no selection. Haplotypes with no or very few suppressor alleles allow Sb drive to prevail, those with high or maximal numbers of suppressor alleles overwhelm Sb drive to cause drive reversal, and those with intermediate proportions of suppressor alleles (the most common circumstance―inset) neutralize Sb drive to favor Mendelian segregation ratios. The suppressor loci are shown in the region of SB chromosome 16 homologous to the supergene (see Additional file 13: Text S3 for rationale). Centromere is not shown to scale (see Additional file 5: Figure S2). (TIF 781 kb) [file 12863_2018_685_MOESM15_ESM.tif]

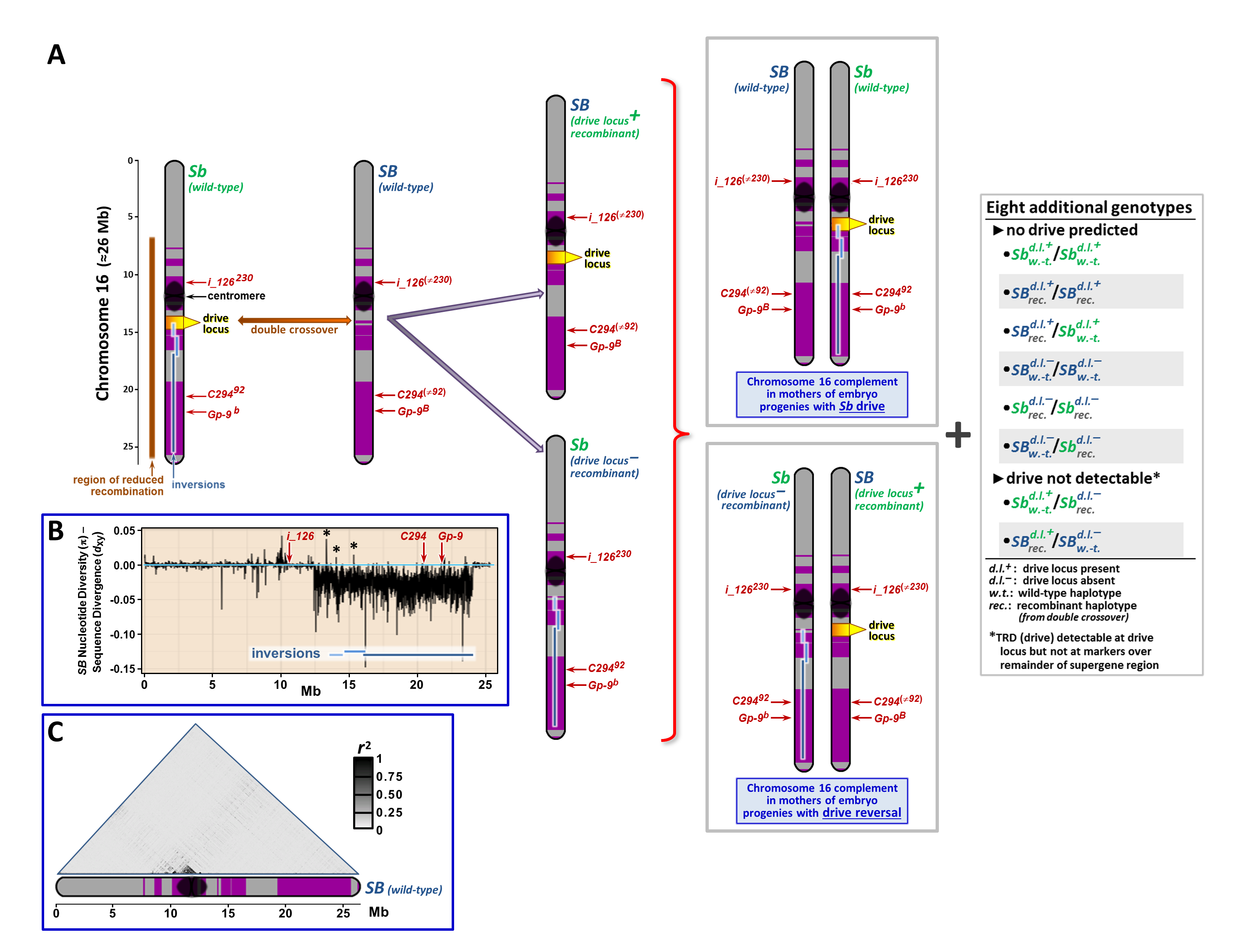

Supplement: Supplementary file 16 — Figure S10. Hypothetical scenario for a historical double crossover involving the putative supergene drive locus as the genetic mechanism responsible for Sb supergene drive reversal in polygyne Solenopsis invicta. (A) Double crossover between the wild-type Sb and SB social chromosomes in the segment between markers i_126 and Gp-9 transferred the complete drive complex (locus) from Sb to SB without altering the congruence of alleles at all three supergene markers observed in progenies with both drive and reversal-of-drive. Two novel recombination products were generated, a SB chromosome containing the drive locus and a Sb chromosome without it, and these presumably must be paired in a queen to yield drive reversal in her progeny (pairing of wild-type SB with wild-type Sb yields supergene (Sb) drive; pairing leading to any of six different drive-locus+ or drive-locus− homozygotes is expected to yield Mendelian proportions, although at least some of these homozygotes are expected to be lethal genotypes in queens; and pairing leading to either of two drive-locus+/drive locus– heterozygotes on otherwise SB or Sb haplotypes in homozygous condition is expected to yield drive that is undetectable). (B) Sequence differentiation between SB (N = 60) and Sb (N = 20) social chromosomes from native (South American) S. invicta, as measured by differences in the pooled SB nucleotide diversity (π) and the median values of absolute sequence divergence (dxy) between individual chromosomes of each type along 5 kb non-overlapping windows. Values significantly greater than zero (substantially above blue line), consistent with recombination of Sb segments into SB haplotypes, are rare and involve small segments. Asterisks indicate three such instances of likely recombination of ≈5-10 kb-size elements. Positions of the three inversions contained within the supergene are shown by the blue bars. (C) Linkage disequilibrium (LD, measured as r2 values) in native S. invicta along the SB social ch [file 12863_2018_685_MOESM16_ESM.tif]
