# Supplementary material for: Unexpected patterns of segregation distortion at a selfish supergene in the fire ant Solenopsis invicta
Source: BMC Genet. 2018 Nov 7;19:101. doi: 10.1186/s12863-018-0685-9 (PMC6223060; doi:10.1186/s12863-018-0685-9)
Supplement: Supplementary file 4 — Table S2. Characteristics of marker loci genotyped in polygyne queens and their progeny embryos (male genotypes inferred). (PDF 10 kb) [file 12863_2018_685_MOESM4_ESM.pdf]

Table S2 Characteristics of marker loci genotyped in polygyne queens and their progeny embryos (male genotypes inferred)

Locus[a]	Number of alleles (N_a)			Expected heterozygosity (H_{exp})			Chromosome	Genome start coordinate[c]
	Queens	Males[b]	Both sexes	Queens	Males[b]	Both sexes		
Bertha	5	3	5	0.653	0.592	0.625	3	3:10321677
Sdag_C27 (*C27*)	3	3	3	0.327	0.351	0.338	6	6:10867177
Sdag_C294 (*C294*)	7	6	7	0.642	0.727	0.724	16	16:18429274
Sdag_C536 (*C536*)	8	8	8	0.820	0.813	0.819	6	6:5725253
cassidy	5	4	5	0.664	0.659	0.661	7	7:12459354
Gp-9	2	2	2	0.500	0.209	0.432	16	16:18579940
Sol_i109 (*i_109*)	4	4	4	0.736	0.714	0.726	14	14:2617730
Sol_i114 (*i_114*)	6	6	6	0.713	0.731	0.720	5	5:7887511
Sol_i120 (*i_120*)	7	6	7	0.711	0.687	0.704	10	10:6961043
Sol_i126 (*i_126*)	5	4	5	0.751	0.682	0.743	16	16:8362602
Sol_i129 (*i_129*)	4	3	4	0.286	0.287	0.286	4	4:10716117
red_ant	2	2	2	0.352	0.232	0.294	6	6:18309984
Sol-42f	10	7	11	0.782	0.774	0.778	15	15:6834468
Sol-49	7	5	7	0.758	0.705	0.733	8	8:9915806
sunrise	3	3	3	0.496	0.486	0.490	14	14:5031308

[a] All loci except *Gp-9* are microsatellites. Data for supergene-linked loci are highlighted in red shading and font. Abbreviated locus names used in the main text and display items (if any) are shown in parentheses

[b] Alleles of mates of polygyne queens were inferred from comparison of maternal and progeny genotypes

[c] Based on reference genome build *Si_gnH_C3* of a haploid *SB* male from the USA (Y. Zheng et al., unpublished)
