# Supplementary material for: Unexpected patterns of segregation distortion at a selfish supergene in the fire ant Solenopsis invicta
Source: BMC Genet. 2018 Nov 7;19:101. doi: 10.1186/s12863-018-0685-9 (PMC6223060; doi:10.1186/s12863-018-0685-9)
Supplement: Supplementary file 6 — Text S2. Results―additional information. (PDF 144 kb) [file 12863_2018_685_MOESM6_ESM.pdf]

Text S2 Results—additional information

(a) Marker and sample characteristics

Based on the observed genotypes of 113 reproductive queens and inferred genotypes of 109 of their male mates, each microsatellite locus possessed 2-11 alleles (mean = 5.5), with the per-locus H_{exp} ranging from 0.286 to 0.819 (Additional file 4: Table S2). The gene *Gp-9* segregates the alternate alleles, *B* and *b*, in our study population and other polygyne populations in the USA; all reproductive queens of this form are heterozygotes, the majority of which mate with haploid *B* males (see below and [1, 2]). Consistent with this, H_{exp} at *Gp-9* was estimated at 0.5 for queens but somewhat less than this value for queens and males combined (Additional file 4: Table S2).

The numbers of progenies in which embryos were genotyped ranged from 40 for locus *red_ant* to 101 for six of the microsatellite markers as well as *Gp-9* (Additional file 1: Table S1). The mean numbers of embryos successfully genotyped per progeny at each marker locus are listed in Table 1. The overall mean number of embryos scored per progeny for the microsatellites was 32.6 (excluding progenies for which a marker failed entirely), with a mean of 34.5 embryos scored for *Gp-9* across the 101 progenies. The numbers of progenies segregating for each microsatellite locus ranged from twelve to 85 (Table 1, Additional file 1: Table S1). All 101 progenies segregated the two *Gp-9* alleles because, as expected, all mother queens were confirmed *Bb* heterozygotes. Importantly, heterozygosity with the expected alleles was confirmed for the mother queens at all microsatellite markers that segregated in their progenies; indeed, observed queen genotypes invariably were consistent with those of their embryos in all progenies.

(b) Progeny characteristics

Based on comparisons of queen and offspring embryo multilocus genotype distributions, several different types of progenies were recognized. The great majority of mother queens (94 of 101; 93.1%) mated with a single male, so their progenies comprised simple families. Of these

monandrous queens, most (86 of 94; 91.5%) mated with a male bearing the *B* allele at *Gp-9* (i.e., lacking the supergene). Among the remaining monandrous queens, seven (7.4%) mated with a *Gp-9*^{b}-bearing male (i.e., a male whose chromosome 16 bore the *Sb* supergene), whereas a single queen (1.1%) mated with a fertile diploid male that was heterozygous at *Gp-9*. All of the offspring of this latter queen were judged to be triploids [3], based on the presence of three alleles in single embryos at several of the microsatellite loci (in all embryos at locus *Sol-42f*) and invariably uneven band intensities observed for all individuals in the gel-based *Gp-9* PCR assay. In this triploid progeny, the patterns of differential band intensity allowed unequivocal assignment of queen gamete haplotypes at *Gp-9*, while comparison of queen and offspring genotypes allowed assignment of queen gamete haplotypes at the microsatellite loci.

Only seven of the 101 mother queens (6.9%) mated multiply, one of these polyandrous queens (14.3%) evidently with three males and the remainder (85.7%) with two males. Only two of the polyandrous queens (28.6%) mated exclusively with *Gp-9*^{B}-bearing males, while the remaining five (71.4%) mated with males of each *Gp-9* haplotype. This result, along with the rarity of matings by monandrous queens to *Gp-9*^{b} males, is consistent with earlier suggestions that queens of invasive polygyne *S. invicta* that mate initially with a *Gp-9*^{b} male typically remain receptive to remating, whereas those that mate with a *Gp-9*^{B} male tend not to remate [2].

The pairwise coefficient of genetic relatedness (r) estimated between queens and their mates (supergene-linked loci excluded) ranged from 0 to 0.324, with a mean of 0.041, median of zero, and bootstrap 95% confidence interval (CI) of 0.029-0.055; no effect of nest of origin of the queens on r between mates was found ($N = 107$, Kruskal-Wallis test, $H = 15.1$, $p = 0.18$). Genetic differentiation between successful reproductives of the two sexes considered as groups also was minimal, with single-locus estimates of F_{ST} between mother queens and their mates ranging from -0.007 to 0.013 at the non-supergene-linked loci. Estimates of pairwise nestmate queen r ranged from 0 to 0.662, with a mean of 0.069, median of zero, and bootstrap 95% CI of

0.058-0.082; again, no effect of nest of origin on these *r* values was detected (N = 382, Kruskal-Wallis test, H = 8.7, p = 0.65).

Importantly, queens with significant supergene transmission ratio distortion (TRD) were neither less nor more closely related to nestmate queens also displaying TRD compared to queens in pairs not displaying TRD—this was true whether polarity of TRD was the same for the focal pair (both displayed drive or drive reversal) or differed between them (one displayed drive and the other drive reversal) (Kruskal-Wallis test, H = 0.87, p = 0.65; the three classes compared were queen pairs for which one or both queens did not display TRD, pairs with TRD of the same polarity, and pairs with TRD of opposite polarity; see also Additional file 12: Figure S7). Also, relatedness of pairs of nestmate queens was not correlated with similarity in their supergene *k* values (mean Spearman ρ= 0.084 over 1000 iterations of a randomization test, 95% CI for ρ: -0.101–0.262); that is, more closely related queens did not tend to have more congruent levels of deviation from Mendelian ratios at the supergene loci. The relevance of these findings with respect to a potential mechanism of drive reversal is discussed in Additional file 13: Text S3.

(c) Recombination and linkage disequilibrium

Estimation of the pedigree recombination frequency (*c*) between pairs of marker loci was accomplished by directly counting the number of recombinant gametes (eggs) represented in each progeny. Values of *c*, along with their 95% confidence intervals (CIs) and minimum and maximum values, are depicted in Fig. 1. The 95% CIs of only five pairs of markers do not overlap with 0.5, the value for freely recombining loci. The three lowest estimates of recombination involve pairwise comparisons of the three supergene-linked markers (mean c = 0.009, 0.032, and 0.034; N = 62, 85, and 55 progenies for *C294*/*Gp-9*, *Gp-9*/*i_126*, *C294*/*i_126*, respectively; see Additional file 5: Figure S2 for locations of these markers on chromome 16). The two other pairs of loci with *c* significantly less than 0.5 are *i_109*/*sunrise* and *C27*/*C536* (mean c = 0.143 and 0.230, N = 38 and 26 progenies, respectively). The former two loci are 2.4Mb apart on chromosome 14, while the latter are 5.1Mb apart on chromosome 6 (Additional

file 4: Table S2). The summary recombination results are supported by results from the individual progenies. For example, values of c deviating from 0.5 at $p < 0.001$ (likelihood ratio test) were obtained for 54-100% of progenies for the five aforementioned marker pairs (98-100% for the three supergene-linked markers), but such extreme departures occurred in only 0-4% of progenies for all other marker pairs, with the great majority of pairs (97%) having none.

Estimates of the disequilibrium coefficient D^* and its statistical significance were obtained for inferred egg haplotypes (from embryos) and for the haploid male mates of the mother queens that produced the study progenies. For eggs, only the three supergene-linked marker pairs displayed significant disequilibrium (exact probabilities for all other marker pairs exceeded 0.315), with all three significant results withstanding correction for multiple comparisons. For males, eight marker pairs exhibited significant disequilibrium, with two of the supergene-linked marker pairs (*C294*/*Gp-9* and *Gp-9*/*i_126*) showing the lowest probabilities of equilibrium—only these latter two pairs retained statistical significance after correction for multiple comparisons.

(d) Progeny embryo segregation patterns

A pattern of significant TRD at the supergene but not other markers was inferred by several different analyses, including comparison of proportions of progenies with TRD between the two classes of markers as well as comparison of observed supergene segregation proportions with those expected by chance based on resampling analyses and explicit simulation models. Queens whose progenies exhibited significant distortion at the supergene-linked loci were not generally likely to produce eggs that departed from Mendelian segregation ratios at other loci. Specifically, the mean binomial probabilities of Mendelian ratios at the supergene loci in a progeny were not associated with the mean probabilities for the remaining segregating loci in that progeny ($n = 101$, Spearman $\rho = -0.006$, $p = 0.950$). Also, the 24 progenies implicated as displaying supergene-associated distortion did not, as a group, display a greater tendency for significant non-Mendelian ratios at the remaining segregating loci compared to the other 77 progenies (Mann-Whitney Test for differences in mean binomial probabilities, W = 1209, p =

0.454). Finally, a significant relationship was found between the mean binomial probability of Mendelian ratios across segregating loci in a progeny and the coefficient of variation (CV) for these probabilities (n = 101, Spearman ρ = 0.758, $p < 0.001$), implying a pattern in which queens with higher average levels of distortion also displayed higher variation across their loci. Together, these results agree with other evidence that queens producing eggs with significant supergene-linked TRD do not tend also to produce distorted ratios at other genomic regions.

Small groups of queenless workers maintained very high proportions of viable eggs/embryos in our rearing tests that supplemented the TRD analyses. Only one of 40 test units maintained fewer than 88% of the eggs/embryos initially given to them, and most units (23) kept all of their embryos viable for at least 48h (see Additional file 14: Figure S8). This result is important because it strongly suggests that worker culling of embryos based on embryo supergene status was unlikely to be the cause of significant supergene TRD in our progeny studies, based on the following. The formula for proportionate worker-induced selective mortality of embryos (M_{TRD}) that leads to biased segregation ratios is:

$M_{\mathrm{TRD}} = -([1/k]-2)/2$,

where k (unpolarized) is the proportion of gametes carrying the over-represented allele following selective worker cannibalism of embryos. Applying this formula using k values from our 24 study progenies with statistically significant supergene TRD yields estimates of expected losses of 20-34% of embryos in one-quarter of the supplementary test units, if workers actively discriminate against embryos on the basis of their supergene status. The marked disparity between such predicted high loss of embryos in a sizable subset of progenies compared to the low and largely consistent levels actually observed across our supplementary test units (Additional file 14: Figure S8) implicates actual segregation distortion, rather than selective worker cannibalism of embryos, as the primary or sole cause of significant TRD in our study progenies.

References

1. Shoemaker DD, Ross KG. Effects of social organization on gene flow in the fire ant *Solenopsis invicta*. Nature. 1996;383:613-6.
2. Lawson LP, Vander Meer RK, Shoemaker D. Male reproductive fitness and queen polyandry are linked to variation in the supergene *Gp-9* in the fire ant *Solenopsis invicta*. Proc R Soc London B. 2012;279:3217-22.
3. Krieger MJB, Ross KG, Chang CWY, Keller L. Frequency and origin of triploidy in the fire ant *Solenopsis invicta*. Heredity. 1999;82:142-50.
