# Supplementary material for: Unexpected patterns of segregation distortion at a selfish supergene in the fire ant Solenopsis invicta
Source: BMC Genet. 2018 Nov 7;19:101. doi: 10.1186/s12863-018-0685-9 (PMC6223060; doi:10.1186/s12863-018-0685-9)
Supplement: Supplementary file 7 — Table S3. Proportions of haplotypes with a supergene-associated allele that also bear such an allele at another supergene locus. (PDF 97.8 kb) [file 12863_2018_685_MOESM7_ESM.pdf]

Table S3 Proportions of haplotypes with a supergene-associated allele that also bear such an allele at another supergene locus

	Alleles		
	C294^{92}	*Gp-9*^{b}	*i_126*^{230}
Eggs			
C294^{92}	—	**0.991**<br>*0.498*<br>(*N*=62, *n*=973)	**0.942**<br>*0.495*<br>(*N*=55, *n*=589)
Gp-9^{b}	**0.992**<br>*0.498*<br>(*N*=62, *n*=972)	—	**0.951**<br>*0.501*<br>(*N*=85, *n*=1000)
i_126^{230}	**0.666**<br>*0.350*<br>(*N*=55, *n*=833)	**0.681**<br>*0.359*<br>(*N*=85, *n*=1396)	—
Males			
C294^{92}	—	**1.000**<br>*0.102*<br>(*n*=6)	**1.000**<br>*0.102*<br>(*n*=2)
Gp-9^{b}	**1.000**<br>*0.102*<br>(*n*=6)	—	**1.000**<br>*0.113*<br>(*n*=3)
i_126^{230}	**0.333**<br>*0.034*<br>(*n*=6)	**0.250**<br>*0.028*<br>(*n*=12)	—

Shown in bold are observed proportions of haplotypes with a supergene-associated allele indicated by the column heading that also bear another supergene-associated allele at a different supergene locus, indicated by the row label; proportions are shown separately for eggs (inferred from diploid embryo progenies of polygyne queens) and the haploid male mates of polygyne queens (inferred from diploid embryo progenies and their polygyne queen mothers). Proportions of each two-locus haplotype expected in the absence of linkage disequilibrium are shown in italics. Sample sizes (*N*, numbers of progenies for eggs; *n*, numbers of haplotypes) are shown in parentheses. Sample sizes are small for males because most of these mates of polygyne queens presumably originated from monogyne colonies (see text) and supergene-associated alleles are absent in the monogyne form
