# Supplementary material for: Unexpected patterns of segregation distortion at a selfish supergene in the fire ant Solenopsis invicta
Source: BMC Genet. 2018 Nov 7;19:101. doi: 10.1186/s12863-018-0685-9 (PMC6223060; doi:10.1186/s12863-018-0685-9)
Supplement: Supplementary file 13 — Text S3. Genetic mechanism of drive reversal―additional information. (PDF 135 kb) [file 12863_2018_685_MOESM13_ESM.pdf]

Text S3 Genetic mechanism of drive reversal—additional information

Variation observed in both the magnitude and direction of *Sb* supergene drive in polygyne *S. invicta* queens may depend on the genetic background, that is, on the genome-wide presence and nature of multiple segregating suppressors and enhancers of drive and their epistatic interactions [1-3]. One relatively simple scenario for *Sb* drive and its reversal posits multiple unlinked suppressor loci located on the *SB* haplotype of chromosome 16 and/or on other, non-homologous chromosomes; these suppressor loci hypothetically segregate allelic variants with additive effects to counter *Sb* drive, with both the strength and direction of drive determined by the proportion of such loci bearing the suppressor variants (see Additional file 15: Figure S9). Recombination at these unlinked loci (including independent assortment if they occur on different chromosomes) is expected to lead to a distribution of multilocus haplotypes that vary in their content of suppressor alleles (Additional file 15: Figure S9 inset). Specifically, if no or very few such loci bear a suppressor allele, then the additive countering effect is weak and *Sb* drive is expected to prevail. At the other extreme, if most or all such loci bear suppressor alleles, then the additive countering effect is so strong as to overwhelm the ability of *Sb* to drive and even cause reversal in the direction of transmission ratio distortion (TRD) promoted by the supergene. Intermediate proportions of loci with suppressor alleles (the most common circumstances, see figure inset) create a stalemate between the *Sb* drive and countering tendencies, which results in production of Mendelian segregation ratios. The nature of any such hypothetical suppressors of TRD involving true meiotic drive, as well as of any enhancers in the supergene, conceivably could involve regulating expansion/contraction of chromosome 16 centromeric repeat sequences, regulating levels of the kinetochore complex proteins associated with the chromosome 16 centromere, or influencing the development of meiotic spindle asymmetry involved in the orientation of selfish centromeres towards the egg pole [4-6].

Selection pressure on suppressors of TRD is expected to be most intense along the region of the homologous chromosome corresponding to the drive complex [7, 8], leading to the expectation

that suppressors are concentrated in, or confined to, such regions. Our estimates of nestmate queen genetic relatedness (*r*) reveal that queens displaying statistically significant TRD do not have especially low or high relatedness to their nestmate queens with TRD (see Additional file 6: Text S2; Additional file 12: Figure S7). Because we estimated *r* using only markers on chromosomes other than 16, a simple prediction of our model of drive reversal is that pairs of nestmate queens with TRD of different polarity (i.e., drive and drive reversal) should tend to have relatively low *r* while those with TRD of the same polarity should have relatively high *r*, if drive suppressors are located primarily outside the supergene homologous region. The absence of either pattern in our data suggests that such hypothetical modifiers are instead located in the supergene homologous region on the *SB* chromosome. Moreover, based on our analyses of all 101 progeny-producing queens, relatedness between any two nestmates was not predictive of congruence in their supergene *k* values; thus, extent of deviation from Mendelian ratios at the supergene does not appear to be highly heritable, a finding also consistent with hypothetical suppressors largely being confined to the supergene homologous region.

This simple additive genetic model can be used as a null model to test alternative hypotheses of the mechanism of drive reversal. For instance, one alternative hypothesis invokes historical recombination between linked driver and target loci that could produce recombinant haplotypes exhibiting rock-paper-scissors or other evolutionary dynamics, as well as drive reversal [e.g., 9]. Our finding of congruent deficiencies or excesses of supergene-linked alleles across all three of our supergene markers bracketing much of the supergene suggests that the requisite recombinant haplotypes are unlikely to result from single crossovers, assuming the drive locus falls in the interval between our markers. On the other hand, recombinants resulting from double crossovers—which generate gametes in which a middle segment is recombined while flanking markers remain unchanged—could dissociate drive and target loci but not affect the complete congruence among our markers in progenies with either drive or reversal-of-drive.

Similarly, the entire *Sb* drive complex (locus) potentially could be transferred into a *SB* haplotype background to yield drive reversal via a double crossover along the supergene sequence between any pair of our supergene markers (Additional file 16: Figure S10). However, this model has several stringent requirements. First, double crossovers appear to be very rare in large-inversion heterozygotes, on the order of 10^{-4}–10^{-5} crossovers per generation [10]. Second, the entire *Sb* drive locus would need to be included in the *SB* crossover product and, judging from the structure of other true meiotic drive elements [5, 11-13], it is likely to be a multi-megabase complex; significantly, no evidence exists for such large blocks of *Sb*-like sequence in a limited sample of *SB* chromosomes from the invasive range [14] nor in a large sample of *SB* chromosomes from the native range [15] (see Additional file 16: Figure S10b). Once integrated into the *SB* chromosome, the drive locus would be subject to dissociation of linked drive, responder, and any enhancer elements comprising it, because no protective inversions that act to limit such recombination exist on *SB* chromosomes in *S. invicta* [15] (Additional file 16: Figure S10c); proximity to the centromere could aid in maintaining the integrity of the complex by inhibiting recombination in the absence of *SB* inversions but, by the same token, such a location of the drive complex would pose an additional obstacle to the initial double crossover. Finally, the recombinant *SB* chromosome with the acquired drive locus presumably would need to be paired with a recombinant *Sb* chromosome that had lost it in order for supergene drive reversal to be manifested in a given progeny (Mendelian ratios are expected when queens are drive-locus^{+} or drive-locus^{-} homozygotes); therefore, mutiple drive locus recombinants and wild-type social chromosomes are predicted to circulate as standing genetic variation in wild polygyne *S. invicta* populations. Comparative analyses of the chromosome 16 sequences of queens from this study that displayed significant drive and drive reversal (or their eggs) will be useful for surveying for large recombinant blocks, as required by this model. The absence of such blocks suggested by current data is consistent with the additive null model.

References

1. Lyttle TW. Segregation distorters. Annu Rev Genet. 1991;25:511-57.
2. Christianson SJ, Brand CL, Wilkinson GS. Reduced polymorphism associated with X chromosome meiotic drive in the stalk-eyed fly *Teleopsis dalmanni*. PLoS ONE. 2011;6:e27254.
3. Reinhardt JA, Brand CL, Paczolt KA, Johns PM, Baker RH, Wilkinson GS. Meiotic drive impacts expression and evolution of X-linked genes in stalk-eyed flies. PLoS Genet. 2014;10:e1004362.
4. Fishman L, Saunders A. Centromere-associated female meiotic drive entails male fitness costs in monkeyflowers. Science. 2008;322:1559-62.
5. Didion JP, Morgan AP, Clayshulte AM-F, Mcmullan RC, Yadgary L, Petkov PM, et al. A multi-megabase copy number gain causes maternal transmission ratio distortion on mouse chromosome 2. PLoS Genet. 2015;11:e1004850.
6. Akera T, Chmátal L, Trimm E, Yang K, Aonbangkhen C, Chenoweth DM, et al. Spindle asymmetry drives non-Mendelian chromosome segregation. Science. 2017;358:668-72.
7. Jaenike J. Sex chromosome meiotic drive. Annu Rev Ecol Syst. 2001;32:25-49.
8. Larracuente AM, Presgraves DC. The selfish *Segregation Distorter* gene complex of *Drosophila melanogaster*. Genetics. 2012;192:33-53.
9. A Traulsen, FA Reed. From genes to games: Cooperation and cyclic dominance in meiotic drive. J Theor Biol. 2012;299:120-5.
10. Stevison LS, Hoehn KB, Noor MAF. Effects of inversions on within- and between-species recombination and divergence. Genome Biol Evol. 2011;3:830-41.
11. Axelsson E, Albrechtsen A, Van AP, Li L, Megens HJ, Vereijken ALJ, et al. Segregation distortion in chicken and the evolutionary consequences of female meiotic drive in birds. Heredity 2010;105:290-8.
12. Safronova LD, Chubykin VL. Meiotic drive in mice carrying *t*-complex in their genome. Russian J Genet. 2013;49:885-97.

13. Fishman L, Kelly JK. Centromere-associated meiotic drive and female fitness variation in *Mimulus*. Evolution. 2015;69:1208-18.
14. Pracana R, Priyam A, Levantis I, Nichols RA, Wurm Y. The fire ant social chromosome supergene variant *Sb* shows low diversity but high divergence from *SB*. Mol Ecol. 2017;26:2864-79.
15. Zheng Y. The evolution of a social supergene in fire ants. PhD thesis, Univ. Lausanne, https://serval.unil.ch/notice/serval:BIB_DB589E420FA0 (2018). Accessed 25 Aug 2018.
